# Axially Chiral Bifluorenylidene Radical Anions with Long Spin–Lattice Relaxation Times at Room Temperature in Fluid Solution

**DOI:** 10.1021/jacs.6c04102

**Published:** 2026-05-26

**Authors:** Brett M. Lucht, Marisa N. James, Nicholas A. Moriglioni, Elizabeth L. Fosnocht, Sunil Saxena, Wesley J. Transue

**Affiliations:** Department of Chemistry, 201934 University of Pittsburgh, Pittsburgh, Pennsylvania 15213, United States

## Abstract

Despite the recent interest in magnetochiral phenomena, well-characterized radicals that possess both molecular chirality and spin remain scarce. Herein, we report the synthesis of a series of axially chiral bifluorenylidene (BF) molecules and their singly reduced radical anions with varying degrees of fjord-region benzannulation. Our systematic structural perturbations provide insight into how the twist angle across their central C=C bond influences their redox state, electronic structure, stereochemical dynamics, and spin properties. Variable-temperature nuclear magnetic resonance (NMR) and electron paramagnetic resonance (EPR) studies are used to explore the barriers to major racemization processes. UV–vis–NIR absorption and magnetic circular dichroism spectroscopies provide insight into the electronic structures of the systems and are combined with multireference computational techniques to characterize the potential energy surfaces. Pulsed EPR measurements revealed that the BF radical anions displayed notably long spin–lattice (*T*_1_) relaxation times that approached 0.1 ms in fluid 2-methyltetrahydrofuran solution at room temperature, nearly 2 orders of magnitude longer than typical organic radicals. Remarkably, we found the unique combination of π delocalization, electronic structure, and low anisotropy insulates the spin from longitudinal relaxation due to molecular tumbling. The *T*_1_ temperature dependence is instead consistent with relaxation through thermally activated local-mode processes, suggesting that molecular design to control vibrations may be just as important in extending solution-phase *T*_1_ times as in the solid state. Our results establish BF radical anions as a versatile framework to explore spin–chirality interactions and to achieve long-lived spin states in nonviscous fluid solution at room temperature.

## Graphical abstract

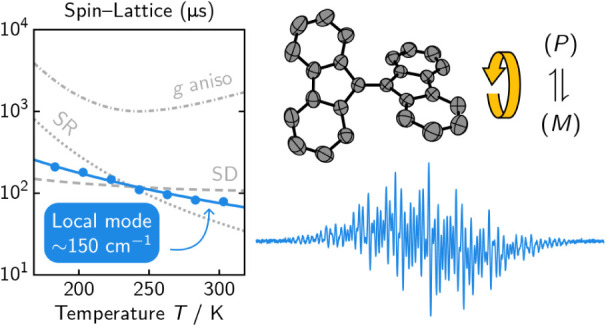

## Introduction

The interplay of spin and chirality has developed into a nexus of intense research activity. Fundamental questions remain about the link between these two phenomena, which arise due to breakdowns in parity and time-reversal symmetries.^1^ Recent years have seen exciting advancements in experimental demonstrations of molecular chirality-induced spin selectivity (CISS),^2^ magnetochiral photochemistry,^3^ field-sensitive enantioselective electrochemistry,^4^ and more.^5,6^ With such a burst of interest, it is essential to prepare and thoroughly characterize molecular systems with both unpaired electrons and well-defined molecular chirality in order to enable systematic studies of emergent magnetochiral phenomena.

We have identified bifluorenylidene (BF) molecules as an ideal framework for such investigations. Originally discovered in 1875,^7^ these molecules have long attracted attention due to the unusual twist across their central C=C bond that confers axial chirality, interesting photophysics, facile reduction, and complicated structural dynamics. BFs have found use in molecular conductance,^8^ organic photovoltaics,^9^ singlet fission,^10^ and more;^11^ yet, the singly reduced radical anionic forms have never been isolated. This is surprising because they exist at the precise intersection of spin, chirality, and dynamic stereochemistry that has recently captured such sustained attention.

Herein, we report the synthesis of a homologous series of BFs (Figure 1a) and their radical anions, representing the first isolation and comprehensive characterization of this class of chiral organic radicals. Barriers to their racemization processes are explored for both neutral and anionic BFs by variable temperature (VT) nuclear magnetic resonance (NMR) or electron paramagnetic resonance (EPR) spectroscopies. Combination of UV–vis–NIR absorption and magnetic circular dichroism (MCD) spectroscopies with computational techniques provides insight into the influence of reduction on the twisted C=C moiety and its electronic structure. Pulsed EPR measurements reveal exceptionally long spin–lattice *T*_1_ times approaching 0.1 ms in fluid solution at room temperature, nearly 2 orders of magnitude longer than those of typical organic radicals. While *T*_1_ times of molecules immobilized in solids or glasses are well studied, those in fluid solution are far less so, making the long *T*_1_ times of the BF radical anions particularly noteworthy. The contrast between the extended *T*_1_ times and the more typical phase memory times (*T*_m_ ∼ 0.5–1.2 μs) reveals an unexpected resilience to spin–lattice relaxation mechanisms while remaining susceptible to typical spin dephasing processes. Our results establish BF radical anions as a promising platform to explore the interplay of spin and chirality within molecular paramagnets, while also raising fundamental questions about the different solution-phase mechanisms responsible for spin–lattice *T*_1_ and spin–spin *T*_2_ relaxation.

**Figure 1:**
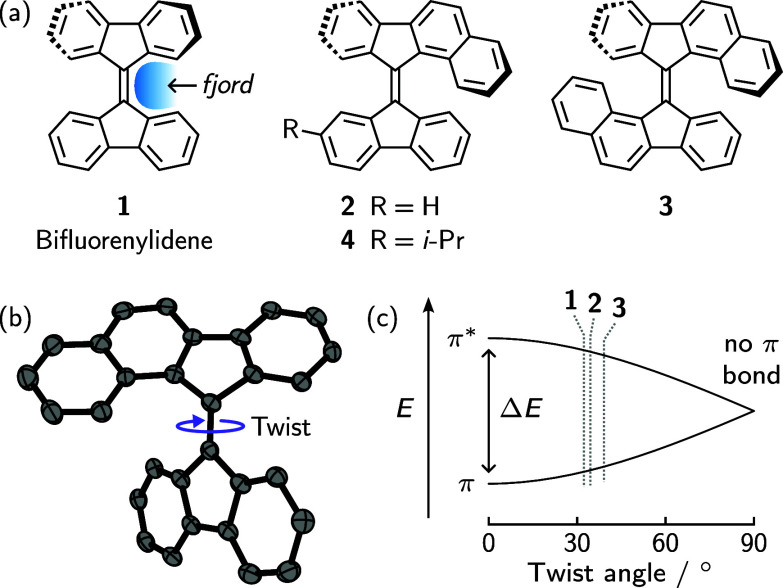
(a) Bifluorenylidenes have a “fjord” region, defined as a five-sided pocket within a polyaromatic hydrocarbon. Fjords cause steric clashing between internally directed positions and lead to dihedral twisting. (b) X-ray crystallography of **2** shows a twist of 33.6°. (c) Twisting reduces *p* orbital overlap and weakens the π interaction from its theoretical nontwisted strength Δ*E*.

## Results and Discussion

### Neutral Bifluorenylidene Compounds

We began by synthesis and characterization of the neutral BFs. Compounds **1**–**4** were prepared following modified literature procedures^12–14^ and isolated as brightly colored solids (orange **1**, red **2**/**4**, red-purple **3**) after purification by column chromatography or preparative thin-layer chromatography. ^1^H NMR analysis revealed *E*-to-*Z* stereoisomer ratios of **3** and **4** to be 94:6 and 40:60, respectively, indicating energy differences of Δ*G*_*E*→*Z*_(293 K) = +1.6 and −0.2 kcal/mol. X-ray crystallographic analysis was performed on **2** to quantify the twist angle across its central C=C bond (1.382(4) Å), revealing a 33.6(4)° twist angle (Figure 1b). This is intermediate between those known for **1** (31.9–33.0°)^15^ and **3** (39.1°),^13^ and it underscores the effectiveness of benzannulation within the fjord region to modulate the twist angle.^16^ Twisting an olefin reduces the strength of its C=C π interaction (Figure 1c), and this is reflected in several of the spectroscopic features of **1**–**3**. UV–vis–NIR absorption spectra showed a decrease in the π → π^*^ transition energies (*ṽ*_max_ 22 000 cm^–1^ **1**, 20 800 cm^–1^ **2**, 19 700 cm^–1^ **3**, Figure 3a), and Raman spectra showed a decrease in the energies of the symmetric C=C stretch (*ṽ* 1550 cm^–1^ **1**, 1536 cm^–1^ **2**, 1523 cm^–1^ **3**, Figure 3b).

Bifluorenylidenes are famous for the complicated dynamics of their stereochemistries around their twisted C=C bond.^17,18^ Four stereoisomers are possible (Figure 2), and the two major stereoisomerization processes, *E*/*Z* isomerization and edge passage, are both racemizing pathways. Direct *E*/*Z* isomerization would be unusual for a typical olefin: it proceeds through a biradical 90° transition state and is rarely observed due to prohibitively high barriers (e.g., 33–35 kcal/mol for tetraphenylethylenes).^19^ The barrier is dramatically lowered in BFs due to their inherent twist and electronic stabilization of the biradical transition state,^20^ making *E*/*Z* isomerization a thermally accessible process.

**Figure 2:**
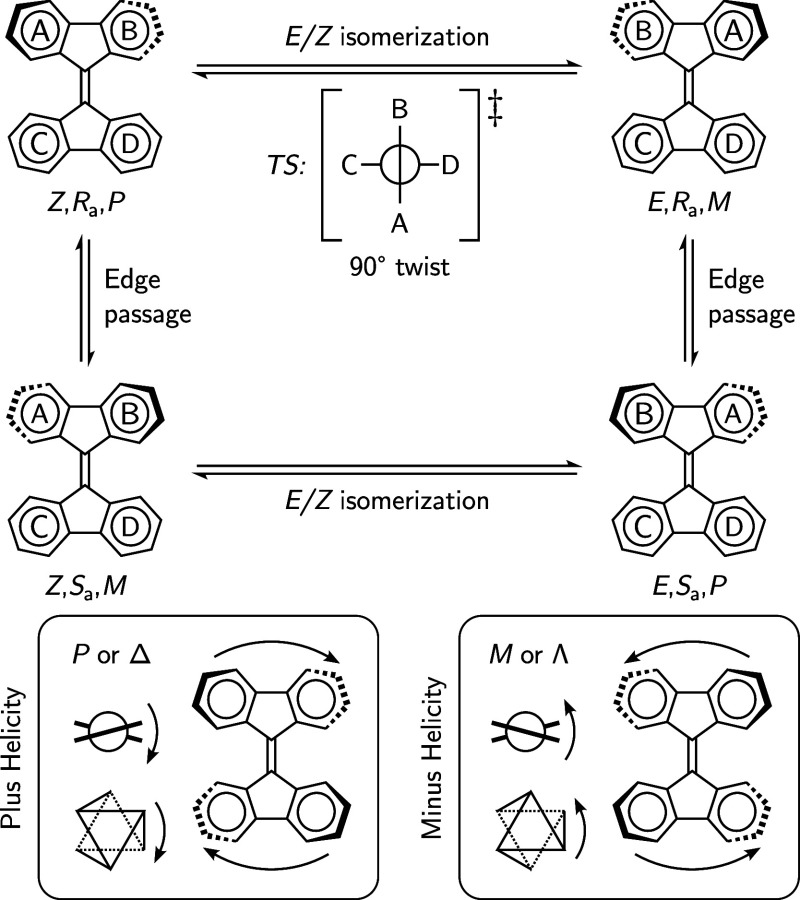
There are two primary pathways for stereochemical dynamics. Stereoisomers have been labeled with *E*/*Z* and *R*_a_/*S*_a_ assuming Cahn–Ingold–Prelog priorities A > B > C > D, and *P*/*M* according to the handedness of the helicity.

Analysis of **4** with a combination of exchange spectroscopy (EXSY) NMR studies and VT dynamic NMR (DNMR) studies allowed quantification of the barrier to *E*/*Z* isomerization as Δ*H* ^‡^ = 22.2(3) kcal/mol, Δ*S* ^‡^ = 2(1) cal/mol·K (Figure 3c). This barrier is lower than Δ*G* ^‡^(443 K) = 24.9 kcal/mol known for the nonbenzannulated 2,2^′^-dimethyl-9,9^′^-bifluorenylidene,^17^ demonstrating that benzannulation in the fjord region reduces the *E*/*Z* isomerization barrier. The diastereotopic CH_3_ protons of the isopropyl substituents of **4** also enabled measurement of the edge passage barrier, Δ*G* ^‡^(373 K) = 19.4 kcal/mol. This value is increased from that of 2-isopropyl-9,9^′^-bifluorenylidene, Δ*G* ^‡^(206 K) = 10.5 kcal/mol,^17^ showing that fjord benzannulation sterically inhibits edge passage. Both barriers of **4** remain low enough to preclude separation and isolation of its enantiomers at room temperature; however, the half-lives of these processes should allow for observation of enantioenrichment through steady-state spectroscopies.

**Figure 3:**
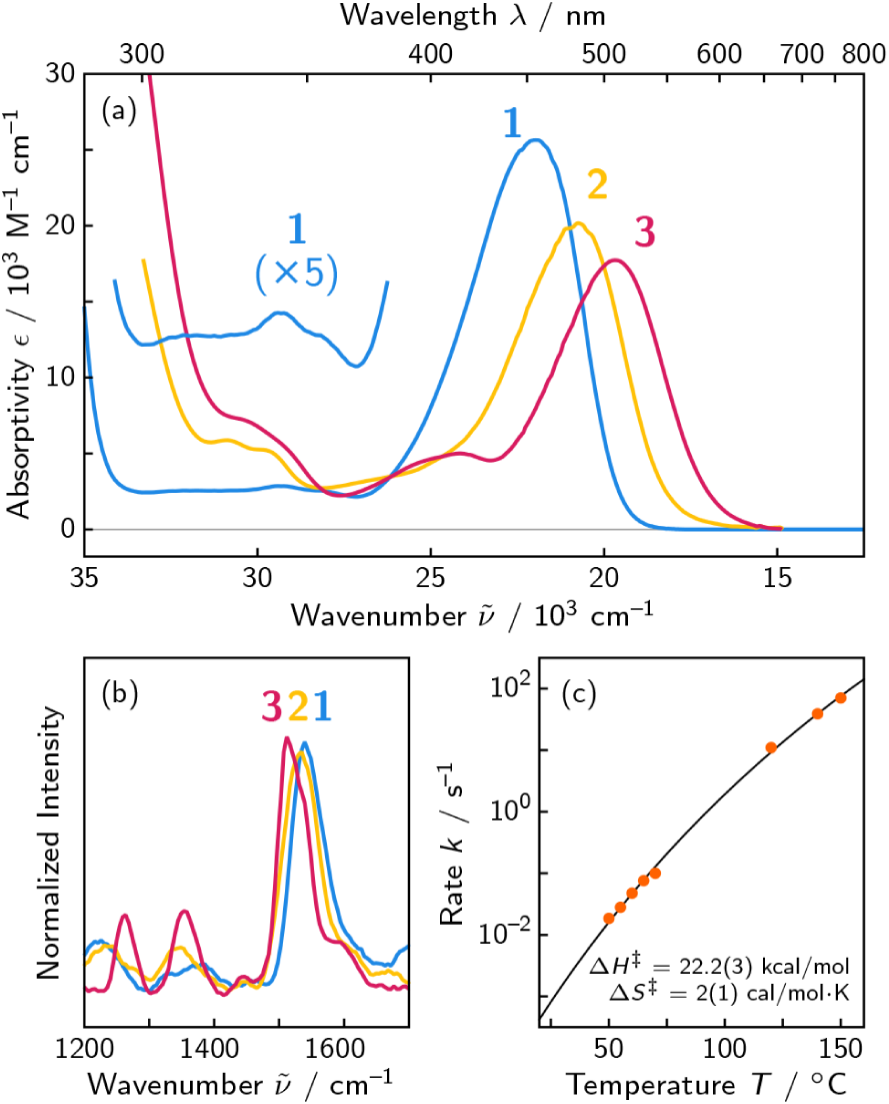
(a) Absorption spectra of neutral BF compounds **1**–**3** in THF solution show prominent π → π^*^ transitions in the 19–22 000 cm^–1^ region along with a series of weaker transitions in the 22–33 000 cm^–1^ region. (b) Raman spectra (excitation λ 532 nm) show a decrease in C=C stretch corresponding with increased twist angle. (c) Rate constants for *E*/*Z*-isomerization of **4** was measured by EXSY NMR (50–70 °C) and by DNMR line shape analysis (120–150 °C) to estimate a barrier.

A question naturally arises whether the changes in the geometric, spectroscopic, and kinetic properties of benzannulated BFs are due primarily to steric or electronic effects. The influence of the twist angle on the {π, π^*^}^2^ electronic excited states of **1** was explored using multireference CASSCF­(2,2)/RI-NEVPT2 calculations^21,22^ with ORCA 6.0.1 ^23^ (Figure 4a). These multireference calculations accurately captured the energy of the π → π^*^ transition to be 22 080 cm^–1^, and they showed that the energy of this transition should gradually fall as the twist angle approaches 90°. The 3 × 3 CI model^24^ was used to contextualize the calculated potential energy surface (PES). This model allowed us to describe each BF with an active space of two electrons in two *p* orbitals localized on each carbon (labeled “A” and “B”). Two-electron repulsion and exchange terms were parametrized as described in SI Section S5.3, and the one-electron energies of each *p* orbital were described as

1 $$H=\left(\begin{array}{cc} h_{AA} & h_{AB}\\ h_{AB} & h_{BB} \end{array}\right)$$

where *h*_*AA*_ is the energy of the *p* orbital on carbon “A”, *h*_*BB*_ is the same for carbon “B”, and *h*_*AB*_ is the mixing energy between the two *p* orbitals. We have assumed this mixing energy varies as *h*_*AB*_ = 1/2 Δ*E* cos ϕ, where ϕ is the twisting angle and Δ*E* is the energetic splitting between π and π^*^ orbitals when ϕ = 0° (Figure 1c). If differences in PESs are strongly influenced by increased delocalization upon benzannulation, this should be reflected in a decrease in Δ*E*. Fitting the 3 × 3 CI model to the calculated PESs gave Δ*E* = 20 600(200) cm^–1^ for **1**, 20 800(200) cm^–1^ for **2**, and 18 600(200) cm^–1^ for **3**. This shows no change between **1** and **2** in Δ*E* within the standard uncertainty of the fit, which contrasts with the experimental decrease in barrier between 2,2^′^-dimethyl-9,9^′^-bifluorenylidene^17^ (akin to **1**) and **4** (akin to **2**), ΔΔ*G* ^‡^(443 K) = −3.6 kcal/mol. This indicates the change in barrier is primarily driven by steric strain rather than an increase in delocalization. The lower value of Δ*E* for **3** suggests that increased benzannulation may gradually start be more influential on the barrier, as can be seen in some highly benzannulated literature examples.^16^

**Figure 4:**
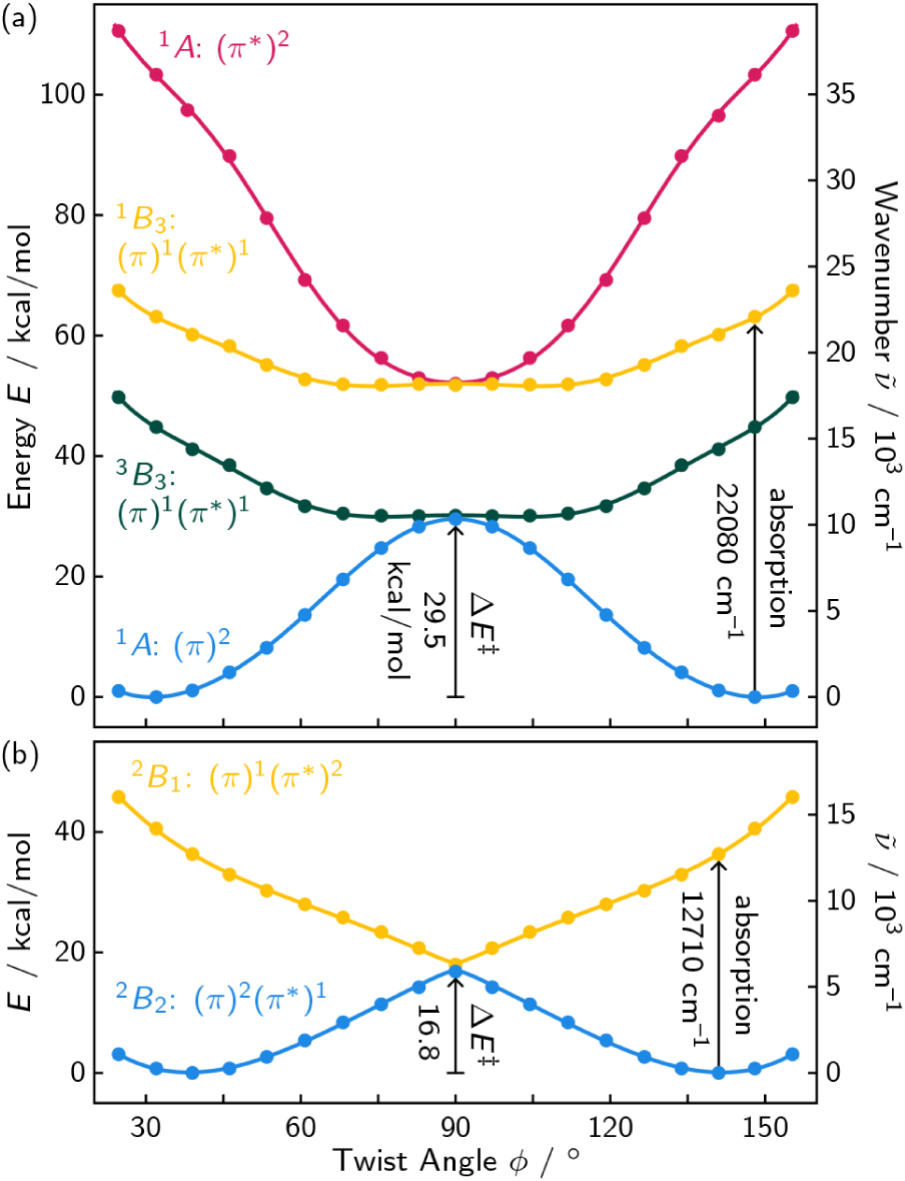
CASSCF­(*n*,2)/RI-NEVPT2 calculations show how the dihedral twists across the central olefinic bond of BFs relate to the UV–vis–NIR spectra and the *E*/*Z* isomerization barriers. The energies of the {π, π^*^}^*n*^ states for (a) **1** (*n* = 2) and (b) [**1**]^•–^ (*n* = 3) are plotted.

### Reduced Bifluorenylidene Radical Anions

Despite decades of studying BFs as electron acceptors, we are unaware of any reports of isolating BF radical anions, the closest example being a 1.74 *e*-reduced tetrafluorenofulvalene species (Figure 5a).^25^ The perceived inaccessibility of BF radical anions has even led some researchers to explore ultrafast pump–probe experiments on electronic excited states of neutral **1** as a surrogate for the anion.^26^ As such, we were pleasantly surprised to find that **1** could be easily reduced with potassium metal (1.1 equiv) in THF to yield dark green solutions of [**1**]^•–^. Recrystallization by slow vapor diffusion of hexane into THF at −35 °C provided needles of [K­(THF)_4_]­[**1**] in 53% isolated yield. Thus, [K­(THF)_4_]­[**1**] can be accessed in two steps from commercial materials. Reduction of **2** and **3** proceeded under similar conditions in THF to yield their dark purple radical anions.

**Figure 5:**
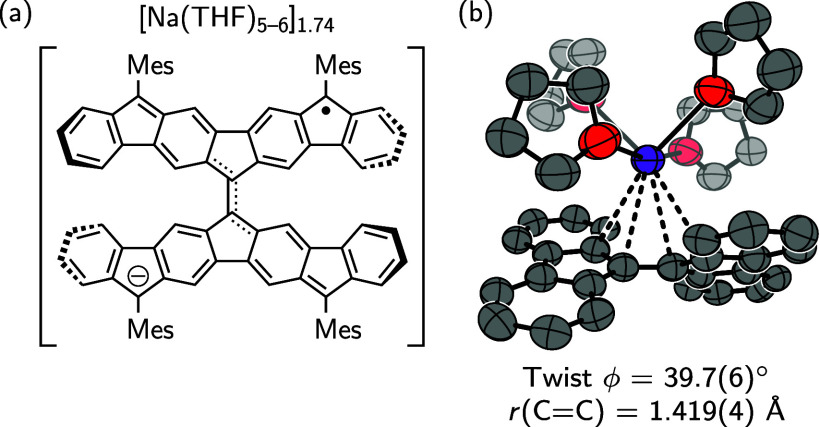
(a) The closest relevant example to an isolated reduced BF is this reduced tetrafluorenofulvalene species with a complicated electronic structure as described by Prajapati et al.^25^ (b) Our X-ray diffraction data for [K­(THF)_4_]­[**1**] revealed a 39.7(6)° twist angle and 1.419(4) Å olefinic C=C bond length.

These anions were highly sensitive to air and moisture. Crystalline samples of [K­(THF)_4_]­[**1**] could be stored for weeks in the freezer of our glovebox (−35 °C); however, even traces of O_2_/H_2_O in the glovebox atmosphere caused decomposition. This sensitivity hindered the selection and mounting of high-quality crystals for X-ray crystallographic analysis. Nonetheless, we were able to collect preliminary data sets on [K­(THF)_4_]­[**1**] (Figure 5b) and [K­(THF)_4_]­[**2**] (SI Section S4). The parent anion revealed a dihedral twist of 39.7(6)° and a central C=C bond length of 1.419(4) Å, showing a slight increase in both parameters relative to the neutral compound (avg. 1.37(1) Å, 32.4(2)°).^15^ The potassium cation rested symmetrically above the central olefinic unit, suggesting that this is the site of greatest accumulation of electron density in [**1**]^•–^. Similar metrics were observed in the reduced [K­(THF)_4_]­[**2**] species, which showed a twist angle of 45(1)° and a C=C bond length of 1.44(1) Å, both also increased from the neutral. Reduction should formally lower the olefinic bond order from 2 to 1.5, decreasing the driving force for planarity and thus allowing the anions to adopt larger twist angles. This appears to be the case from our crystallographic studies, which provided twist angles that parallel values calculated by DFT: [**1**]^•–^ 40.8°, [**2**]^•–^ 44.9°, [*E*-**3**]^•–^ 47.5°, and [*Z*-**3**]^•–^ 49.6° (ωB97X-D3/Def2-TZVP;^22,27–29^ see SI Section S5.1).

The electronic structures of the radical anions were interrogated by UV–vis–NIR absorption and magnetic circular dichroism (MCD) spectroscopies (Figure 6). The absorption spectra showed a prominent π → π^*^ transition in the NIR region that appeared to decrease in energy with increasing twist angle (*ṽ*_max_ 12 000 cm^–1^ [**1**]^•–^, 10 900 cm^–1^ [**2**]^•–^, 9 900 cm^–1^ [**3**]^•–^). Our CASSCF (3,2)/RI-NEVPT2 calculations (Figure 4b) captured the energies of these transitions well, and their predicted PESs could be fit using (eq 1) to show that the differences in these excitation energies are primarily driven by the twisting rather than delocalization. While the MCD spectra were complicated and showed a variety of peaks, they serve as a useful fingerprint for the species of interest. The MCD spectra of [**1**]^•–^ were unchanged by the identity of the counterion (Li, Na, K, Mg), indicating that it likely exists as solvent-separated ions^30^ in THF solution at room temperature (see SI Section S2.3).

**Figure 6:**
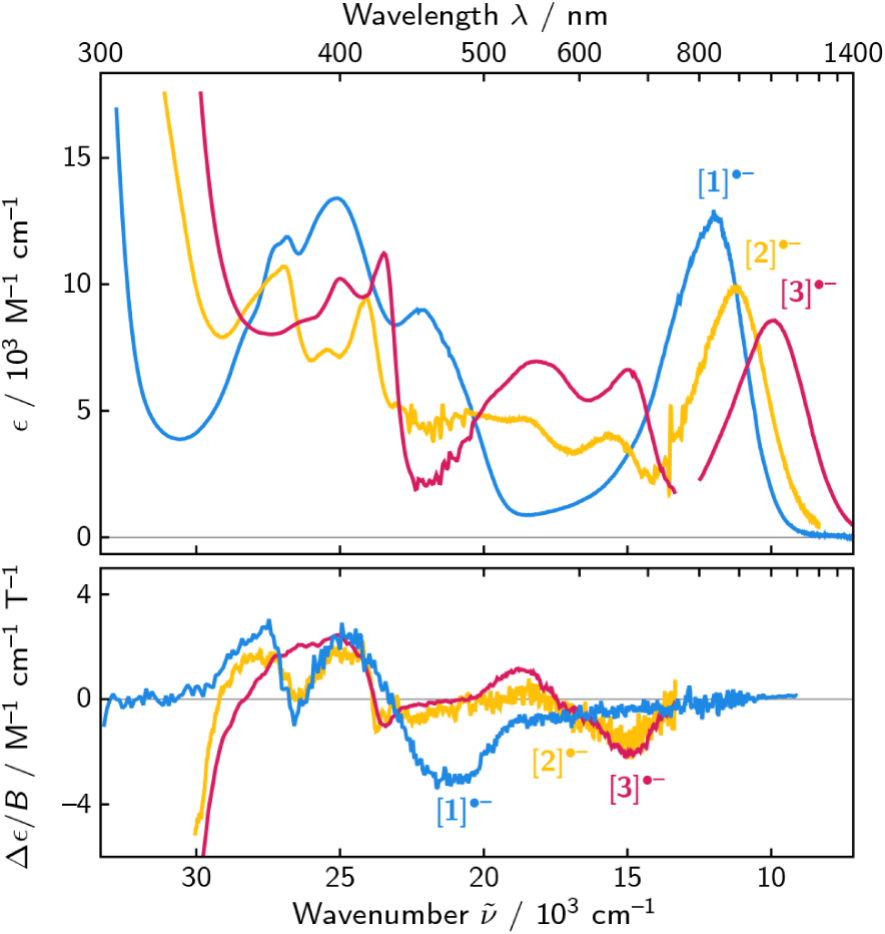
UV–vis–NIR absorption (*top*) and MCD (*bottom*) spectra of BF anions in THF solution at room temperature have a large number of features. Their π → π^*^ transitions appear squarely in the NIR region.

Solutions of [K­(THF)_4_]­[**1**] in 2-MeTHF at room temperature showed a beautifully complex X-band EPR spectrum (Figure 7).^33^ The presence of four proton environments with four protons each should yield a 5^4^ = 625-line pattern, and we were able to model its line shape using EasySpin^34^ with *g* = 2.0028 and isotropic hyperfine values of *A*_iso_ = 5.42, 4.34, 1.48, and 0.88 MHz. Neither benzannulated system exhibited such well-defined hyperfine structure. Anion [**2**]^•–^ had no resolved splitting, presumably because its 18 distinct proton chemical environments muddled the appearance. Some hyperfine structure is observed in the spectrum of [**3**]^•–^, which should have ten distinct proton environments, each with two equivalent protons. However, any interpretation is complicated by our expectation that both *E* and *Z* isomers should be present. VT EPR measurements over a 183–303 K temperature range did not give any appreciable changes in line shape that would indicate a chemical exchange process, so we presume the *E* ⇌ *Z* coalescence temperature is either appreciably below 183 K or above 303 K. Some initial line shape simulations using DFT-predicted hyperfine constants suggest a lower bound for the barrier of Δ*H* ^‡^ ≳ 9  kcal/mol (SI Section S3.2). Compiling our multireference PES with DFT vibrational calculations (SI Section S5.1.1) gives our best computational barrier estimate of Δ*H* ^‡^ = 11.8 kcal/mol and Δ*S* ^‡^= −2.3 cal/mol·K for [**3**]^•–^, which agrees with this experimental lower bound.

**Figure 7:**
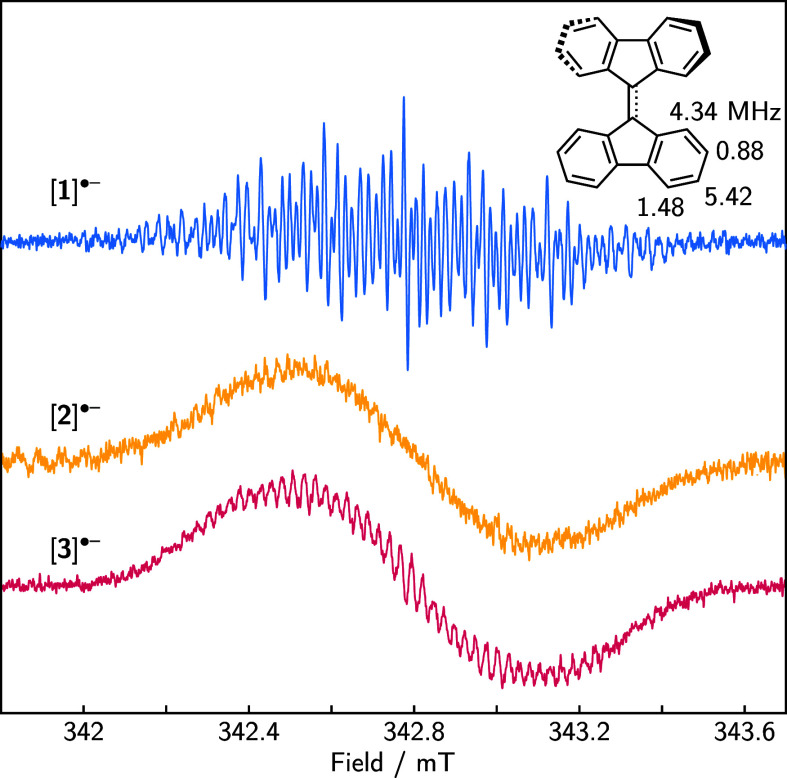
X-band CW EPR spectra for the three anions in 2-methyltetrahydrofuran solution (293 K) show varying degrees of resolved superhyperfine coupling. An intricate splitting pattern is seen in [**1**]^•–^, no coupling is resolved in [**2**]^•–^, and partially resolved splitting patterns in [**3**]^•–^. Fitting in EasySpin provided the *A* values shown for [**1**]^•–^. The assignments of *A* values to positions in [**1**]^•–^ were made by comparison with DFT calculations (PBE0/EPR-III).^31,32,54^

### Spin Relaxation Measurements

Recent measurements of molecular CISS,^2^ demonstrations of spin-controlled electrochemistry,^4^ and proposals for field-sensitive enantioenrichment^35^ all depend on a balance of time scales for electron transfer, enantioisomerization, and spin relaxation processes. We expect that the greatest insight into spin–chirality interactions will arise in systems where these three processes can be tuned to control their relative time scales. To this end, our estimate of the *E*/*Z* isomerization barrier in [**3**]^•–^ led us to explore BF spin dynamics through pulsed EPR experiments.

Extensive effort has been devoted to understanding solid-state electron spin relaxation,^36^ yielding both phenomenological (Debye) models^37^ and ab initio models^38^ that have allowed detailed studies of spin–environment interactions. The same cannot be said for fluid samples, where molecular tumbling introduces additional pathways that facilitate relaxation.^39^ Solution-phase relaxation studies have remained primarily within the domain of NMR rather than EPR spectroscopy. Performing analogous EPR studies is important because it is in fluid solutions where chiral radicals possess the thermal energy and conformational flexibility necessary for spin-sensitive isomerization processes. A deeper understanding of these relaxation pathways could also enable the development of chiral molecular quantum sensors that operate in homogeneous solution with their analytes, a key advantage of molecular systems over solid-state devices.^40^

Pulsed X-band EPR measurements of the BF anions revealed spin–lattice *T*_1_ times in fluid 2-MeTHF solution approaching 0.1 ms at room temperature (283–303 K, Figure 8). These *T*_1_ times are remarkably long for spins in nonviscous solution. For comparison, typical organic radicals under similar conditions have relaxation times 10^1–2^-times shorter at ambient temperature:^41^ DPPH *T*_1_ = 2.0 μs (toluene),^42^ thianthrene radical cation *T*_1_ = 0.4 μs (CF_3_COOH),^42^ and trityl radicals *T*_1_ ∼ 10–16 μs (water).^43^ While endohedral fullerene examples can exhibit longer *T*_1_ times (e.g., N@C_60_ *T*_1_ = 0.1–0.12 ms, CS_2_, 293 K),^44^ their extended relaxation is enabled by the unique screening of environmental interactions by the C_60_ cage, something that cannot be easily translated to nonfullerene systems. The *T*_1_ times for these BF anions are the longest examples that we have found for a nonfullerene molecular spin in nonviscous solution at room temperature. Notably, the phase memory times (*T*_m_ ∼ 0.5–1.2 μs) are not similarly extended, suggesting that specific *T*_1_ relaxation pathways have suppressed that differ from conventional *T*_m_ relaxation pathways.^36^

**Figure 8:**
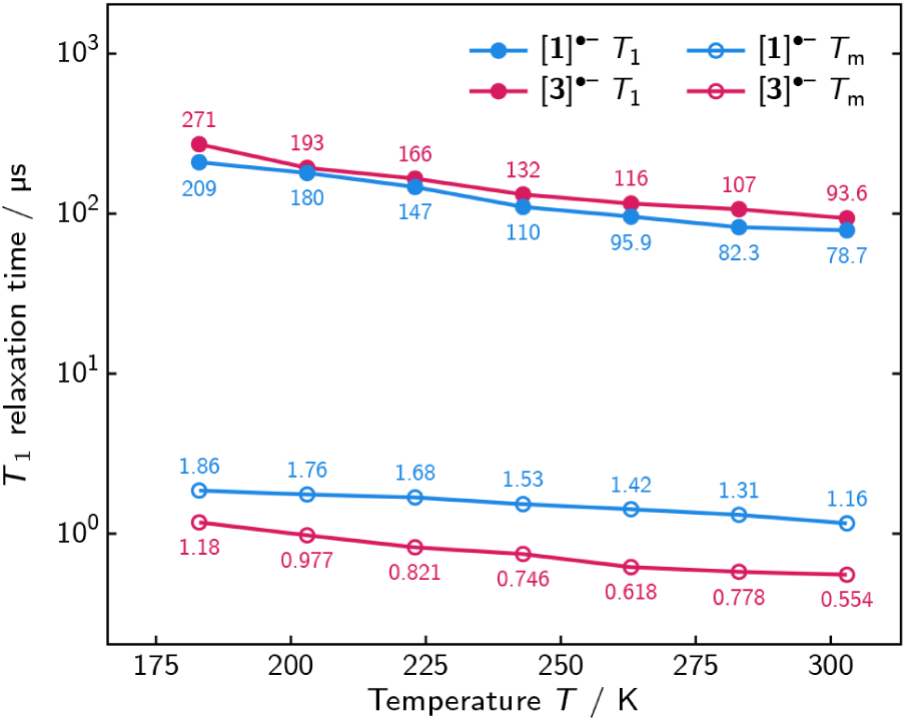
Spin–lattice (*T*_1_) and phase memory (*T*_m_) relaxation times of BF anions in 2-MeTHF solution were measured by inversion recovery and Hahn-echo experiments, respectively.

### Relaxation Mechanism Analysis

Spin–lattice relaxation in molecular solids is usually described as a combination of the direct, Raman, Orbach, and spin–phonon (local mode) mechanisms.^46^ Spins dissolved in nonviscous solution cannot usually be described using this model because molecular tumbling introduces additional spin relaxation mechanisms. There are many such mechanisms and their rates contribute additively,

2 $${T_{1}}^{-1}=\underset{\mathrm{process}}{\sum }{T_{1,\mathrm{process}}}^{-1}$$

Most directly, coupling between the electron spin of the molecule and its rotational angular momentum generates spin–rotational (SR) relaxation. This appears as a *J⃗CS⃗* term in the molecular Hamiltonian, where *J⃗* is the rotational angular momentum of the molecule and *C* is the spin–rotation interaction matrix. With a few assumptions, *C* can be related to the deviation of the molecular *g* values from the free electron (*g*_*e*_ = 2.0023) value,^39^ allowing us to calculate the SR contribution to *T*_1_ relaxation through

3 $${T_{1,\mathrm{SR}}}^{-1}=\underset{i=x,y,z}{\sum }\frac{{(g_{ii}-g_{e})}^{2}}{9\tau_{R}}$$

where τ_R_ is the rotational correlation time.^47^

Aside from direct coupling between spin and rotational angular momenta, the mobility of molecules in solution introduces several other relaxation mechanisms. Tumbling causes stochastic fluctuations in the effective *g* and hyperfine (*A*) values arising from modulation of their anisotropies. These mechanisms are called the *g* anisotropy and electron–nuclear dipolar (END) mechanisms, respectively, and follow^47^

4 $${T_{1,g}}^{-1}=\frac{2}{5}{\left(\frac{\omega }{g}\right)}^{2}\{\frac{1}{3}{(g_{zz}-\frac{g_{xx}+g_{yy}}{2})}^{2}+{(\frac{g_{xx}-g_{yy}}{2})}^{2}\}J\left(\omega \right)$$

5 $${T_{1,\mathrm{END}}}^{-1}=\frac{2}{9}I\left(I+1\right)\underset{i=x,y,z}{\sum }{(A_{ii}-\mathrm{A̅})}^{2}J\left(\omega \right)$$

where ω is the microwave frequency, *I* is the nuclear spin, *A̅* is the average (isotropic) *A* value, and $J\left(\omega \right)=\tau_{R}/\left(1+\omega^{2}\tau_{R}^{2}\right)$ is a spectral density function. Interaction with the nuclei of the solvent bath causes solvent diffusion (SD),^48^

6 $${T_{1,\mathrm{SD}}}^{-1}=R_{\mathrm{SD},\max}{\left[\frac{2\omega \tau_{R}}{1+{(\omega \tau_{R})}^{3/2}}\right]}^{1/4}$$

intermolecular exchange causes concentration-dependent relaxation,^49,50^

7 $${T_{1,\mathrm{exch}}}^{-1}=\kappa \left[R\right]$$

and thermal processes can arise from local mode vibrations or chemical exchange,^39,42,48,50^

8 $${T_{1,\mathrm{local}}}^{-1}=C_{\mathrm{local}}\frac{e^{\Delta /k_{B}T}}{{(e^{\Delta /k_{B}T}-1)}^{2}}$$

where *R*_SD,max_ is a proportionality constant for spin diffusion, κ is a constant characterizing Heisenberg exchange interactions, [*R*] is the radical concentration, Δ is the energy of a local mode, and *C*_local_ is the spin–vibrational coupling for the local mode.

The rotational correlation times of these anions were estimated using the Stokes–Einstein relation,

9 $$\tau_{R}=\frac{c_{\mathrm{slip}}V\eta }{k_{B}T}$$

where *V* is the molecular volume, *k*_B_ is the Boltzmann constant, *T* is temperature, and η is the temperature-dependent viscosity^51^ of 2-MeTHF (Figure 9a). We have estimated the molecular volumes of the BF anions to be 400–500 Å^3^ using a van der Waals sphere method as implemented in ORCA.^29^ The stick–slip *c*_slip_ parameter^52^ lies between 0 and 1 and characterizes solute–solvent friction: smaller values indicate the solute and solvent “slip” past each other, resulting in faster tumbling and shorter τ_R_, whereas larger values indicate they “stick” to each other, giving slower tumbling near the hydrodynamic limit. Values of *c*_slip_ ∼ 0.2–0.4 are typical of small organic radicals in medium-to-low polarity solvents with low viscosity (e.g., 0.2 for neutral aromatic radicals^42^ and 0.4 for tempone^53^ in toluene); however, we anticipate *c*_slip_ could be larger for [**1**]^•–^ and [**3**]^•–^ due to their anionic charge and the potential for ion pairing with their counterions to increase the effective molecular volume.

**Figure 9:**
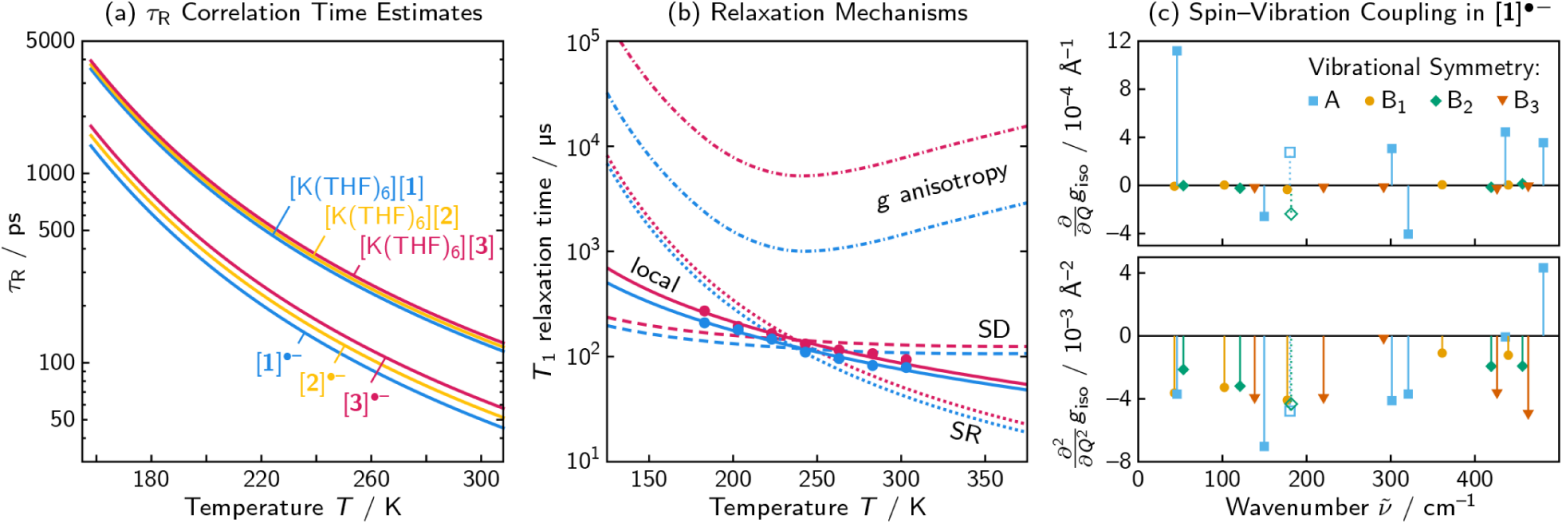
(a) Predicted rotational correlation times in 2-MeTHF estimated using the Stokes–Einstein relation. Molecular volumes were estimated with and without an associated K­(THF)_6_^+^ cation and are plotted with *c*_slip_ = 1 (SI Section 3.4.1). (b) Best-fit curves for the SR (dotted), SD (dashed), *g* anisotropy (dot-dashed), and local-mode (solid) mechanisms for [**1**]^•–^ (blue) and [**3**]^•–^ (red). The effective molecular volume *c*_slip_ *V*, the spin diffusion constant *R*_SD,max_, the local mode energy Δ, and the spin–phonon proportionality constant *C*_local_ were floated in these fitting routines. Combined SR+SD relaxation also provided a close fit but it resulted in unexpectedly small *c*_slip_ and *R*_SD,max_ values (SI Section 3.4.2). (c) First and second derivatives of the *g*_iso_ value calculated for each local mode (*Q*) of D_2_-symmetric [**1**]^•–^ are shown up to 500 cm^–1^ with their associated irreducible representation (irrep); the asterisk labels a strongly mixed pair of A and B_2_-symmetric modes. Only totally symmetric (A) modes are proficient at relaxing spin at first order,^45^ matching observation here. All D_2_ irreps follow Γ^2^ = A so the second derivatives can be nonzero regardless of irrep.

The temperature dependences of (eqs 3–9) provide each relaxation mechanism with a distinct temperature profile, allowing us to estimate which mechanisms contribute most strongly to the observed spin–lattice relaxation times. Remarkably, our analysis of these major relaxation pathways has shown that molecular tumbling and exchange are unlikely to dominate solution *T*_1_ relaxation (Figure 9b, SI Section 3.4). The SR and *g* anisotropy mechanisms are inefficient because of the combination of fast rotational correlation times (≲60 ps for the anions at 300 K, Figure 9a) and the small *g* value deviations (Δ*g* = *g* – *g*_*e*_ ≲ 0.0005). Such small *g* deviations arise from the weak spin–orbit coupling typical of hydrocarbon π systems (ca. 1 cm^–1^).^54^ Quantitatively, our best-fit analysis showed the *g* anisotropy mechanism cannot reproduce experiment, and the SR mechanism would require unreasonably small *c*_slip_ parameters of 0.08(1) for [**1**]^•–^ and 0.04(1) for [**3**]^•–^. The SD mechanism alone did not reproduce the temperature profile well, but performed much better in combined SR+SD fit; however, the fitted values of *c*_slip_ (∼0.15) and particularly *R*_SD,max_ (∼4000 s^–1^ versus the expected^48^ ∼10^5^ s^–1^) were judged to be too small (SI Section 3.4.2). The END mechanism was excluded because BF anions lack strong anisotropic hyperfine interactions such as the ^14^N coupling that dominates nitroxide radical relaxation.^47^ Finally, charged species like these BF anions are known to be less susceptible to relaxation through Heisenberg exchange in solution than neutral radicals.^41,50,55^

These estimates leave local mode (thermal) processes and modulation of END superhyperfine coupling as the likeliest contributors to *T*_1_ relaxation. Fitting the observed experimental *T*_1_ values to a single effective thermal process reproduced the temperature dependence well and yielded Δ = 120(70) cm^–1^ for [**1**]^•–^ and 190(50) cm^–1^ for [**3**]^•–^ (Figure 9b). Interestingly, DFT calculations reveal totally symmetric^45^ normal modes for [**1**]^•–^ at 150 and 181 cm^–1^ that have appreciable first-order spin–phonon coupling (Figure 9c).^45,56,57^ These calculated modes are within one standard uncertainty of both fitted values from experiment. Definitive assignment of local mode relaxation over superhyperfine END modulation would require sophisticated modeling of multispin electron–proton cross relaxation processes. As a simple test, a sum of (eq 9) over all DFT-calculated superhyperfine interactions of [**1**]^•–^ estimates an END contribution on the order of >10^6^ μs, too slow to dominate relaxation. However, we acknowledge this treatment drastically oversimplifies the spin dynamics, so future electron–electron double resonance (ELDOR) experiments will be needed to further confirm the dominance of local mode relaxation.^58^ Ultimately, our analysis suggests the long *T*_1_ times of these BF anions are consequences of their spin delocalization, rigid structure, anionic charge, and fast rotational correlation times, and we expect these will be useful design criteria when targeting long spin–lattice relaxation in solution.

## Conclusion

The tunability provided by fjord substitution patterns establishes BF molecules as useful models to study spin–chirality interactions. We have presented a systematic study of how fjord benzannulation impacts the electronic structures, olefinic twist angles, spectroscopic features, and stereochemical dynamics of both neutral and radical anionic BF species. We have also achieved the first isolation of a BF radical anion as an air-sensitive crystalline solid, enabling comprehensive characterization of this class of chiral radical previously thought to be inaccessible. Our results demonstrate that BF molecules and related axially chiral hydrocarbons represent ideal molecular frameworks to explore CISS and related magnetochiral phenomena.

The spin–lattice relaxation times of these anions are particularly notable, exceeding those of typical organic radicals in fluid solution by 10^1–2^ times and representing the longest *T*_1_ values reported for nonfullerene molecular spins in nonviscous solution at room temperature. Control and tunability of molecular spin relaxation times are highly desirable, especially now as the field works to uncover the complex interconnections between spin and chirality. The temperature dependence of the BF *T*_1_ times is well described by a single thermally activated local mode with energy around ∼150 cm^–1^, suggesting that synthetic targeting of specific vibrational modes can be just as important to extending relaxation times in solution as in the solid state.^59^ The contrast between the long *T*_1_ times and the conventional *T*_m_ times indicates that it may be necessary to independently optimize spin–lattice relaxation and spin decoherence in molecular paramagnets rather than focus on just one. Further studies will be necessary to map out the different *T*_1_ and *T*_m_ contributors in species like these BF anions. Our results indicate that long *T*_1_ times may be found in other reduced anionic π systems lacking strong hyperfine coupling, such as helicenes, coronenes, polycyclic aromatic hydrocarbons, and related systems.^60,61^ Studies of these classes of radical aromatic hydrocarbons will open new avenues for rational design of chiral molecular spins with tailored relaxation properties.

## Supplementary Material



## Notes

Experimental details, spectroscopic characterization, crystallographic information, computational details, and theoretical derivations (PDF)
