# Supplementary material for: Axially Chiral Bifluorenylidene Radical Anions with Long Spin–Lattice Relaxation Times at Room Temperature in Fluid Solution
Source: J Am Chem Soc. 2026 May 26;148(22):22921–30. doi: 10.1021/jacs.6c04102 (PMC13266708; doi:10.1021/jacs.6c04102)
Supplement: Supplementary file 1 [file ja6c04102_si_001.pdf]

Axially Chiral Bifluorenylidene Radical Anions with Long Spin–Lattice Relaxation Times at Room Temperature in Fluid Solution

Brett M. Lucht,[†] Marisa N. James,[†] Nicholas A. Moriglioni, Elizabeth L. Fosnocht, Sunil Saxena, Wesley J. Transue[*]

Supporting Information

Contents

S1 Synthesis **3**
S1.1 Neutral Bifluorenylidenes . . . 3
S1.1.1 Bifluorenylidene (**1**) . . . 3
S1.1.2 Benzo[*b*]fluorenone (**5**) . . . 4
S1.1.3 Mixture **6**: 2-(2-Naphthyl)benzoic Acid and 3-Phenyl-2-naphthoic Acid . . . 4
S1.1.4 Benzo[*a*]fluorenone (**7**) . . . 4
S1.1.5 9-Diazofluorene (**8**) . . . 6
S1.1.6 11-(9-Fluorenylidene)benzo[*a*]fluorene (**2**) . . . 6
S1.1.7 Bisbenzo[*a*]fluorenylidene (**3**) . . . 8
S1.1.8 2-Isopropylfluorenone hydrazone (**9**) . . . 8
S1.1.9 2-Isopropyl-9-diazofluorene (**10**) . . . 10
S1.1.10 11-(2-Isopropyl-9-fluorenylidene)benzo[*a*]fluorene (**4**) . . . 10
S1.2 Reduced Radical Anionic Bifluorenylidenes . . . 14
S1.2.1 Potassium Bifluorenylidene $[K(THF)_4]$[**1**] . . . 14
S1.2.2 Potassium Reduction of **2** and **3** . . . 18

S2 UV–Vis–NIR Absorption and MCD Spectroscopy **19**
S2.1 Data Acquisition and Processing . . . 19
S2.2 Absorption and MCD Spectra of Diamagnetic Species . . . 19
S2.3 Absorption and MCD Spectra of Paramagnetic Reduced Species . . . 20

S3 Electron Paramagnetic Resonance **22**
S3.1 Continuous Wave X-Band EPR Experiments . . . 22
S3.2 Simulation of Chemical Exchange in $[E/Z\text{-}\mathbf{3}]^{\bullet-}$ . . . 24
S3.3 Pulsed EPR Experiments . . . 24
S3.4 Discussion of Spin–Lattice Relaxation in Solution . . . 26
S3.4.1 Relaxation Processes and Rotational Correlation Times . . . 26
S3.4.2 Fitting Experimental T_1 Times . . . 27

S4 X-ray Crystallography **31**

S5 Computational Methods **33**
S5.1 Density Functional Theory (DFT) Calculations . . . 33
S5.1.1 Geometry Optimizations, Transition States, and Thermochemistry . . . 33
S5.1.2 Time-Dependent DFT Calculations . . . 35
S5.1.3 EPR Property Calculations . . . 40
S5.2 CASSCF/RI-NEVPT2 Multireference Calculations . . . 43
S5.2.1 CASSCF(n,2)/RI-NEVPT2 Dihedral Angle Excited State Scans . . . 43

S5.2.2 Larger CASSCF(n,8)/RI-NEVPT2 Calculations 43
S5.3 Discussion of Electronic Structure and the 3 × 3 CI Model 46
S5.4 XYZ Coordinates 52
S5.4.1 Neutral and Anion **1** 52
S5.4.2 Neutral and Anion **2** 55
S5.4.3 Neutral and Anion **3** 59

S1 Synthesis

Air-sensitive manipulations were performed in a MBraun LabMaster Pro SP glove box under an inert atmosphere of purified N_2 or using standard air-free Schlenk techniques. Solvents were obtained anhydrous and oxygen-free by bubble degassing (Ar), purification through alumina columns (Pure Process Technology Solvent Purification System), and storage in the glove box over 3 Å activated molecular sieves. Deuterated solvents were degassed and stored over molecular sieves for at least 2 days prior to use. Glassware was oven-dried for at least 2 h at temperatures greater than 150 °C.

Commercial reagents 1-indanone (TCI America), *o*-phthalaldehyde (TCI America), potassium hydroxide (Fisher Chemical), Eaton's reagent (Thermo Scientific), hydrazine hydrate (Thermo Scientific), activated manganese dioxide (technical grade, Alfa Aesar), Lawesson's reagent (Thermo Scientific), and benzo[a]fluorenone (TCI America) were purchased and used as received. Literature compounds fluorenone hydrazone[1] and 2-isopropylfluorenone[2] were prepared according to reported procedures.

All NMR spectra on diamagnetic compounds were obtained on Bruker Avance III (400, 500, or 700 MHz) instruments. New compounds were characterized by their ^{1}H and ^{13}C{^{1}H} spectra, and two-dimensional techniques (COSY, TOCSY, HSQC, HMBC) were used as needed when specific resonance assignments were important. ^{1}H and ^{13}C NMR chemical shifts (δ) in chloroform-*d* are reported in parts per million (ppm) relative to tetramethylsilane, using residual proton signals from the deuterated solvent as an internal reference.[3] ^{1}H NMR spectra in mesitylene-d_{12} were referenced to have the residual methyl solvent peak located at 2.161 ppm, which is the chemical shift of the mesitylene methyl resonance we measured in benzene-d_6. High resolution mass spectrometry (HRMS) were obtained on a Thermo Scientific Q-Exactive mass spectrometer through electrospray ionization (ESI); the mobile phase was acetonitrile spiked with formic acid. Raman spectra were acquired on solid samples with a Horiba XploRA PLUS confocal Raman microscope using a 532 nm laser.

S1.1 Neutral Bifluorenylidenes

S1.1.1 Bifluorenylidene (1)

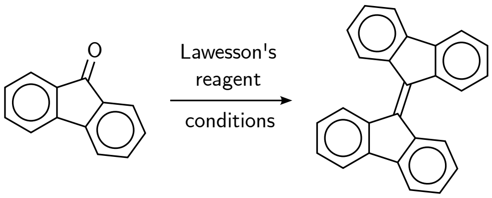

1

This preparation was adapted from literature reports for related compounds.[4,5] Inside of the glovebox, 9-fluorenone (5.00 g, 27.7 mmol), Lawesson's reagent (5.72 g, 14.1 mmol), dry toluene (100 mL), and a magnetic stir bar were added to a 250 mL Schlenk flask. The flask was sealed with a septum, brought outside the box, and attached to the Schlenk line. Under a strong counterflow of argon, the septum was swapped for a reflux condenser. The reaction mixture was heated to 115 °C under argon overnight with stirring for 16 hours. The dark red solution was dried in vacuo to a tacky red solid. The solids were boiled in 200 mL of ethanol, and filtered hot through a medium porosity frit. The filtrate was collected and cooled, which left behind large, bright orange needles of **1** (2.302 g, 51%). NMR analysis matched expectation from the literature. ^{1}H NMR (400 MHz, $CDCl_3$, 20 °C, Fig. S1) δ 8.39 (d, J = 7.9 Hz, 2H), 7.71 (d, J = 7.3 Hz, 2H), 7.33 (td, J = 7.5, 0.9 Hz, 2H), 7.21 (td, J = 7.9, J = 1.1 Hz, 2H). Raman (λ_{exc} 532 nm, Fig. 3b) 1550 cm^{-1}. UV–vis absorption (300–800 nm, THF, Fig. S19a) λ / nm [ϵ / M^{-1} cm^{-1}] 341 [2 840], 355 [2 560], 450 [25 600].

S1.1.2 Benzo[b]fluorenone (5)

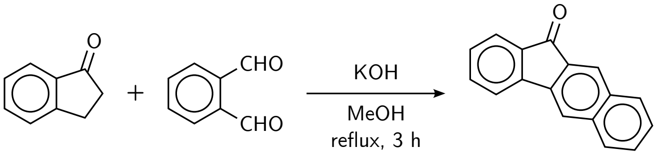

5

This prep was adapted from literature.[6] A solution of 1-indanone (10.0 g, 75.7 mmol) in methanol (50 mL) was added to a rapidly stirring suspension of *o*-phthalaldehyde (9.99 g, 75.7 mmol) in MeOH (50 mL). After stirring 30 min, a methanolic 28% KOH solution (6.72 g in 24 mL) was added slowly to the pale yellow solution, causing evolution of heat and the development of an orange color. The mixture was heated to reflux for 3 h, during which time the supernatant turned brown-red and a yellow solid precipitated. After cooling, the yellow solids were collected by filtration and washed with small portions of methanol to give 6.95 g (51%) of **5** as bright yellow solids. ^{1}H NMR (400 MHz, $CDCl_3$, 20 °C, Fig. S2) δ 8.16 (s, 1H), 7.88 (d, J = 8.0 Hz, 1H), 7.85 (s, 1H), 7.82 (d, J = 8.1 Hz), 7.75 (d, J = 7.4 Hz, 1H), 7.70 (d, J = 7.5 Hz, 1H), 7.59–7.52 (m, 2H), 7.46 (td, J = 7.49, 1.2 Hz, 1H), 7.34 (td, J = 7.4, 0.9 Hz, 1H).

S1.1.3 Mixture 6: 2-(2-Naphthyl)benzoic Acid and 3-Phenyl-2-naphthoic Acid

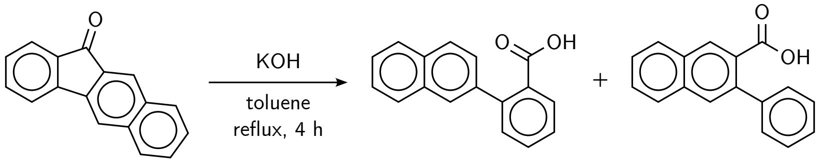

5 **6a** **6b**

Mixture **6**

This prep was adapted from literature.[6] A 250 mL round bottom flask was charged with yellow 11*H*-benzo[*b*]fluoren-11-one **5** (5.45 g, 24.7 mmol), KOH (16.7 h, 297 mmol) and a stir bar. Toluene (100 mL) was added and the mixture was refluxed (120 °C, 4 h), during which time the solution turned brown and orange-brown solids precipitated. After cooling, the reaction mixture was poured into water (200 mL) and the organic layer separated from the aqueous layer. The organic layer was then extracted with 10 wt% KOH (3 × 100 mL) in a separatory funnel. The combined aqueous extracts were cooled in an ice bath and acidified with 6 M HCl until the orange color disappeared to leave a colorless precipitate and a yellow supernatant. Product was extracted with diethyl ether (3 × 100 mL), the organic extracts were combined, dried over $MgSO_4$, and then volatiles were removed under reduced pressure to yield 4.28 g of a 60:40 mixture of 2-(2-naphthyl)benzoic acid **6a** and 3-phenyl-2-naphthoic acid **6b**. ^{1}H NMR ($CDCl_3$, 400 MHz, 20 °C, Fig. S3) δ 7.99 (d, ^4J = 4.5 Hz), 7.95 (d, ^2J = 7.7 Hz), 7.87–7.80 (m), 7.63–7.54 (m), 7.51–7.26 (m).

S1.1.4 Benzo[a]fluorenone (7)

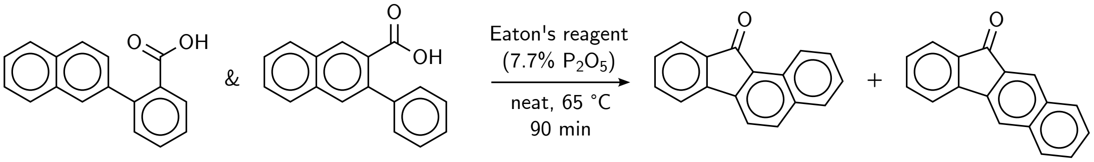

6a **6b** **7** **5**

Mixture **6**

This prep was adapted from literature.[6] A 250 mL round bottom flask was charged with the mixture **6** (9.77 g, 39.4 mmol) and a magnetic stir bar. Addition of Eaton's reagent (150 mL, 945.2 mmol) caused the yellow mixture to darken to brown-red, and the mixture was heated (65 °C) for 90 min. The dark suspension was then cooled and slowly added to an equal volume of DI water, which caused immediate precipitation

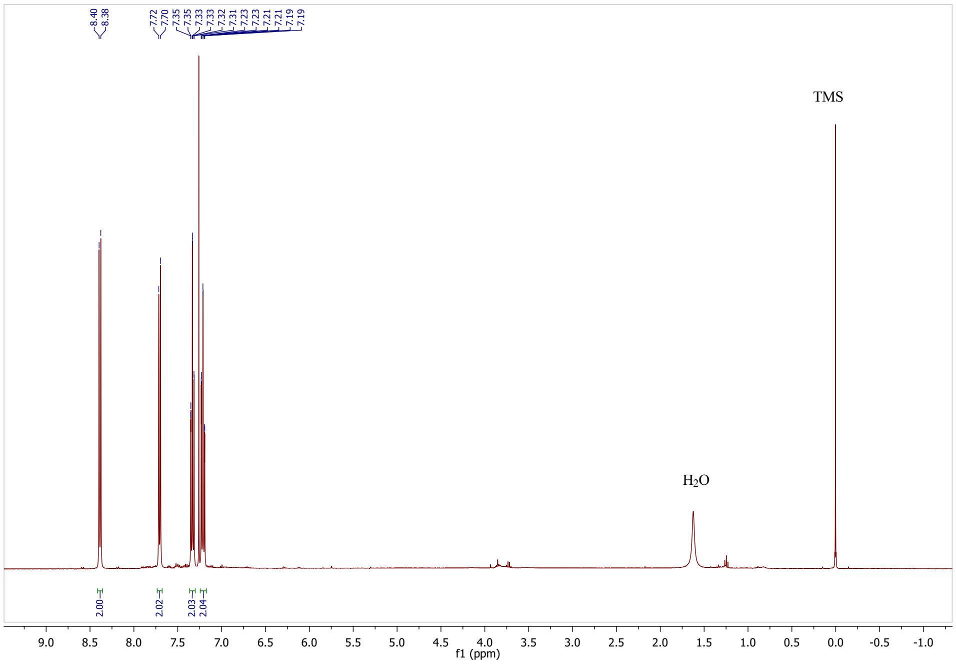

Figure S1: ^{1}H NMR spectrum of **1** in chloroform-*d*.

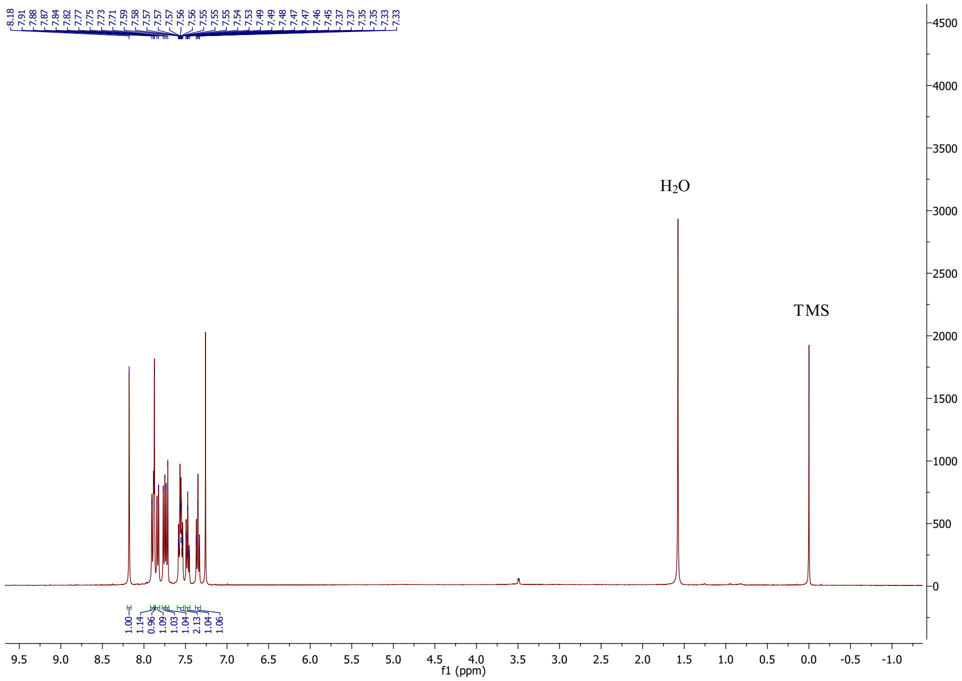

Figure S2: ^{1}H NMR spectrum of **5** in chloroform-*d*.

of yellow solids. This suspension was stirred for 30 min, precipitating additional brown-orange solids. The mixture was extracted with ethyl acetate (3 × 150 mL), and the combined organic extracts were washed with a saturated Na_2CO_3 solution (2 × 150 mL) followed by a washing using NaCl brine (1 × 150 mL). The organic phase was dried with $MgSO_4$, filtered, and the volatiles removed under reduced pressure to yield 9.40 g of a 60:40 ratio of benzo[*a*]fluorenone **7** to benzo[*b*]fluorenone **5**. This mixture was separated by column chromatography (95:5 hexanes:ethyl acetate), and **7** eluted as the first colored fraction. ^{1}H NMR (500 MHz, $CDCl_3$, 20 °C, Fig. S4) δ 8.96 (d, J = 8.4 Hz, 1H), 8.00 (d, J = 8.2 Hz, 1H), 7.79 (d, J = 8.3 Hz, 1H), 7.65 (d, J = 8.7 Hz, 1H), 7.62–7.57 (m, 2H), 7.49 (d, J = 7.3 Hz, 1H), 7.44 (t, J = 7.4 Hz, 2H), 7.28–7.25 (m, 1H).

S1.1.5 9-Diazofluorene (8)

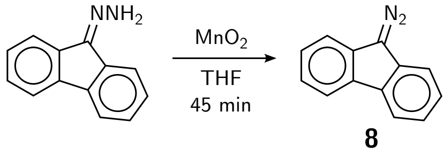

8

This prep was adapted from literature.[7] We have found this oxidation step using activated MnO_2 to be quite sensitive to the quality, age, and storage conditions of the MnO_2 oxidant. A 100 mL round bottom flask was loaded with 9*H*-fluoren-9-hydrazone (1.00 g, 5.10 mmol), activated MnO_2 (2.24 g, 25.7 mmol), THF (unstabilized, 50 mL), and a magnetic stir bar. The flask was wrapped in foil and stirred open to air for 45 min, which caused a light red color to develop. The mixture was filtered through a pad of Celite on a medium porosity frit, and the red filtrate was dried under reduced pressure to yield 0.807 g (82%) **8** as a pale red solid. ^{1}H NMR (500 MHz, $CDCl_3$, 20 °C, Fig. S5) δ 7.95 (d, J = 7.6 Hz, 2H), 7.52 (d, J = 7.7 Hz, 2H), 7.39 (td, J = 7.7, 1.1 Hz, 2H), 7.33 (td, J = 7.5, 1.0 Hz, 2H).

S1.1.6 11-(9-Fluorenylidene)benzo[a]fluorene (2)

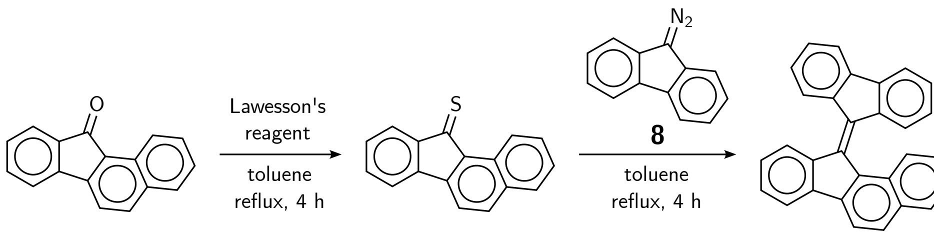

7 **2**

This prep was adapted from literature.[6] Inside of a nitrogen filled glovebox, a 100 mL three neck round bottom flask was loaded with **7** (197 mg, 0.86 mmol), Lawesson's reagent (346 mg, 0.86 mmol), toluene (15 mL), and a magnetic stir bar. A side neck of the flask was sealed using a gas inlet adapter, and the remaining two necks were sealed using rubber septa. The flask was removed from the box and attached to the Schlenk line. Positive argon pressure was used to swap the septum on the central neck for an argon-flushed condenser. The mixture was heated with magnetic stirring in an oil bath (120 °C, 4 h), causing the initial yellow mixture to turn dark red. After 4 h, a solution of **8** (173 mg, 0.90 mmol) in dry toluene (20 mL) was taken up into a syringe and injected at a rate of 2 mL/min using a syringe pump, maintaining the 120 °C oil bath temperature. Stirring was continued an additional 6 h. The solution was cooled to room temperature and passed through a 2 cm pad of silica. The filtrate was dried down to dark red solids which contained a mixture of **1**, **2**, **3**, and **7**. This mixture was separated by loading small portions (∼150 mg) of the dark red solids in concentrated DCM solution onto silica preparative thin-layer chromatography plates. Each plate was run using 4:1 hexanes:dichloromethane as the mobile phase and each plate was run three times, allowing the silica to dry fully between runs. The red band was scraped from each plate and material desorbed using four 2 mL rinses of DCM. The combined filtrates were dried to yield 108 mg (33%) **2** as a dark red solid. ^{1}H NMR (400 MHz, $CDCl_3$, 20 °C, Fig. S6) δ 8.43 (d, J = 7.9 Hz, 1H), 8.40 (d, J = 7.8 Hz, 1H), 7.99 (d, J = 8.1 Hz, 1H), 7.92 (d, J = 8.2 Hz, 1H), 7.89–7.83 (m, 2H), 7.76 (d, J = 7.5 Hz, 1H), 7.70 (d, J = 7.5

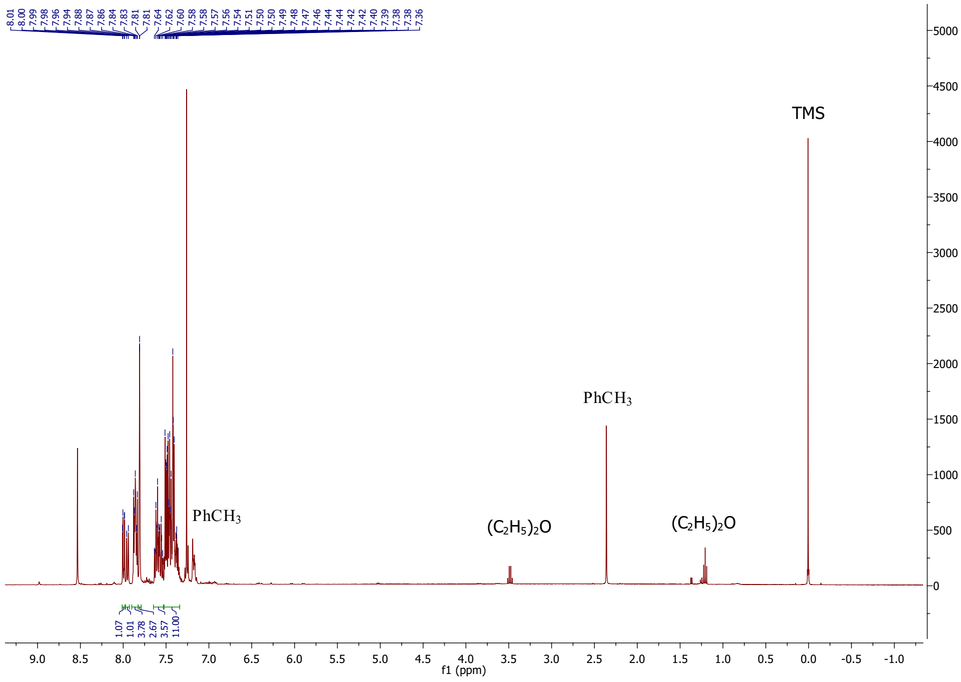

Figure S3: ^{1}H NMR spectrum of mixture **6** in chloroform-d.

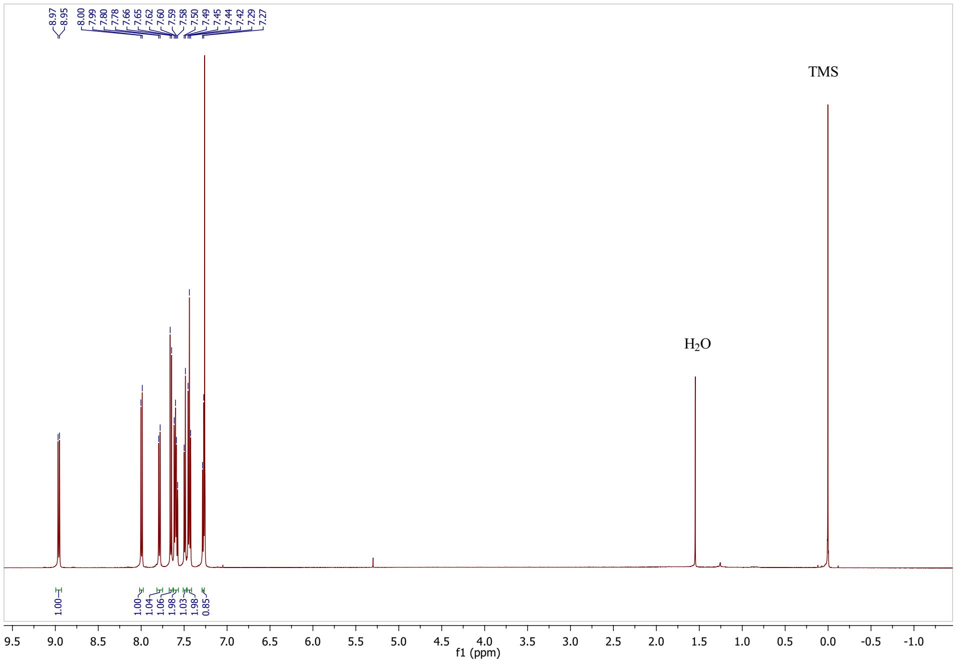

Figure S4: ^{1}H NMR spectrum of **7** in chloroform-d.

Hz, 1H), 7.67 (d, J = 7.4 Hz, 1H), 7.40 (td, J = 7.4, 0.97 Hz, 1H), 7.35–7.29 (m, 2H), 7.21 (m, 3H), 7.15 (td, J = 7.37, 1.05 Hz, 1H), 6.88 (td, J = 7.64, 1.14 Hz, 1H). Raman (λ_{exc} 532 nm, Fig. 3b) 1536 cm^{-1}. UV–vis absorption (300–800 nm, THF, Fig. S19a) λ / nm [ϵ / M^{-1} cm^{-1}] 324 [5 870], 336 [5 210], 381 [3 450 sh], 407 [4 960 sh], 482 [20 200].

S1.1.7 Bisbenzo[a]fluorenylidene (3)

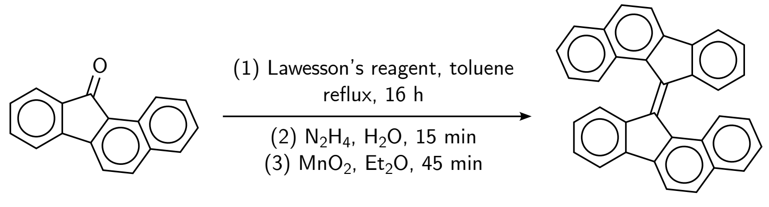

7 **3**

Inside of a nitrogen filled glovebox, a 50 mL Kjeldahl-style Schlenk flask was loaded with **7** (117 mg, 0.51 mmol), Lawesson's reagent (103 mg, 0.25 mmol), toluene (15 mL), and a magnetic stir bar. The flask was sealed with a rubber septum and removed from the box where it was placed under positive Ar pressure and its septum was swapped for an Ar-flooded condenser. The mixture was heated with magnetic stirring (120 °C, 16 h), causing all solids to dissolve and a dark red color to develop. The flask was raised from the oil bath and cooled to room temperature. The rubber septum was removed under a strong Ar counter-flow and hydrazine hydrate (0.20 mL, 4.1 mmol) was added quickly before sealing again. The solution was allowed to stir for 15 min and its color changed from dark red to pale yellow. The volatiles were removed in vacuo leaving pale yellow tacky solids. The solids were sonicated in diethyl ether (50 mL) and filtered through a pad of Celite. Activated MnO_2 (454 mg, 5.22 mmol) was added to the filtrate and stirred open to air for 45 min, then the mixture was filtered through Celite to give a deep purple-red solution containing both **3** and **7**. These were separated by silica gel column chromatography (9:1 hexanes:toluene), collecting the deep purple band. Drying yielded 49 mg (45%) of **3** as deep purple solids. ^{1}H NMR (500 MHz, $CDCl_3$, 20 °C, Fig. S7) δ 8.31 (d, J = 8.4 Hz, 2H), 7.97 (d, J = 8.3 Hz, 2H), 7.92 (d, J = 8.3, 2H), 7.68 (d, J = 7.4 Hz, 2H), 7.38 (td, J = 7.0, 1.28 Hz), 7.33 (td, J = 7.75, 1.2 Hz, 2H), 7.20 (d, J = 7.9 Hz, 2H), 7.16 (t, J = 7.4 Hz, 2H), 6.86 (td, J = 7.3, 0.7 Hz, 2H). Raman (λ_{exc} 532 nm, Fig. 3b) 1523 cm^{-1}. UV–vis absorption (300–800 nm, THF, Fig. S19a) λ / nm [ϵ / M^{-1} cm^{-1}] 331 [7 050], 342 [5 490], 392 [4 240], 414 [5 010], 507 [17 700].

S1.1.8 2-Isopropylfluorenone hydrazone (9)

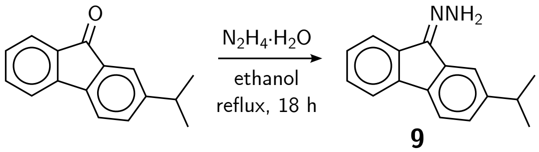

9

A 25 mL round bottom flask was charged with 2-isopropyl-9-fluorenone (55 mg, 0.25 mmol), ethanol (5 mL), and a magnetic stir bar, and was combined at room temperature. To this, hydrazine hydrate (0.10 mL, 2.0 mmol) was added. The flask was fitted with a reflux condenser and heated to reflux overnight. After cooling to room temperature, volatiles were removed, leaving behind a yellow oil (52.1 mg, 88% yield). ^{1}H NMR analysis revealed a ~3:1 ratio of two isomers, which we have labeled with an asterisk (*) for the major isomer and a prime (′) for the minor isomer. This was used in the next step without further purification. ^{1}H NMR (500 MHz, $CDCl_3$, 20 °C, Fig. S8) δ 7.88 (d, J = 7.6 Hz, 1H′), 7.76 (s, 1H′), 7.71 (dd, J = 7.4, 3.1 Hz, 2H′), 7.67 (d, J = 7.8 Hz, 1H′), 7.61 (t, J = 8.3 Hz, 2H*), 7.57–7.52 (m, 2H*), 7.41 (t, J = 7.5 Hz, 1H′), 7.36 (t, J = 7.4 Hz, 1H*), 7.34–7.26 (m, 2H*), 7.21 (dd, J = 7.8, 1.4 Hz, 1H′), 6.36 (d, J = 6.6 Hz, 2H*+2H′), 2.98 (m, 1H*+1H′), 1.31 (dd, J = 8.7, 7.0 Hz, 6H*+6H′). ^{13}C NMR (126 MHz, $CDCl_3$, 20 °C, Fig. S9) δ 149.34, 149.21, 149.03, 146.20, 146.16, 146.14, 145.94, 141.68, 140.26, 139.42, 138.86, 138.02, 137.88, 136.69, 130.79, 130.54, 129.87, 129.10, 128.62, 127.99, 127.58, 127.46, 127.44, 127.36, 127.28, 125.66, 125.17, 124.15, 123.28,

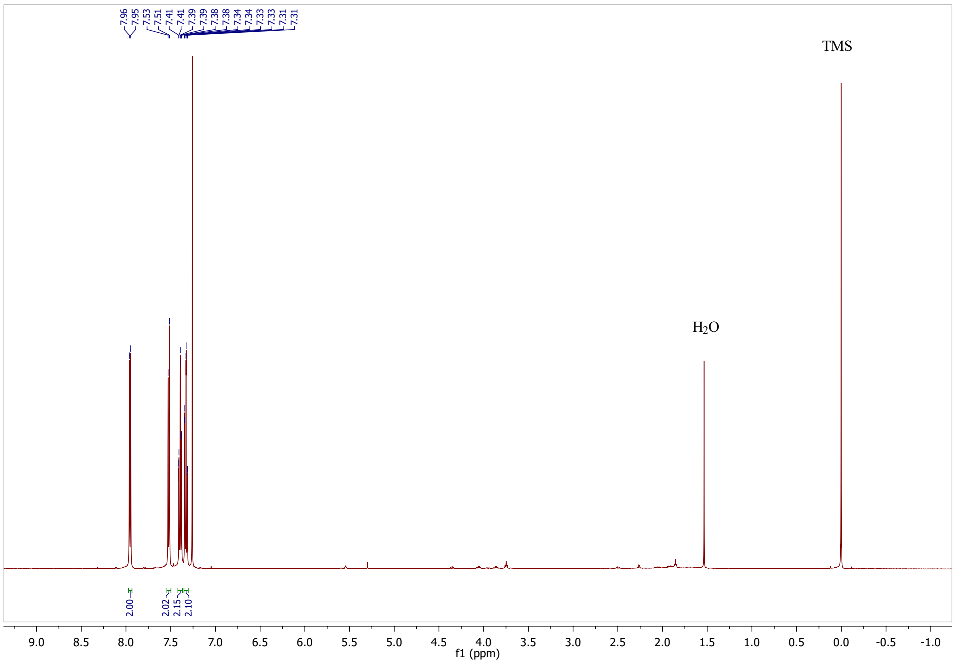

Figure S5: ^{1}H NMR spectrum of **8** in chloroform-*d*.

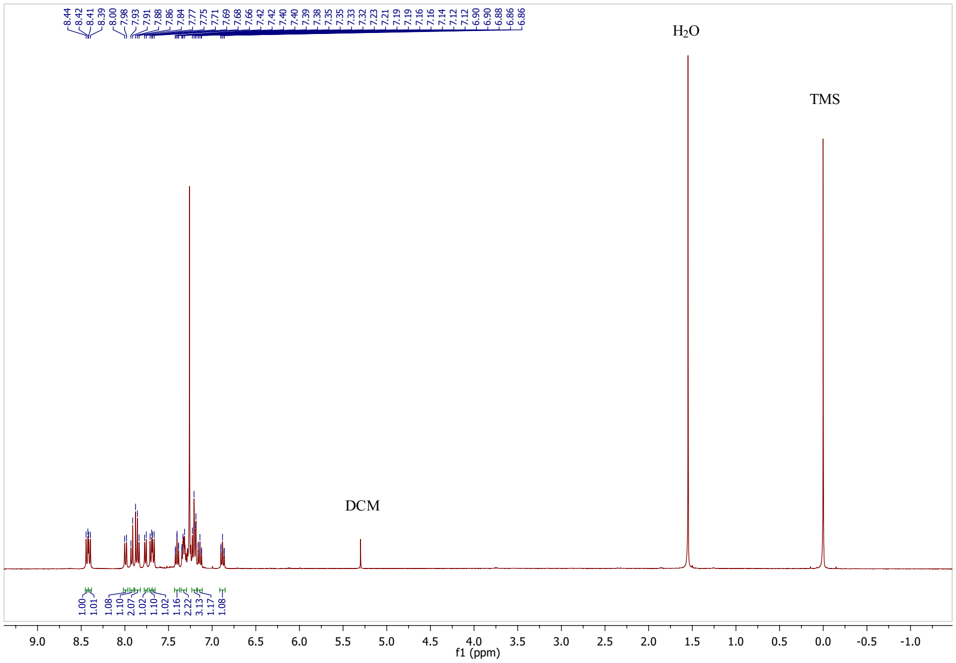

Figure S6: ^{1}H NMR spectrum of **2** in chloroform-*d*.

120.85, 120.50, 120.33, 119.93, 119.79, 119.57, 119.35, 118.69, 75.35, 34.68, 34.55, 34.44, 24.31, 24.29, 24.21, 24.18. HRMS (FTMS + p ESI) m/z: $[M+H]^+$ calculated for $C_{16}H_{16}N_2$, 237.1313; found, 237.13838.

S1.1.9 2-Isopropyl-9-diazofluorene (10)

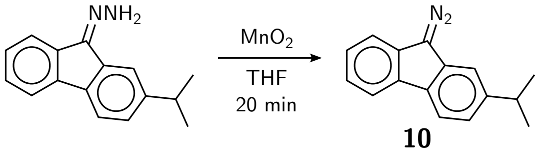

10

A 25 mL round bottom flask was loaded with **9** (52.1 mg, 0.23 mmol), activated MnO_2 (0.11 g, 1.23 mmol), stabilized THF (5 mL), and a magnetic stir bar. The mixture was stirred open to air for 20 min, causing a light pink color to develop, then it was filtered through a pad of Celite in a medium porosity frit. The pink filtrate had its volatiles removed under reduced pressure to yield a pink oil (46.5 mg, 88% yield) of crude **10**. The material obtained was impure, but decomposed on silica so it could not be further purified. This was used in the next step without further purification.

The unidentified peaks in the ^{1}H NMR spectrum were present in every attempt to make this compound in varying intensity. When these unidentified peaks were more intense than the peaks corresponding to **10**, a brown oil was obtained that could not be successfully used in the next step. By overlaying these various ^{1}H NMR spectra, we were able to determine that the integrated peaks in Fig. S10 do represent **10**, and the others represent an impurity that hinders the next step. These impurities also likely contribute to the low yield of **4**. ^{1}H NMR (500 MHz, $CDCl_3$, 20 °C, Fig. S10) δ 7.90 (d, J = 7.5 Hz, 1H), 7.86 (d, J = 7.9 Hz, 1H), 7.49 (d, J = 7.7 Hz, 1H), 7.38–7.34 (m, 2H), 7.32–7.28 (m, 1H), 7.20 (d, J = 7.9 Hz, 1H), 3.04 (sept, J = 6.9 Hz, 1H), 1.34 (d, J = 6.9 Hz, 6H).

S1.1.10 11-(2-Isopropyl-9-fluorenylidene)benzo[a]fluorene (4)

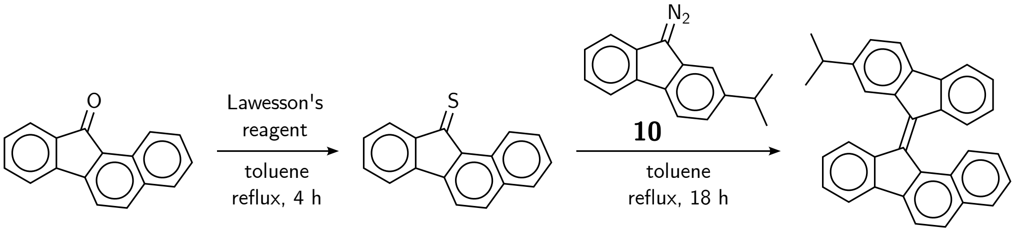

7 **4**

Inside of a nitrogen filled glovebox, a 50 mL three neck round bottom flask was loaded with Lawesson's reagent (58 mg, 0.15 mmol), toluene (8 mL) and a magnetic stir bar. A side neck of the flask was sealed using a gas inlet adapter, and the remaining two necks were sealed using rubber septa. The flask was removed from the box where it was placed under positive argon pressure and had one neck's septum swapped for an argon flooded condenser. Compound **7** (33 mg, 0.15 mmol) was added under a positive flow of argon. The mixture was heated with magnetic stirring in an oil bath (110 °C, 4 h), causing the initial yellow mixture to become dark red. After 4 h, a solution of **10** (46.5 mg, 0.17 mmol) in dry toluene (8 mL) was taken up in a syringe and injected at a rate of 2 mL/min using a syringe pump. The flask was kept at the same temperature for the duration of the addition plus an additional 18 h. The solution was then cooled to room temperature and passed through a short pad of silica. The filtrate was dried down to a dark red oil. Silica gel chromatography (4:1 hexanes:dichloromethane), followed by recrystallization from hexanes afforded a red solid (6.2 mg, 10% yield). ^{1}H NMR includes actual integrations when the peak likely corresponds to the minor isomer.

^{1}H NMR (700 MHz, $CDCl_3$, 20 °C, Fig. S11) δ 8.46 (dd, J = 7.8, 4.6 Hz, 1.8H), 8.39 (d, J = 7.7 Hz, 1H), 8.29 (s, 0.7H), 8.03 (d, J = 8.6 Hz, 0.8H), 7.99 (d, J = 8.5 Hz, 1H), 7.92 (d, J = 8.2 Hz, 2H), 7.88 (d, J = 8.2 Hz, 2H), 7.85 (t, J = 6.8 Hz, 2H), 7.73–7.70 (m, 2.8H), 7.66 (d, J = 7.8 Hz, 1H), 7.62 (d, J = 7.4 Hz, 1H), 7.56 (d, J = 8.2 Hz, 1H), 7.38 (t, J = 7.4 Hz, 1H), 7.35–7.26 (m, 5H), 7.23–7.12 (m, 6H), 7.06 (d,

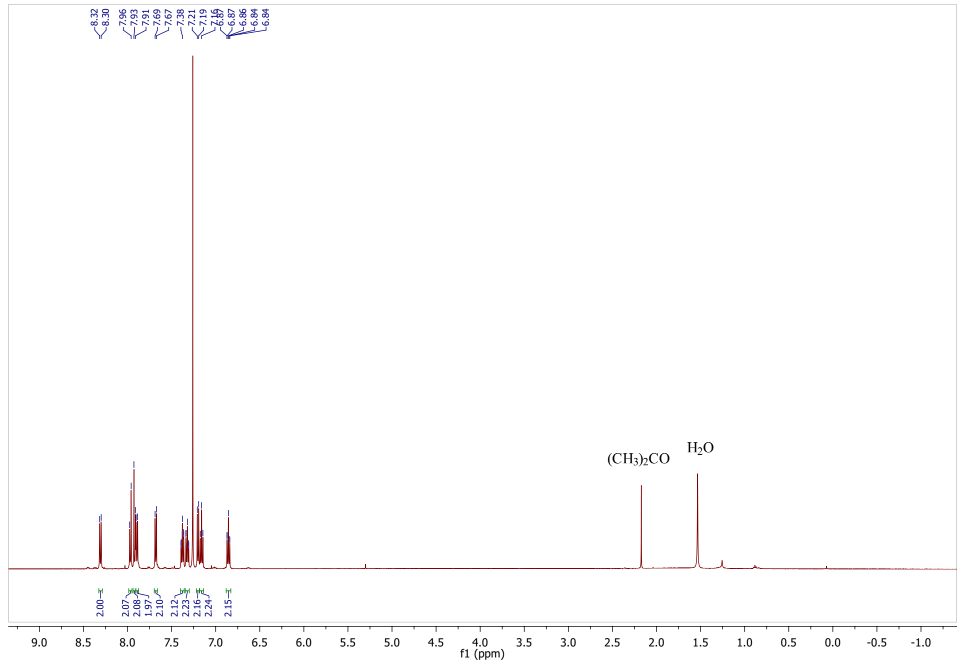

Figure S7: ^{1}H NMR spectrum of **3** in chloroform-*d*.

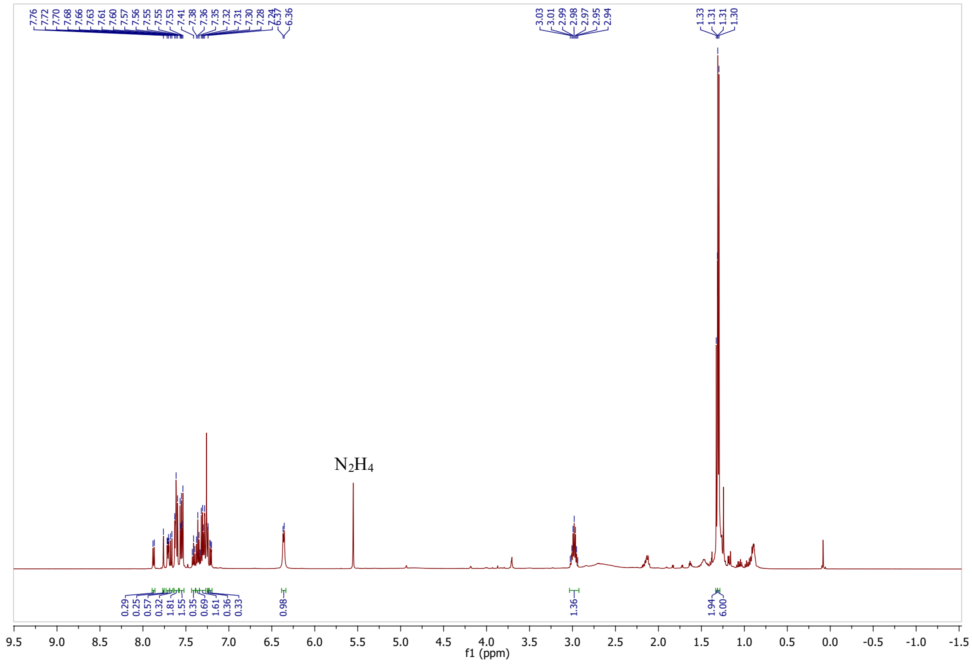

Figure S8: ^{1}H NMR spectrum of **9** in chloroform-*d*.

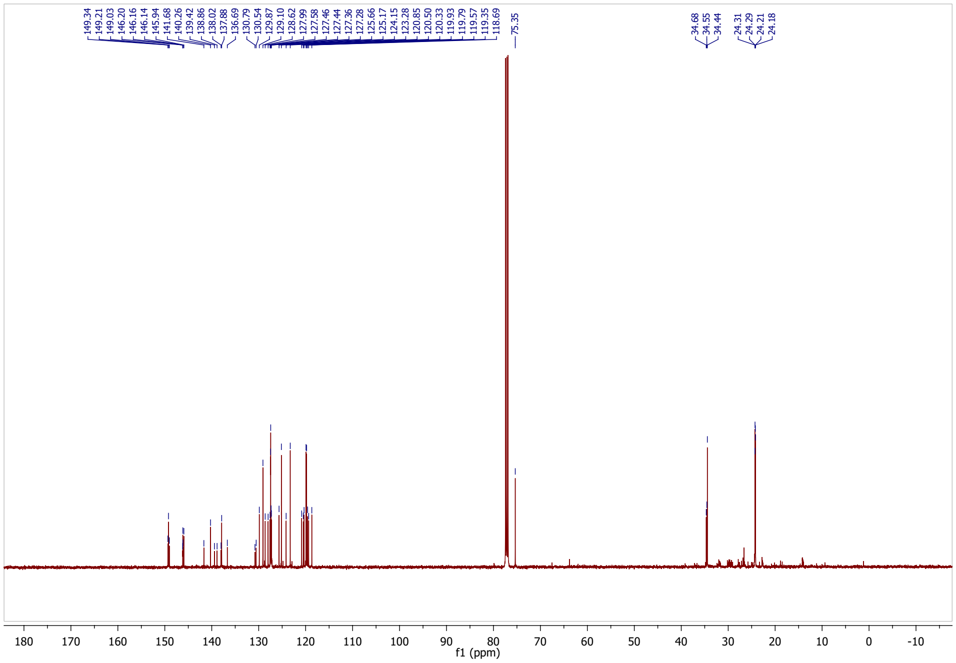

Figure S9: $^{13}C\{^{1}H\}$ NMR spectrum of **9** in chloroform-*d*.

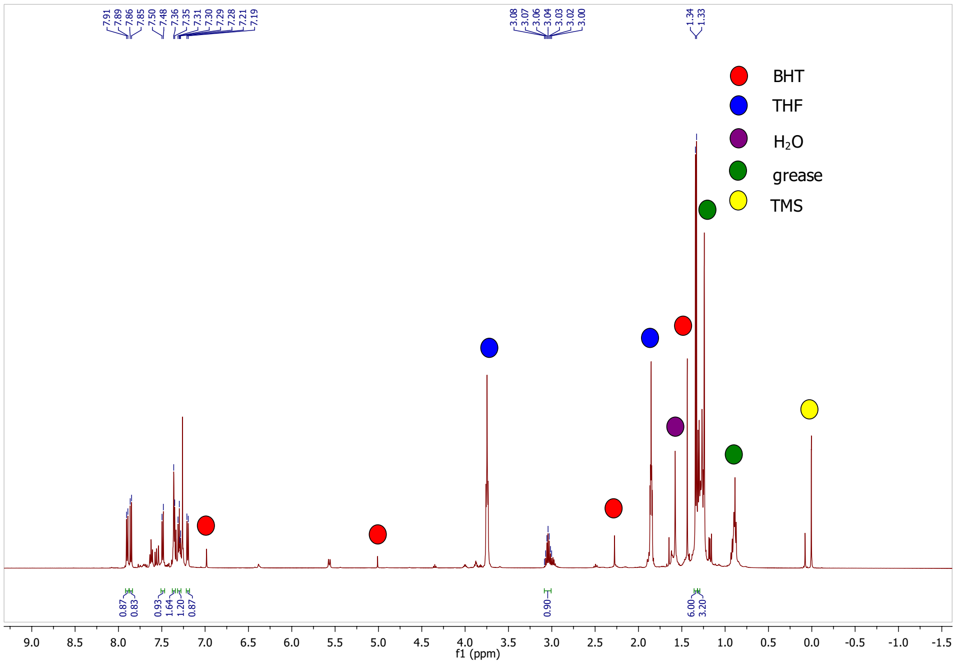

Figure S10: ^{1}H NMR spectrum of **10** in chloroform-*d*.

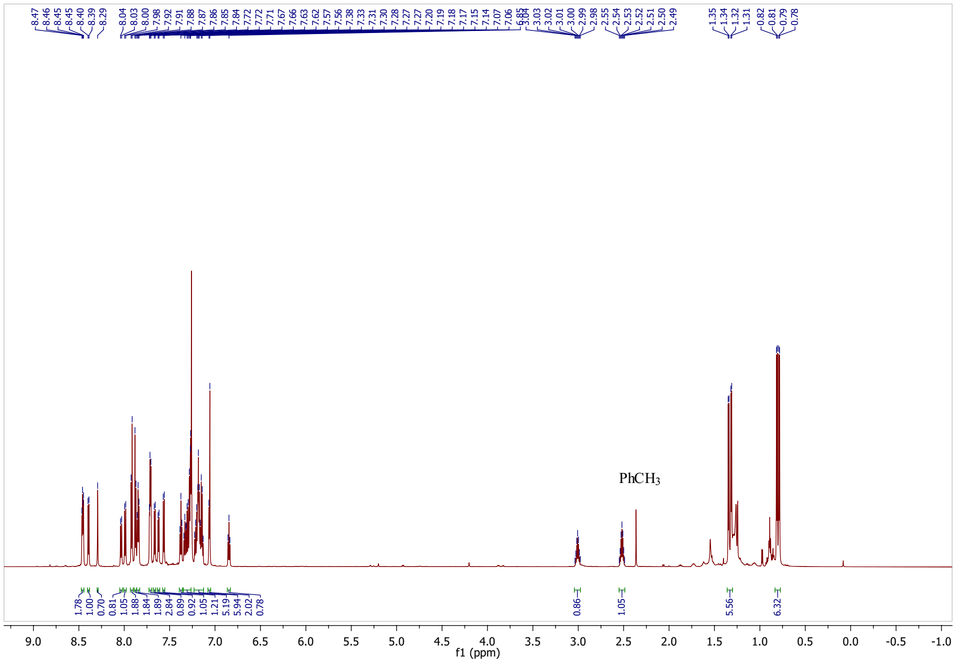

Figure S11: ^{1}H NMR spectrum of **4** in chloroform-d.

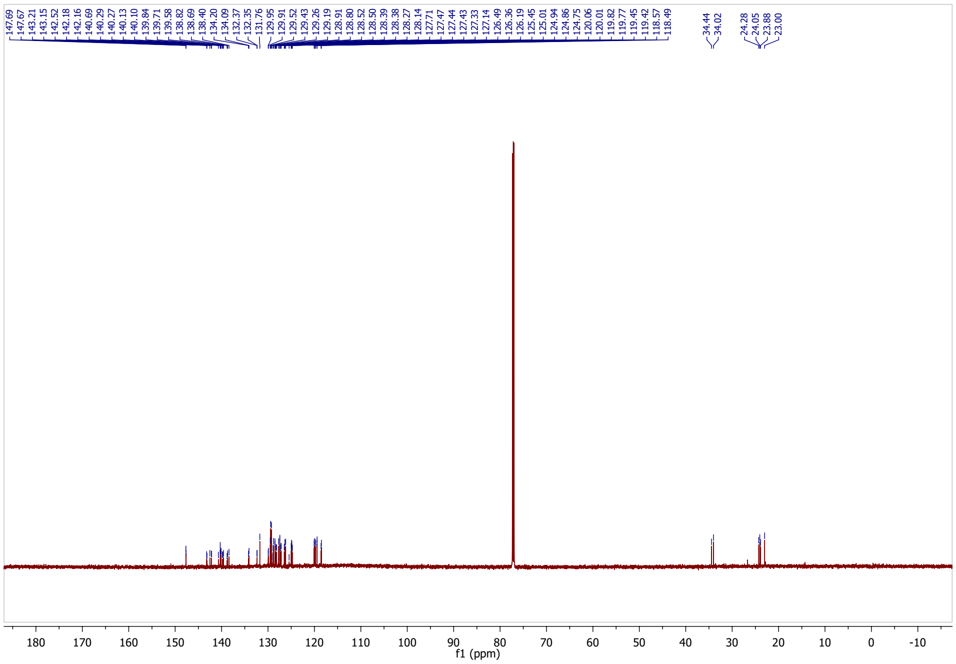

Figure S12: $^{13}C\{^1H\}$ NMR spectrum of **4** in chloroform-d.

$J = 6.9$ Hz, 2H), 6.85 (t, $J = 7.5$ Hz, 0.8H), 3.01 (sept, $J = 6.9$ Hz, 0.8H), 2.52 (sept, $J = 7.2$ Hz, 1H), 1.33 (dd, $J = 20.3, 6.9$ Hz, 5.6H), 0.80 (dd, $J = 17.2, 6.9$ Hz, 6H).

$^{13}C\{^1H\}$ NMR (176 MHz, $CDCl_3$, 20 °C, Fig. S12) δ 147.69, 147.67, 143.21, 143.15, 142.52, 142.18, 142.16, 140.69, 140.29, 140.27, 140.13, 140.10, 139.84, 139.71, 139.58, 138.82, 138.69, 138.40, 134.20, 134.09, 132.37, 132.35, 131.76, 129.95, 129.91, 129.52, 129.43, 129.26, 129.19, 128.91, 128.80, 128.52, 128.50, 128.39, 128.38, 128.27, 128.14, 127.71, 127.47, 127.44, 127.43, 127.33, 127.14, 126.49, 126.36, 126.19, 125.45, 125.01, 124.94, 124.86, 124.75, 120.06, 120.01, 119.82, 119.77, 119.45, 119.42, 118.57, 118.49, 34.44, 34.02, 24.28, 24.05, 23.88, 23.00.

HRMS (FTMS + p ESI) m/z: $[M+H]^+$ calculated for $C_{33}H_{25}$, 421.1878; found, 421.19467. UV–vis absorption (300–800 nm, THF, Fig. S19a) λ_{max} 481.

A sample of **4** in mesitylene-d_{12} was prepared for high temperature variable temperature (VT) NMR experiments. Lineshape analysis was used to determine the rate of chemical exchange at various temperatures, and the rate constants were fitted using the DNMR module of Topspin (Fig. S13a). At high temperatures during our VT NMR experiment, a few small new peaks were observed to grow in presumably due to decomposition, and these can be seen by comparison with a mesitylene-d_{12} sample before heating. Following the VT NMR experiments, we used the sample in a series of NMR experiments to assign all proton resonances, including COSY, NOESY, and various 1D selective TOCSY experiments. These spectra are shown in Figs. S14–S18. The aromatic region is especially congested, even on a 700 MHz instrument, so there remain some slight uncertainties, particularly between the positions labeled '*5*' and '*6*' in Fig. S14. Our assignments are supported by comparison of the experimental ^1H NMR spectrum with the simulated spectrum, which overlay well. Each peak labeled 'dd [t]' is a doublet of doublets that appears as a triplet.

Major Z isomer, 60%: ^1H NMR (700 MHz, mesitylene-d_{12}, 20 °C) δ 8.4813 (d, $J = 7.9$ Hz, H10), 8.3407 (d, $J = 7.7$ Hz, H8′), 8.0706 (d, $J = 8.5$ Hz, H1), 7.6355 (d, $J = 8.3$ Hz, H5/H6), 7.6337 (d, $J = 7.5$ Hz, H7), 7.6033 (d, $J = 7.7$ Hz, H4), 7.5763 (d, $J = 8.3$ Hz, H5/H6), 7.4845 (d, $H = 7.6$ Hz, H5′), 7.3668 (d, $H = 7.7$ Hz, H4′), 7.2231 (d, $J = 1.5$ Hz, H1′), 7.1796 (dd [t], $J = 7.7, 7.1$ Hz, H7′), 7.087 (dd [t], $J = 7.6, 7.1$ Hz, H6′), 7.085 (dd [t], $J = 7.5, 7.1$ Hz, H8), 6.969 (dd [t], $J = 7.9, 7.1$ Hz, H9), 6.936 (dd [t], $H = 7.7, 1.5$ Hz, H3′), 6.931 (dd [t], $J = 7.7, 7.1$ Hz, H3), 6.917 (dd [t], $J = 8.5, 7.1$ Hz, H2), 2.37 (hept, $J = 6.9$ Hz, ^{i}Pr CH), 0.82 (d, $J = 6.9$ Hz, ^{i}Pr CH_3), 0.79 (d, $J = 6.9$ Hz, ^{i}Pr CH_3).

Minor E isomer, 40%: ^1H NMR (700 MHz, mesitylene-d_{12}, 20 °C) δ 8.5333 (d, $J = 7.9$ Hz, H10), 8.3608 (d, $J = 1.5$ Hz, H1′), 8.1023 (d, $J = 8.4$ Hz, H1), 7.6498 (d, $J = 8.3$ Hz, H5/H6), 7.6028 (d, $J = 8.2$ Hz, H4), 7.5951 (d, $J = 8.3$ Hz, H5/H6), 7.4613 (d, $J = 7.5$ Hz, H7), 7.4546 (d, $H = 7.5$ Hz, H4′), 7.4013 (d, $H = 7.3$ Hz, H5′), 7.2454 (d, $J = 7.9$ Hz, H8′), 7.102 (dd [t], $J = 8.2, 7.1$ Hz, H3), 7.1004 (dd [t], $J = 7.9, 7.1$ Hz, H9), 7.0890 (dd [t], $H = 7.5, 1.5$ Hz, H3′), 7.0055 (dd [t], $J = 7.5, 7.1$ Hz, H8), 6.9952 (dd [t], $J = 7.3, 7.3$ Hz, H6′), 6.9315 (dd [t], $J = 8.4, 7.1$ Hz, H2), 6.6150 (dd [t], $J = 7.3, 7.9$ Hz, H7′), 2.821 (hept, $J = 6.9$ Hz, ^{i}Pr CH), 1.302 (d, $J = 6.9$ Hz, ^{i}Pr CH_3), 1.239 (d, $J = 6.9$ Hz, ^{i}Pr CH_3).

EXSY spectra were acquired using a Bruker Avance III 600 or 700 MHz spectrometer equipped with a Bruker BVT3000 variable temperature control unit. Temperatures were calibrated using a DMSO/ethylene glycol external standard. Experiments were run using selective excitations for a 1D NOESY pulse sequence where dipolar couplings due to chemical exchange (positive) or nuclear-Overhauser-effect (negative) are observed.[8] Integration of ^1H EXSY spectra was performed using MestReNova 14.3.0 and fitting of rate constants was done using Wolfram Mathematica 13.1. Rate constants of E/Z isomerization of **4** at temperatures of 50–70 °C were obtained as reported by Perrin[9] using mixing times of $\tau_m = 0.0001-3$ s (Fig. S13b). Barriers were then fit to the Eyring equation,

$$k = \frac{\kappa k_B T}{h} \exp\left(-\frac{\Delta H^\ddagger - T\Delta S^\ddagger}{RT}\right). \tag{S1}$$

where the transmission coefficient κ has been assumed to be unity.

S1.2 Reduced Radical Anionic Bifluorenylidenes

S1.2.1 Potassium Bifluorenylidene $[K(THF)_4][1]$

Inside of a nitrogen-filled glovebox, metallic potassium (11.9 mg, 0.304 mmol) was smeared along the walls of a 20 mL scintillation vial and combined with a magnetic stir bar and THF (4 mL). To this mixture, a

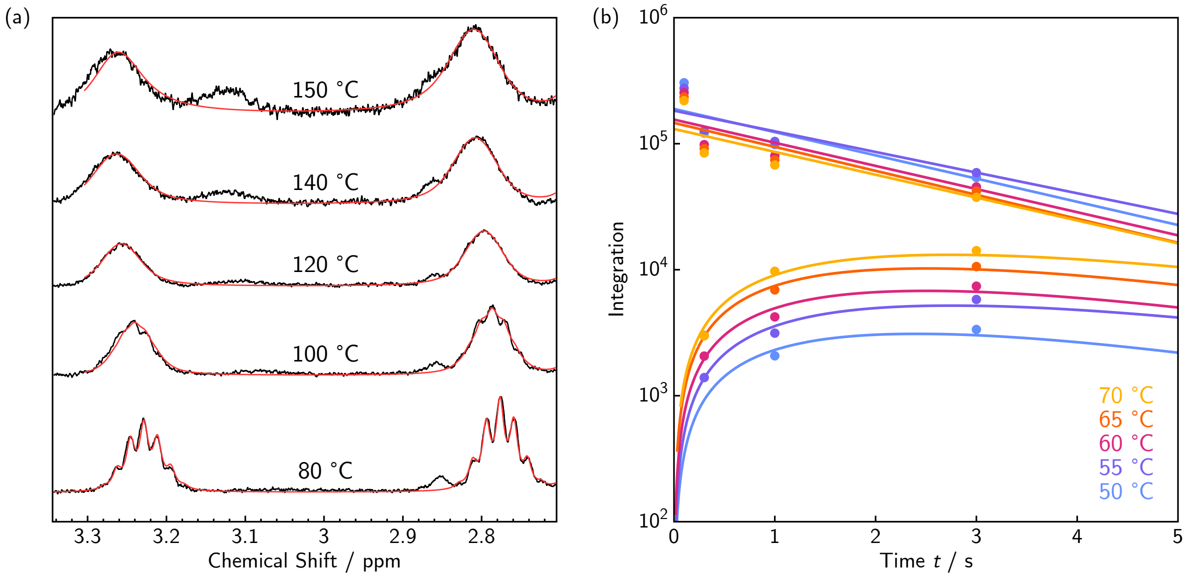

Figure S13: (a) Higher temperatures showed a broadening due to chemical exchange and this was modeled using TopSpin's DNMR module. (b) Selective 1D EXSY experiments allowed monitoring of $E \rightleftharpoons Z$ isomerization even when slow enough that its effects were not observed on the lineshape.

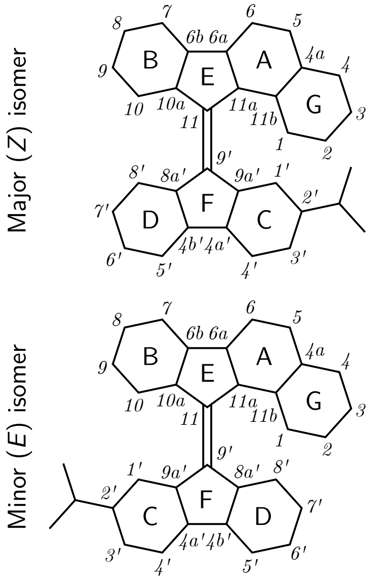

Site	Major (Z) δ	J	Minor (E) δ	J
1	8.0706	8.5	8.1023	8.4
2	6.917	8.5, 7.1	6.9315	8.4, 7.1
3	6.931	7.7, 7.1	7.102	8.2, 7.1
4	7.6033	7.7	7.6028	8.2
5/6	7.6355	8.3	7.6498	8.3
6/5	7.5763	8.3	7.5951	8.3
7	7.6337	7.5	7.4613	7.5
8	7.085	7.5, 7.1	7.0055	7.5, 7.1
9	6.969	7.9, 7.1	7.1004	7.9, 7.1
10	8.4813	7.9	8.5333	7.9
1′	7.2231	1.5	8.3608	1.5
3′	6.936	7.7, 1.5	7.0890	7.5, 1.5
4′	7.3668	7.7	7.4546	7.5
5′	7.4845	7.6	7.4013	7.3
6′	7.087	7.6, 7.1	6.9952	7.3, 7.3
7′	7.1796	7.7, 7.1	6.6150	7.9, 7.3
8′	8.3407	7.7	7.2454	7.9
^{i}Pr CH	2.37	6.9	2.821	6.9
^{i}Pr CH_3	0.82	6.9	1.302	6.9
^{i}Pr CH_3	0.79	6.9	1.239	6.9

Figure S14: Assignments of ^{1}H resonances in the NMR spectrum of **4** in mesitylene-d_{12}.

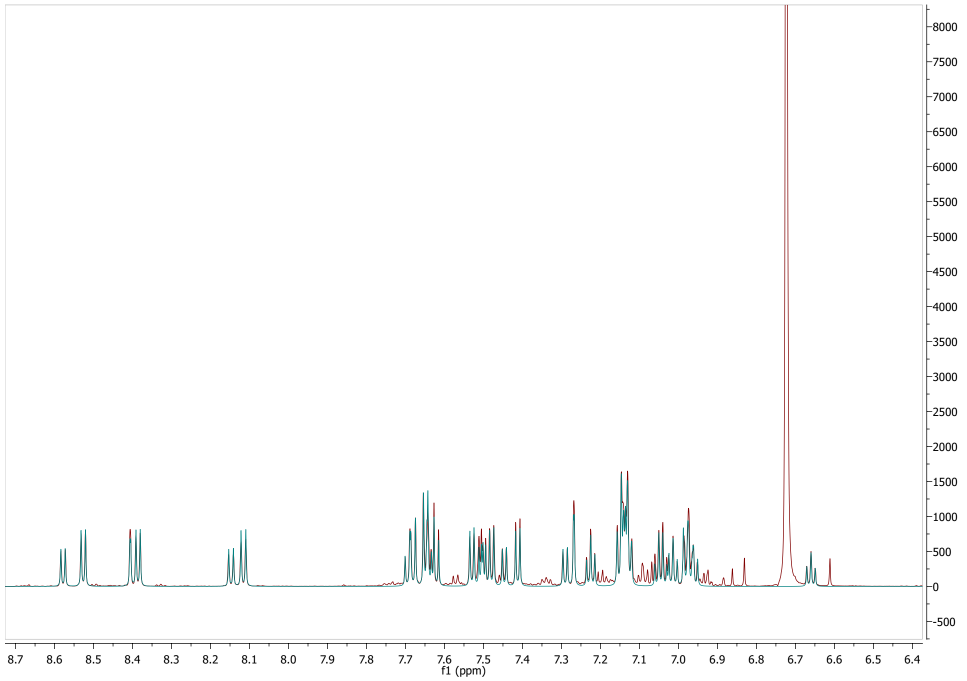

Figure S15: Overlay of experimental ^{1}H NMR spectrum of **4** (700 MHz, 20 °C) with a simulation using the chemical shifts and J coupling values shown in Fig. S14. The smaller unaccounted-for peaks are small impurities that developed while heating to 150 °C for VT DNMR analysis, along with the mesitylene-d_{12} residual proton peak and its ^{13}C satellites.

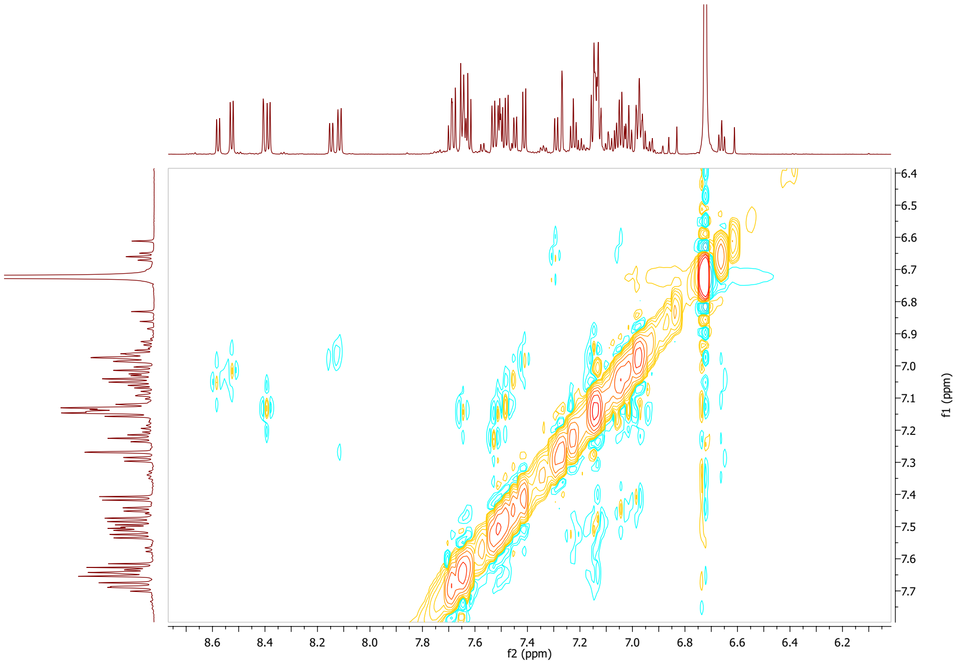

Figure S16: ^{1}H NOESY spectrum of **4** in mesitylene-d_{12}.

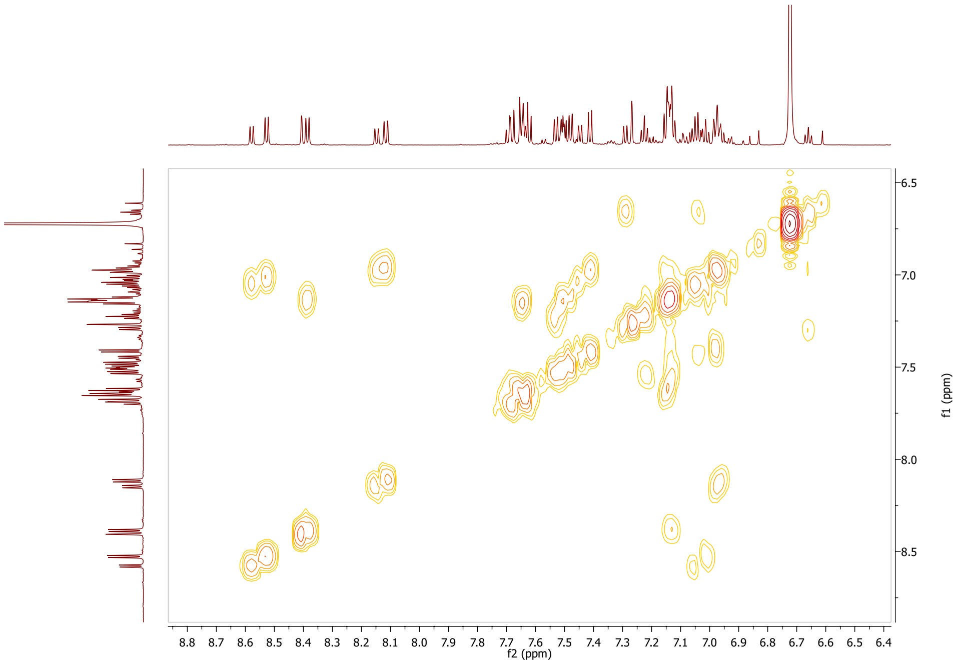

Figure S17: ^{1}H COSY spectrum of **4** in mesitylene-d_{12}.

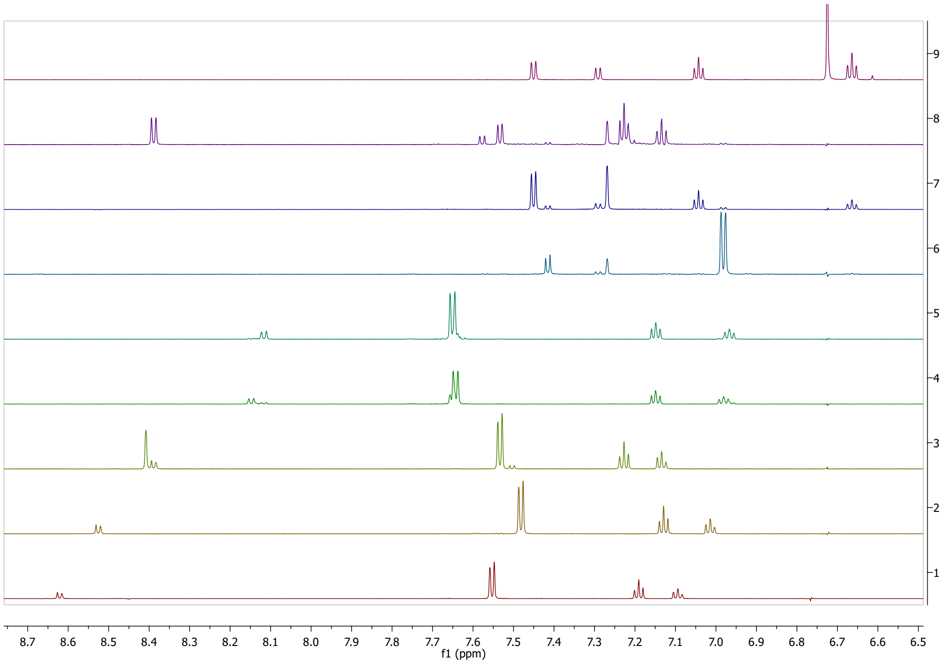

Figure S18: All selective one-dimensional ^{1}H TOCSY NMR experiments run on **4** in mesitylene-d_{12} that were used in making assignments.

solution of **1** (100 mg, 0.304 mmol) in THF (2 mL) was added. This caused a color change originating from the surface of the potassium from the bright orange of **1** to a darker green. The mixture was strongly stirred at room temperature for 4 h. The solution turned dark green. The mixture was decanted from any remaining potassium still present and concentrated under reduced pressure to a volume of approximately 4 mL. Vapor diffusion crystallization cells made by diffusing hexane into this solution at room temperature yielded green needles suitable for preliminary X-ray diffraction studies (59 mg, 53%). This compound was found to be NMR-silent in THF-d_8 solution at room temperature; instead, it showed an intricate EPR spectrum (Section S3). UV–vis–NIR absorption (300–1600 nm, THF, Fig. S19c) λ / nm [ϵ / M^{-1} cm^{-1}] 355 [8 440 sh], 367 [11 500], 373 [11 900], 398 [13 400], 451 [8 980], 835 [12 700].

S1.2.2 Potassium Reduction of 2 and 3

Both **2** and **3** were available in far smaller quantities than **1**, which inhibited our ability to develop a preparative route. Reduced samples were prepared once on small scales with limited quantities of crystalline material isolated for analysis. The following was performed under an inert atmosphere within a N_2-filled glovebox.

Reduction of 2. Metallic potassium (7.3 mg, 0.187 mmol) was smeared along the walls of a 20 mL scintillation vial, then a diethyl ether solution (5 mL) of **2** (14.5 mg, 0.0383 mmol) was added. Stirring overnight (16 h) caused a color change from bright red to burgundy. The solution was decanted away from excess potassium metal, then a second equivalent of **2** (14.5 mg, 0.0383 mmol) in THF (5 mL) was added, causing an immediate color change to dark purple. After stirring 1 h, the solvent was removed under reduced pressure and the dark residue was recrystallized by vapor diffusion of hexane into a THF solution at room temperature. After 2 days, a small quantity of long brown-purple needles were collected and separated for analysis by single-crystal X-ray diffraction, UV–vis–NIR absorption/MCD spectroscopy, and EPR spectroscopy. The sample for EPR spectroscopy was prepared as a 2-methyltetrahydrofuran solution and flame-sealed into a 4 mm OD tube.

Reduction of 3. Metallic potassium (4.9 mg, 0.125 mmol) was smeared along the walls of a 20 mL scintillation vial, then a THF solution (6 mL) of **3** (45.1 mg, 0.105 mmol) was added. Stirring overnight (18 h) caused consumption of the potassium metal, but the solution remained dark purple throughout without a pronounced color change. The solution was decanted, dried, and the solids were recrystallized by vapor diffusion of hexane into a THF solution at room temperature. A small quantity of dark blue-purple crystals were collected and separated for analysis by X-ray crystallography (attempted), UV–vis–NIR absorption/MCD spectroscopy, and EPR spectroscopy. While crystals suitable for X-ray diffraction were not obtained, the other characterization techniques were successful. The sample for EPR spectroscopy was prepared as a 2-methyltetrahydrofuran solution and flame-sealed into a 4 mm OD tube.

S2 UV–Vis–NIR Absorption and MCD Spectroscopy

S2.1 Data Acquisition and Processing

Absorption and magnetic circular dichroism (MCD) spectroscopies were performed using a JASCO J-1700 spectropolarimeter with equipped with a S-20 photocathode-equipped photomultiplier tube (PMT) detector ($\lambda = 163-950$ nm, JASCO Model PM-539), a S-1 photocathode-equipped PMT detector ($\lambda = 400-1250$ nm, JASCO Model EXPM-531), or an InGaAs detector ($\lambda = 800-1600$ nm JASCO Model EXIG-542 or $\lambda = 1600-2500$ nm EXIG-543). Room-temperature MCD data were acquired using a JASCO MCD-581 electromagnet ($|B| \leq 1.5$ T). Samples held within the electromagnet setup were generally found to be $18-20$ °C. Cuvettes were purchased from Spectrocell (1 or 10 or 40 mm "NIR" quartz cells, 220–3500 nm range), and some were adapted to have a Young's valve to allow for analysis of air-free samples.

Data were generally acquired at a 1 nm data pitch, a 500 nm/min scanning speed, a 0.25 or 0.5 s detector integration time, and a 2 nm bandwidth. For peaks that appeared especially narrow, these parameters were varied to ensure that no peak distortion was apparent. When multiple detectors were necessary to fully characterize the room temperature MCD spectra of a compound, the same sample was used across all regions. Solvent blanks were collected under identical conditions to the absorption/MCD spectra of the samples, and were subtracted from the final data.

MCD spectra were acquired at a series of field strengths (e.g. 0, ±1, ±1.5 T). These experimental conditions are in the linear limit ($\mu_B B/k_B T \ll 1$), giving MCD intensity strictly proportional to the applied field. Under these linear conditions, the field-independent and field-dependent components of the measured ellipticity data were separated using a Moore–Penrose pseudoinverse. For example, collection of five spectra over the 300–800 nm region at $B = 0, \pm1, \pm1.5$ T field strengths allows extraction of the baseline (field-independent $I^{(0)}$) and MCD (field-dependent $I^{(1)}$) components through

$$\begin{pmatrix} 1 & 1.5 \\ 1 & 1 \\ 1 & 0 \\ 1 & -1 \\ 1 & -1.5 \end{pmatrix}^{\ominus} \begin{pmatrix} I_{300\text{ nm}}^{+1.5\text{ T}} & I_{301\text{ nm}}^{+1.5\text{ T}} & I_{302\text{ nm}}^{+1.5\text{ T}} & \cdots & I_{800\text{ nm}}^{+1.5\text{ T}} \\ I_{300\text{ nm}}^{+1.0\text{ T}} & I_{301\text{ nm}}^{+1.0\text{ T}} & I_{302\text{ nm}}^{+1.0\text{ T}} & \cdots & I_{800\text{ nm}}^{+1.0\text{ T}} \\ I_{300\text{ nm}}^{0.0\text{ T}} & I_{301\text{ nm}}^{0.0\text{ T}} & I_{302\text{ nm}}^{0.0\text{ T}} & \cdots & I_{800\text{ nm}}^{0.0\text{ T}} \\ I_{300\text{ nm}}^{-1.0\text{ T}} & I_{301\text{ nm}}^{-1.0\text{ T}} & I_{302\text{ nm}}^{-1.0\text{ T}} & \cdots & I_{800\text{ nm}}^{-1.0\text{ T}} \\ I_{300\text{ nm}}^{-1.5\text{ T}} & I_{301\text{ nm}}^{-1.5\text{ T}} & I_{302\text{ nm}}^{-1.5\text{ T}} & \cdots & I_{800\text{ nm}}^{-1.5\text{ T}} \end{pmatrix} = \begin{pmatrix} I_{300\text{ nm}}^{(0)} & I_{301\text{ nm}}^{(0)} & I_{302\text{ nm}}^{(0)} & \cdots & I_{800\text{ nm}}^{(0)} \\ I_{300\text{ nm}}^{(1)} & I_{301\text{ nm}}^{(1)} & I_{302\text{ nm}}^{(1)} & \cdots & I_{800\text{ nm}}^{(1)} \end{pmatrix},$$

where "$\ominus$" indicates the pseudoinverse. The first column of ones in the leftmost matrix indicates that the baseline is constant among the data sets, and the second column of the leftmost matrix contains the strengths of the applied fields. The output gives the field-independent ellipticity in its first row (i.e. the baseline), and the field-dependent ellipticity in its second row (i.e. MCD expressed in millidegrees per tesla). The field-dependent ellipticity $[\theta]$ was converted to $\Delta\epsilon$ MCD intensity by $\Delta\epsilon = [\theta\,(\text{mdeg})]/(32982cl)$ using concentration c and path length l.

S2.2 Absorption and MCD Spectra of Diamagnetic Species

The absorption spectrum of **1** was obtained over a 250–800 nm wavelength range (Fig. S19a). Spectra were acquired in a variety of solvents (THF, 3:2 ethanol/methanol, chloroform) and there were no appreciable shifts in maxima of the features. Adherence of the absorption spectrum to Beer's law over the 300–600 nm wavelength window was checked over a concentration range of 10 µM to 1.7 mM. A series of cuvette path lengths was used to accomplish this (1, 10, 40 mm). All spectra for **1**–**3** in the main manuscript were collected in THF.

The MCD spectra of the neutral species were quite weak (Fig. S19c). Spectra were collected with 16 accumulations each, and a large number of field strengths (±1.5, ±1.4, ±1.3, ±1.2, ±1.1, ±1.0, ±0.7, 0 T) were acquired to try to separate the field-dependent and field-independent components. The data were also acquired at a variety of concentrations so that the intensities around the wings of the strong $\pi \to \pi^*$ transitions could be confidently acquired. Due to the weak intensity of the MCD signals, care was taken to ensure we were truly recording circular dichroism rather than linear dichroism/linear birefringence (LDLB) artifacts. The desired CD intensity has a pathlength dependence of ℓ and the undesired LDLB intensity has a pathlength dependence of ℓ^2,[10] so comparisons of our data sets acquired with two different path lengths (1 vs 10 mm) and multiple concentrations gave us confidence that our signals were not artifactual.

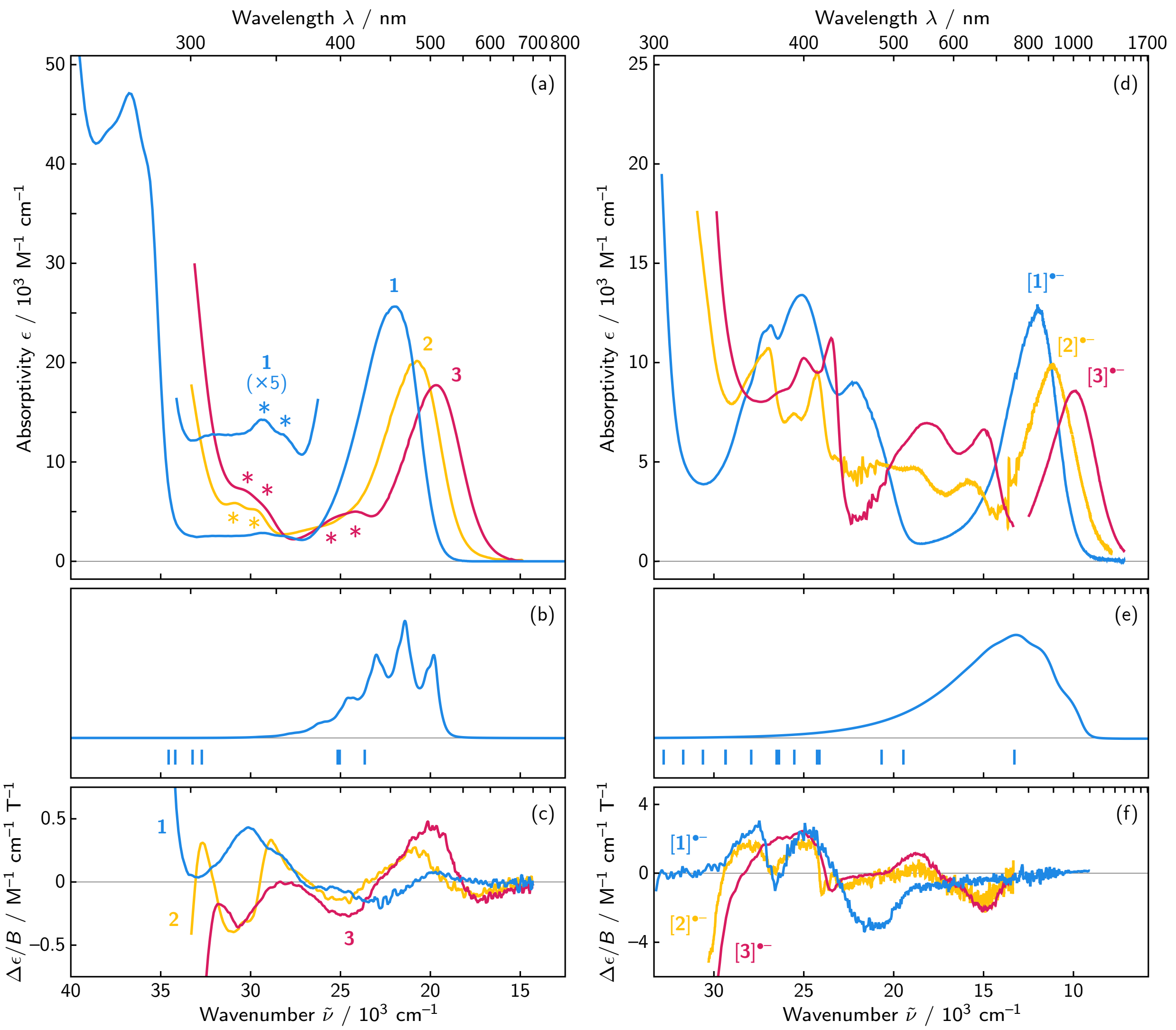

Figure S19: Absorption spectra of the (a) neutral and (d) anionic BFs in THF solution at room temperature. The lowest energy transitions are strong $\pi \to \pi^*$ features. Neutral compounds show a number of smaller features in the 300–450 nm region, and these seem to appear in pairs (marked with asterisks). TD-DFT calculations on (b) **1** and (e) [**1**]$^{\bullet-}$ predict many transitions, shown as sticks (Section S5.1.2). Excited state dynamics calculations on the $\pi \to \pi^*$ transitions predict vibrational progression that coarsely reproduce the experimental observations. MCD spectra show a large number of transitions throughout the UV–vis–NIR for the (c) neutral and (f) anionic species.

S2.3 Absorption and MCD Spectra of Paramagnetic Reduced Species

Absorption spectra were acquired in an airfree cuvette for all anions in THF solution (Fig. S19d). Adherence of the broad NIR absorbance present in samples of K[**1**] to Beer's law over the 800–1200 nm wavelength window was confirmed over a concentration range of 77 µM to 211 µM. The MCD spectra of the anionic species were considerably more intense than the neutral precursors (Fig. S19f). Spectra were collected with 16 accumulations each, at various field strengths (±1.5, ±1.4, ±1.3, 0 T). These data were used to try to separate the field-dependent and field-independent components. The sensitivity of these samples to traces of air prevented longer data accumulations and more fields.

A series of [**1**]$^{\bullet-}$ anions were prepared using a variety of reductants (Na, K, Mg) resulting in identical

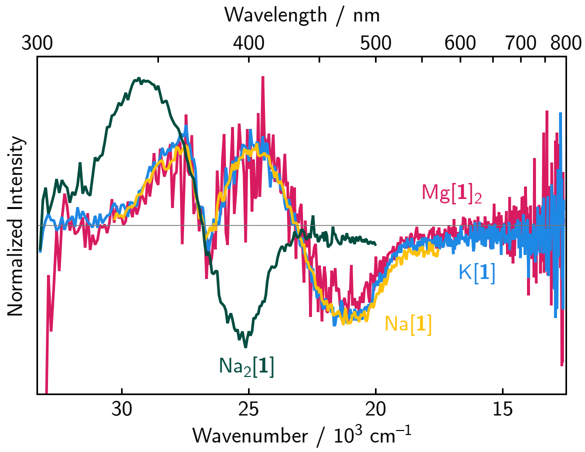

Figure S20: Reduction of **1** using sodium, potassium, and magnesium metal all produce a monoanionic species with identical MCD spectra in THF at room temperature. The lack of change in the spectra upon changing the cation suggests that the THF solvent molecules preferentially solvate the cation to yield solvent-separated ionic pairs. The only deviation was found when excess sodium was used in diethyl ether, conditions similar to those known to produce the BF dianion.[11–13] This spectrum is labeled 'Na_2[**1**]' in green.

absorbance and MCD spectra (Fig. S20). The only difference was found when using excess sodium as a reductant in diethyl ether; similar conditions are known to doubly reduce BF to its dianionic species.[11–13] The lack of a counter-ion dependence of these spectra strengthen the interpretation that in solution these ions are fully dissociated in contrast to their crystalline structure, which showed haptic potassium–bifluorenylidene interactions.

S3 Electron Paramagnetic Resonance

S3.1 Continuous Wave X-Band EPR Experiments

Unless otherwise noted, all samples were ∼0.1 mM in 2-methyltetrahydrofuran. Samples were contained in 4 mm quartz EPR tubes with a J Young tap, or flame sealed within a quartz tube. The g values were externally calibrated using DPPH (2.0036 ± 0.0001).[14] Room temperature CW-EPR experiments were performed with a X-Band Bruker EMX CW EPR spectrometer. Data collection was initiated by conducting a power sweep, and a non-saturating power setting was selected to avoid any lineshape distortions. The sharpness of the spectrum of $[\mathbf{1}]^{\bullet-}$ prompted us to take particular care to ensure the acquisition parameters were not distorting the signal.[14] All measurements were done with a single scan because even slight drifts in the microwave frequency caused appreciable broadening upon signal averaging. Spectra were acquired using 22 dB attention (0.6310 mW power), a sweep width of 30 G, and a sweep time of 360 s, a modulation frequency of 10 kHz, a modulation amplitude of 0.03 G, a conversion time of 36.00 ms, a time constant of 10.24 ms, and 10 000 total data points. Variable temperature (VT) CW-EPR experiments were performed with a Bruker ElexSys E680 CW/FT X-band spectrometer. The spectra were collected with a Bruker ER4118-MD5 resonator, Oxford ITC503 temperature controller and an Oxford CF935 dynamic continuous-flow cryostat. Each sample was allowed to equilibrate at each temperature for 1 h. The parameters for the VT CW-EPR experiments were: center field = 3470 G, sweep width = 40 G, microwave frequency = ∼9.73 GHz, modulation amplitude = 0.1 G, modulation frequency = 100 kHz, conversion time = 20.48 ms, number of scans = 1, number of points = 1024. The rigid limit 80 K CW-EPR experiments were conducted on a Bruker ElexSys E580 CW/FT X-band spectrometer. CW-EPR parameters were: center field = 3496 G, sweep width = 100 G, microwave frequency = ∼9.81 GHz, modulation amplitude = 1 G, modulation frequency = 100 kHz, conversion time = 20.48 ms, number of points = 1024. The spectra for compounds **1**, **2**, and **3** were recorded for 256, 64, and 128 scans respectively.

Table S1: EasySpin Fits

Parameter	$[\mathbf{1}]^{\bullet-}$	Single Gaussian		DFT Informed	
		$[\mathbf{2}]^{\bullet-}$	$[\mathbf{3}]^{\bullet-}$	$[\mathbf{2}]^{\bullet-}$	$[\mathbf{3}]^{\bullet-}$
`S`	0.5	0.5	0.5	0.5	0.5
`g`	2.0028	2.0025	2.0025	2.0025	2.0025
`Nucs`	`'1H,1H,1H,1H'`				
`n`	`[4 4 4 4]`				
`A`	5.42169<br>4.33840<br>1.47697<br>0.877817			×*0.976722*	×*0.833547*
`lw`	0.0159245	0.644624	0.629696	0.1	0.04

Spectra were simulated using the `garlic` function (isotropic/fast motion) in the EasySpin software.[15] EPR spectrum fitting is a nonlinear regression problem and requires well-informed initial guesses for each variable describing the system. Initial hyperfine values for $[\mathbf{1}]^{\bullet-}$ were taken from an earlier literature report.[16] While the literature report was conducted under somewhat different conditions, its values should be similar to ours. The `esfit` subroutine was used to perform the fitting using a quadratic baseline shift and least squares autoscaling. Initial g values and linewidth values were estimated by holding any A values constant at their literature values and then floating the `g` and `lw` parameters against the integrated spectrum. A genetic algorithm was then used to float all values simultaneously against the data 'as-is' (i.e. no longer integrated). The best values obtained from the genetic search were further refined using the Nelder–Mead simplex algorithm to obtain the final values listed in Table S1. Comparisons between the experimental and simulated spectra can be seen in Fig. S21a.

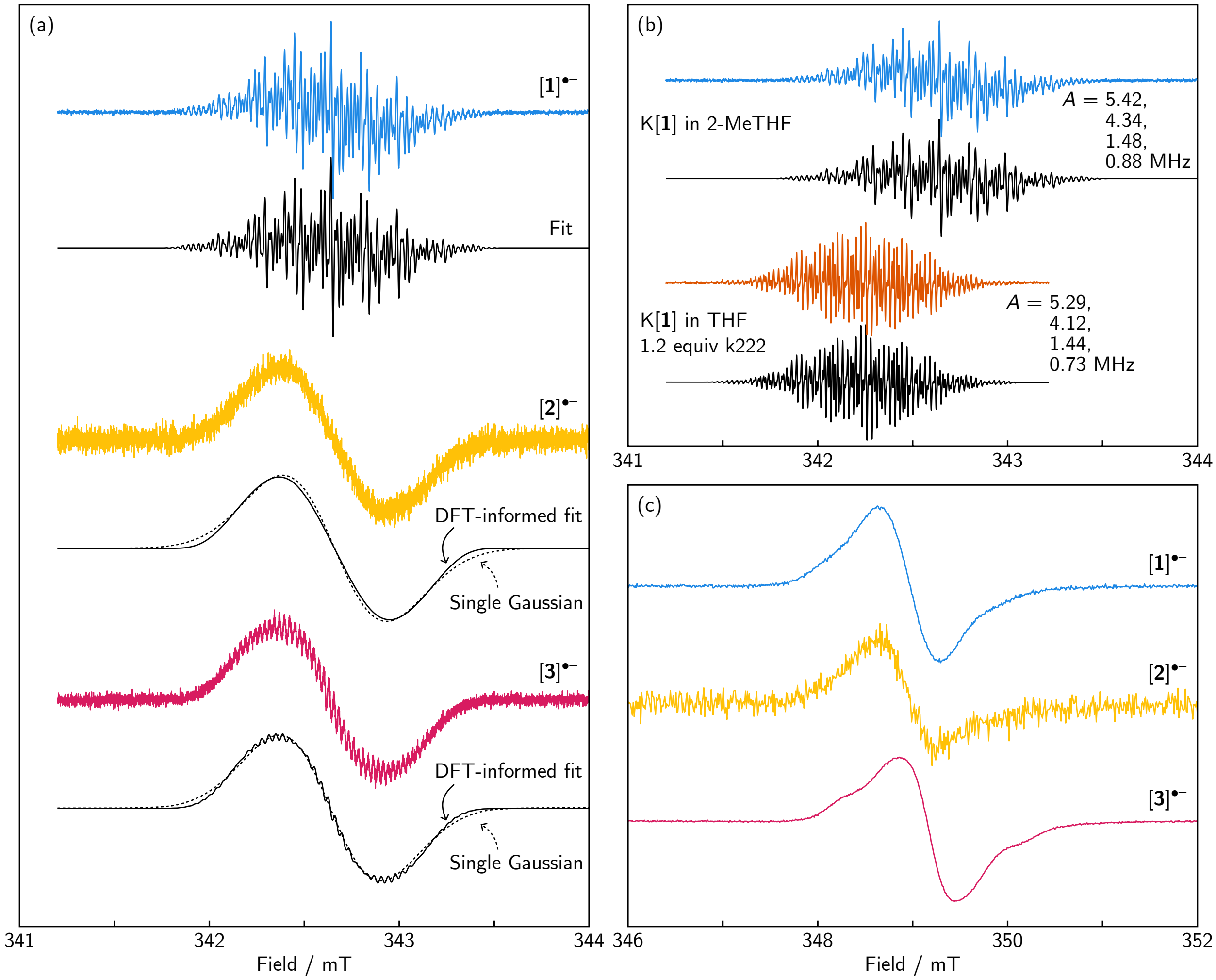

Figure S21: (a) Experimental and simulated/fitted EPR spectra for reduced BF systems at room temperature in 2-methyltetrahydrofuran (2-MeTHF). (b) Comparison of the X-band CW EPR spectra of $K(THF)_4$ in 2-methyltetrahydrofuran solution, and in a THF solution spiked with 1.2 equiv Kryptofix-222 to sequester the potassium cation. While the spectra appear different in shape, fitting using EasySpin shows only slight changes in hyperfine coupling constants. (c) EPR spectra of frozen 2-MeTHF solutions at 80 K.

The broader features for [**2**]$^{\bullet-}$ and [**3**]$^{\bullet-}$ were fitted in two ways. The simplest method fit a single Gaussian feature to the spectrum, ignoring any subtleties in the lineshape or any apparent hyperfine features. A second method was used that was informed by the hyperfine constants from DFT calculations (see Section S5.1.3). The data were fitted by scaling the DFT-calculated hyperfine interactions to best reproduce the overall lineshape. This involved floating the g value and the scaling constant. The underlying linewidth could not be determined for [**3**]$^{\bullet-}$: the experiment data showed small hyperfine features with peak-to-trough distances of ∼0.011 mT but the mismatch between the experimental hyperfine and DFT-scaled hyperfine seemed to always cause the fitting subroutine to smooth out any hyperfine features with far larger linewidths (>0.05 mT). Table S1 lists the fitted g value along with the best scaling constant (the italicized number in the `A` row). Simulation of [**2**]$^{\bullet-}$ through this method was largely insensitive to `lw` because it was strongly correlated with the scaling constant; thus, a value of 0.1 was arbitrarily chosen. The linewidth used for [**3**]$^{\bullet-}$ was somewhat arbitrarily set to 0.04 mT since the fit appeared to be stable and showed some residual hyperfine features. We do not claim the linewidths for [**2**]$^{\bullet-}$ and [**3**]$^{\bullet-}$ in Table S1 are meaningful; further experiments would be necessary to accurately determine experimental hyperfine A_{iso} values.

The spectrum of [**1**]$^{\bullet-}$ in 2-MeTHF was sharp and showed D_2 point group symmetry of the molecule,

indicating that the potassium ion does not remain bound to the central C=C bond of the molecule as seen in the crystal structure of $K(THF)_4$[**1**]. This could either indicate dynamic movement of the potassium ion around the BF anion faster than the EPR timescale, or it could indicate that the solvent abstracts the cation to form a $[K(THF)_x)(2\text{-MeTHF})_y]$[**1**] ion pair. The insensitivity of the MCD spectra of $[\mathbf{1}]^{\bullet-}$ to cation identity (Section S2.3) suggests the cation is likely solvated and separated from the BF anion. To further explore the possible cation–anion interactions, we prepared a sample of $K(THF)_4$[**1**] in THF solution with 1.2 equiv Kryptofix-222 to sequester the potassium cation (Fig. S21b). While the spectrum in 2-MeTHF and the spectrum in THF with Kryptofix-222 looked visually distinct at first glance, fitting the EPR spectrum showed very similar hyperfine interactions: $A = 5.2883, 4.1239, 1.4412, 0.7332$ MHz, all within 0.22 MHz of the 2-MeTHF solution. Clearly, the overlapping $5^4 = 625$-line pattern caused small changes in A values to yield larger changes in the appearance of the spectrum. The hyperfine constants for K[**1**] in THF have been reported as $A = 5.41, 4.23, 1.5, 0.76$ MHz (1.93, 1.51, 0.54, 0.27 G) by Chippendale et al.,[17] as $A = 5.32, 3.36, 1.5, 0.76$ MHz (1.90, 1.20, 0.53, 0.27 G) by Lewis and Singer.[18] We suspect these small variations in hyperfine coupling constants can be partially ascribed to slight drifting in resonant frequency during the course of the acquisition. In total, we think it is reasonable to conclude that the potassium ion is not bound to the BF anion in THF or 2-MeTHF solution due to our combined MCD and EPR results.

S3.2 Simulation of Chemical Exchange in $[E/Z\text{-}\mathbf{3}]^{\bullet-}$

Variable temperature (VT) NMR and EPR experiments alike can provide information on dynamic processes on their respective timescales. As the timescale of the $E \rightleftharpoons Z$ isomerization approaches that of the EPR transitions, there should be a dip in coherence and a coalescence of features. We performed VT EPR measurements over a temperature range of 183–303 K to watch for any changes in lineshape. None were seen, indicating the temperatures used were either appreciably higher or lower than the coalescence temperature of the process. Computational results (Table S5) suggested we would likely need to be able to warm the sample above 303 K to start to see any coalescence behavior, but 303 K was the maximum temperature for which our resonator was rated.

EasySpin was used to explore the likely temperature ranges around which coalescence might lead to changes in lineshape. Simulations were performed using a linewidth of 0.016 mT because this was the linewidth for the sharp $[\mathbf{1}]^{\bullet-}$ anion, and because the residual hyperfine coupling seen in the CW spectrum of $[\mathbf{3}]^{\bullet-}$ had peak-to-trough differences of ∼0.011 mT. Simulations were performed using isotropic hyperfine constants scaled by ×0.833547 from the DFT calculations (see Table S1 and Table S18). Rates were calculated from the Eyring equation at several values of $\Delta H^{\ddagger}$, assuming $\Delta S^{\ddagger} = -2.3$ cal/mol·K which is the value from DFT thermochemistry calculations (Section S5.1.1). The simulated spectra can be seen in Fig. S22 alongside experimental data. All experimental data were adjusted to the microwave frequency of the 303 K data set. There are clear differences in the pattern of features in the experimental and simulated spectra, so this treatment is clearly insufficient to provide definitive insight into chemical exchange dynamics. Nonetheless, if we focus only on the temperature ranges that give rise to changes in lineshape, we can coarsely estimate the barrier heights. Because we did not see any noticeable change in lineshape up to 303 K, it seems likely that we can place a lower bound of $\Delta H^{\ddagger} \gtrsim 9$ kcal/mol.

S3.3 Pulsed EPR Experiments

Pulsed inversion recovery and Hahn-echo decay experiments were performed on the Bruker ElexSys E680 CW/FT X-band spectrometer, with a 1 kW TWT amplifier. Samples were allowed to equilibrate at each temperature for 1 h. The inversion recovery pulse sequence used is as follows: $\pi - T - \pi/2 - t - \pi - t -$ echo. The parameters are as follows: $\pi/2 = 12$ ns, $\pi = 24$ ns, and $t = 400$ ns. The initial value of T was 1000 ns and T was incremented in steps of 500 ns for 4096 points. The time domain signals were fit to a stretched exponential in the form of $I(T) = A * \left[1 - e^{-(T/T_1)^{\alpha}}\right] + C$ to determine T_1. $I(T)$ is the integrated echo area at each point T. α is the stretching component of the exponential. The Hahn-echo decay pulse sequence used is as follows: $\pi/2 - T - \pi - T -$ echo. The parameters are as follows: $\pi/2 = 12$ ns, and $\pi = 24$ ns. The initial value of T was 100 ns and this time period was incremented in steps of 4 ns for 2048 points. The resulting time domain signals were fit to a stretched exponential decay of the form $I(T) = A * e^{-(T/T_m)^{\beta}}$ to determine T_m. For this equation, β is the exponential stretching factor.

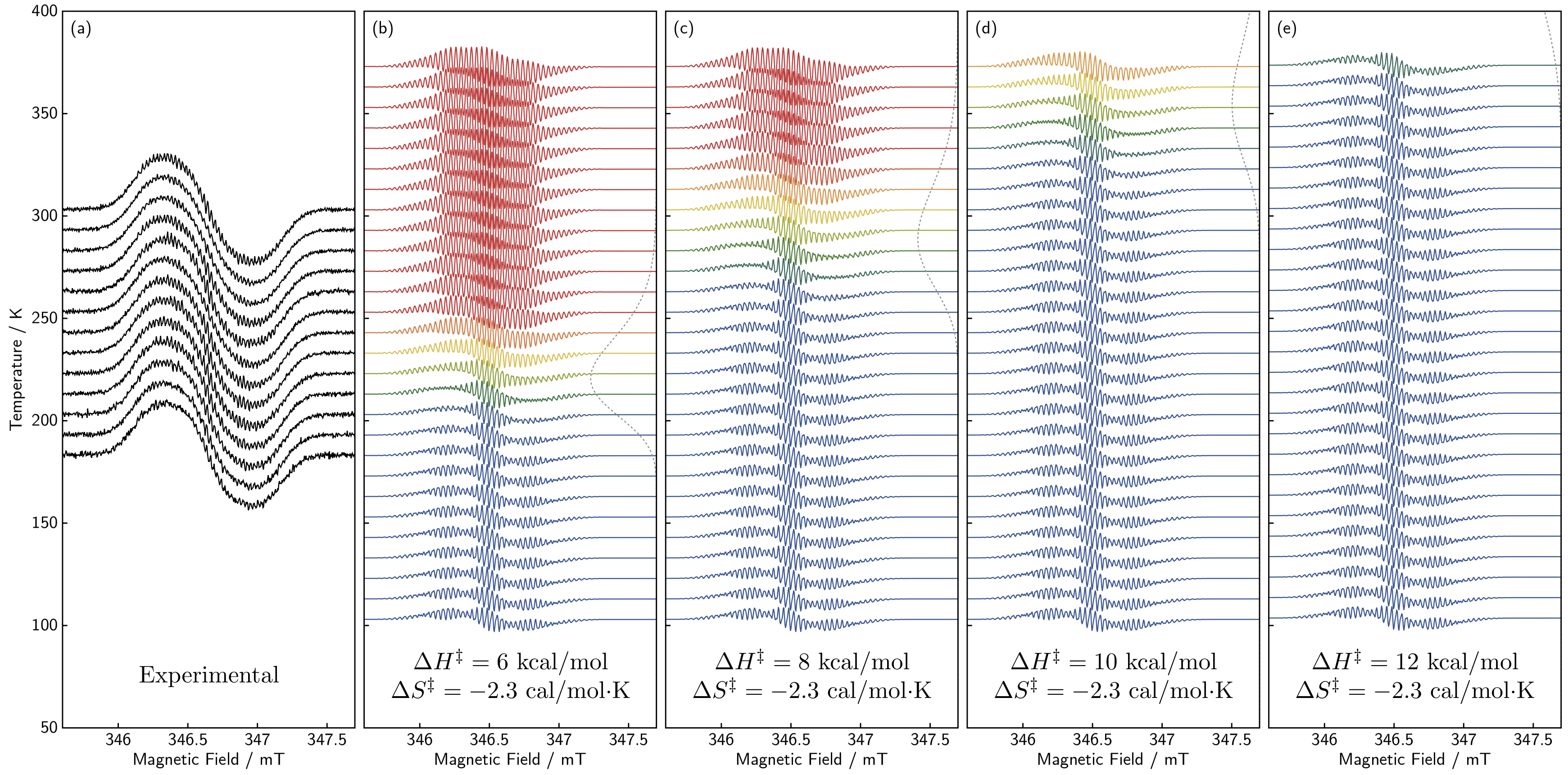

Figure S22: (a) Variable temperature CW EPR spectra at X-band did not show an appreciable change in lineshape over a 183–303 K range. The impact of a $E \rightleftharpoons Z$ isomerization chemical exchange process has been simulated using scaled DFT hyperfine constants. The entropy of the process was chosen to be $\Delta S^\ddagger = -2.3$ cal/mol·K to match DFT, and the enthalpy was varied: (b) 6 kcal/mol, (c) 8 kcal/mol, (d) 10 kcal/mol, (e) 12 kcal/mol. This suggests the barrier to $E \rightleftharpoons Z$ isomerization is likely at least 10 kcal/mol for $[\mathbf{3}]^{\bullet -}$.

S3.4 Discussion of Spin–Lattice Relaxation in Solution

S3.4.1 Relaxation Processes and Rotational Correlation Times

Through several simplifying assumptions,[19] it is possible to express spin–lattice relaxation as a sum of rates from individual contributing processes,

$$T_1{}^{-1} = \sum_{\text{process}} T_{1,\text{process}}{}^{-1} \tag{S2}$$

Common processes include spin–rotational (SR) relaxation,[20]

$$T_{1,\text{SR}}{}^{-1} = \sum_{i=x,y,z} \frac{(g_{ii} - g_e)^2}{9\tau_\text{R}}, \tag{S3}$$

modulation by g anisotropy (sometimes called the 'chemical shift anisotropy'[21,22] or CSA mechanism),[20]

$$T_{1,g}{}^{-1} = \frac{2}{5}\left(\frac{\omega}{g}\right)^2 \left\{\frac{1}{3}\left(g_{zz} - \frac{g_{xx}+g_{yy}}{2}\right)^2 + \left(\frac{g_{xx}-g_{yy}}{2}\right)^2\right\} J(\omega), \tag{S4}$$

modulation by a large anisotropic nuclear hyperfine (A) interaction (electron–nuclear dipolar, END),[20]

$$T_{1,\text{END}}{}^{-1} = \frac{2}{9} I(I+1) \sum_{i=x,y,z} \left(A_{ii} - \bar{A}\right)^2 J(\omega), \tag{S5}$$

solvent nuclear spin diffusion (SD),[22]

$$T_{1,\text{SD}}{}^{-1} = R_{\text{SD,max}} \left[\frac{2\omega\tau_\text{R}}{1 + (\omega\tau_\text{R})^{3/2}}\right]^{1/4}, \tag{S6}$$

local modes (thermal process),[23]

$$T_{1,\text{local}}{}^{-1} = C_\text{local} \frac{e^{\Delta/T}}{\left(e^{\Delta/T} - 1\right)^2}. \tag{S7}$$

and Heisenberg exchange with other radicals in solution,[24]

$$T_{1,\text{exch}}{}^{-1} = \kappa[R]. \tag{S8}$$

Here, g_{ii} ($i = x, y, z$) are the principal values of the diagonalized g matrix, g_e the free electron g value (2.0023), τ_R the rotational correlation time, ω the angular frequency of the measurement, I is the nuclear spin, A_{ii} ($i = x, y, z$) the principal values of the hyperfine A matrix, $\bar{A}$ the isotropic A value, $R_{\text{SD,max}}$ a proportionality constant for solvent spin diffusion, Δ the energy of a local mode, T the temperature, C_local a proportionality constant for the local mode of interest, $[R]$ the radical concentration, κ a proportionality constant characterizing Heisenberg exchange interactions, and J is the spectral density function,

$$J(\omega) = \frac{\tau_\text{R}}{1 + (\omega\tau_\text{R})^2}. \tag{S9}$$

Here, Eqs. (S3)–(S6) represent relaxation modes that are due to molecular tumbling; whereas, Eqs. (S7) and (S8) represent relaxation due to local modes and exchange interactions, both of which are also relevant in solid-state spin relaxation.

Assuming isotropic tumbling in solution, estimation of the rates of these various relaxation processes requires a way to estimate the rotational correlation time of the molecule. The Stokes–Einstein relation,[25]

$$\tau_\text{R} = \frac{c_\text{slip} V \eta}{k_B T}, \tag{S10}$$

can be used to relate the rotational correlation time with the hydrodynamic volume of a molecule V, the solvent viscosity η, and the temperature T. The c_{slip} parameter varies between 0 and 1, and is used to account for the strength of solvent–solute interactions, which can influence tumbling. The viscosity of 2-methyltetrahydrofuran in units of centipoise is known to vary with temperature as[26]

$$\ln(\eta\,[\text{cP}]) = -3.635 + 896.8(T\,[\text{K}])^{-1} \tag{S11}$$

over a range of 213–296 K. We have assumed this equation remains accurate over the 183–303 K range in our experiments. This was judged to be reasonable through comparison with other ethereal solvents with viscosities measured over a wider temperature range (Fig. S23a).[27] We have used ORCA 6.0.1 to estimate the volume of the relevant cations and anions: when a CPCM calculation is requested, the molecular volume is calculated by positioning spheres at each nucleus and then calculating the total enclosed volume of the molecular cavity. Radii were 1.32 Å for H, 2.04 Å for C, 1.824 Å for O, and 3.30 Å for K. The net enclosed volume was calculated to be 397 Å^3 for $[\mathbf{1}]^{\bullet-}$, 450 Å^3 for $[\mathbf{2}]^{\bullet-}$, and 504 Å^3 for $[\mathbf{3}]^{\bullet-}$. The identity of the cations were more ambiguous, but our MCD spectra did not vary with cation identity (Li^+, Na^+, K^+, Mg^{2+}) and our CW EPR spectra showed D_2 point group symmetry and only very minor changes upon addition of Kryptofix-222. Together, these observations suggest the potassium ion may not be bound to the BF anion but rather ligated as $[\text{K(THF or 2-MeTHF)}_x]^+$ ions. The volume prediction for a $[\text{K(THF)}_6]^+$ cation is 612 Å^3. If the ion pair diffuses together, as might be expected in low-dielectric solvents like 2-MeTHF, the effective molecular volume may equal the sum of the individual ion volumes. If they diffuse separately, the effective molecular volume may be more in line with the anion alone. We have plotted curves predicting the rotational correlation time as a function of temperature using a range of molecular volumes in Fig. S23b.

S3.4.2 Fitting Experimental T_1 Times

The thermal behaviors of the SR, g anisotropy, and SD mechanisms are predicted by Eqs. (S3), (S4), (S6), and (S10), and none of these mechanisms individually have temperature variations that matches well with experiment (Fig. S23c). The combination of SR and SD mechanisms (Fig. S23d) can be used to reproduce the experimental T_1 values reasonably well, but the fits give unphysical molecular volumes. Values of c_{slip} would need to be 0.04–0.08 to explain these volumes. These values are smaller than slip values previously reported for tempone in toluene (0.4) and acetone (0.5)[30] and for neutral aromatic radicals in toluene (0.2).[23] Because the 2-MeTHF solvent is somewhat polar, the BF anions are charged, and their counterions may increase the effective molecular volume, we do not consider such small c_{slip} values to be likely.

There is no single nucleus providing strong and anisotropic hyperfine coupling in these BF anions, so the END mechanism described by Eq. (S5) is expected to be a weaker contributor than in systems like TEMPO, where a ^{14}N or ^{15}N nucleus couples strongly to the electron spin. The presence of sixteen/twenty protons in the $[\mathbf{1/3}]^{\bullet-}$ anions does provide a superhyperfine pathway through which END can relax the electron spin; however, Eq. (S5) is not directly applicable to multiple nuclei.[23] Further insight will require detailed pulsed experiments (e.g. ELDOR) and modeling.

Two additional relaxation mechanisms remain to be considered: thermal (local mode) processes, and exchange interactions. The influence of a single thermal process is easy to predict, and was found to model the experimental data well. The best-fit lines for thermal processes are shown and discussed in the main manuscript. There are more intricate formulas describing spin–vibrational relaxation, such as[31–33]

$$T_{1,\text{local}}{}^{-1} = C_{\text{local}} \sum_i \left(\frac{\partial g_{\text{iso}}}{\partial Q_i}\right)^2 \frac{\exp[hc\tilde{\nu}_i/k_B T]}{(\exp[hc\tilde{\nu}_i/k_B T]-1)^2} \tag{S12}$$

where we have chosen to use the derivative of g_{iso} rather than the principal values of the g matrix due to the rapid molecular tumbling in solution. Here, i indexes each normal mode of the molecule, Q_i is the displacement along the ith normal mode, $\tilde{\nu}_i$ is the wavenumber of the ith normal mode, and C_{local} is a proportionality constant in units of $\text{Å}^2\,\text{s}^{-1}$. The performances of the simpler Eq. (S7) and more complicated Eq. (S12) were evaluated using a computational approach. Normal modes were obtained from a frequency calculation on $[\mathbf{1}]^{\bullet-}$ at the ωB97X-D3/Def2-TZVPP level of theory (see Section S5.1.1), and the `orca_pltvib` tool was used to displace the atomic coordinates forward/backward along each normal mode below 500 cm^{-1}. For each normal mode, the g_{iso} value was calculated at the PBE0/EPR-III level of theory (see Section S5.1.3)

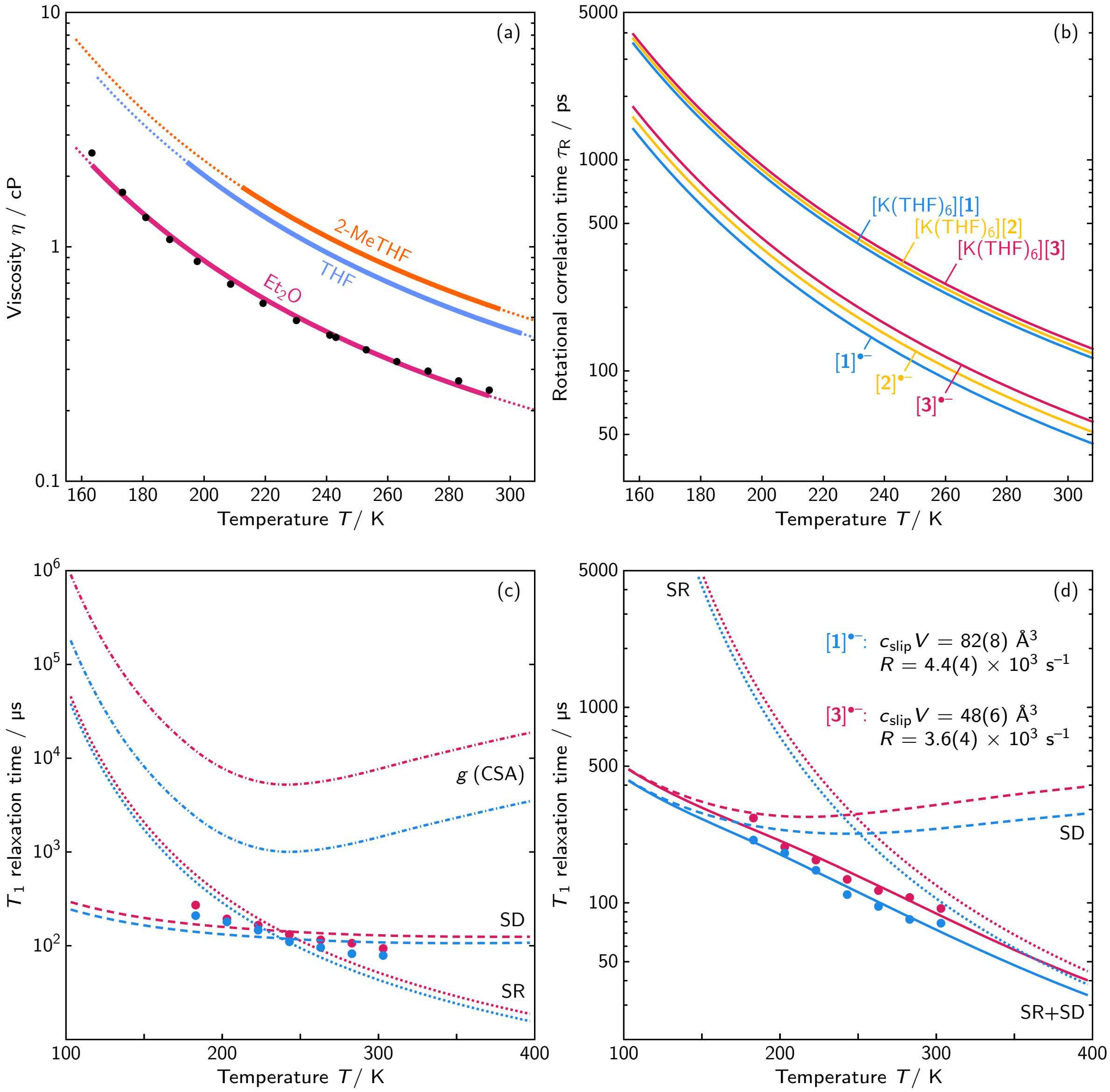

Figure S23: (a) Viscosities of three ethereal solvents are plotted as a function of temperature. The solid lines are the fitted regions[26,27] and the dotted lines are extrapolated. Data points for ether are taken from the literature[28,29] and we performed a regression to obtain the line of best fit. (b) Predicted rotational correlation times in 2-MeTHF are plotted as a function of molecular volume using the Stokes–Einstein relation, assuming $c_{\mathrm{slip}} = 1$. The molecular volumes used were 397 Å^3 for [**1**]$^{\bullet-}$, 450 Å^3 for [**2**]$^{\bullet-}$, 504 Å^3 for [**3**]$^{\bullet-}$, 1009 Å^3 for [K(THF)$_6$][**1**], 1062 Å^3 for [K(THF)$_6$][**2**], and 1116 Å^3 for [K(THF)$_6$][**3**]. (c) Best-fit lines using Eqs. (S3), (S4), and (S6) to model SR (dotted), CSA (dash-dot), and SD (dashed) mechanisms using g values in Table S16 and allowing $c_{\mathrm{slip}}V$ and $R_{\mathrm{SD,max}}$ to float. (d) Best-fit line using both SR and SD relaxation mechanisms.

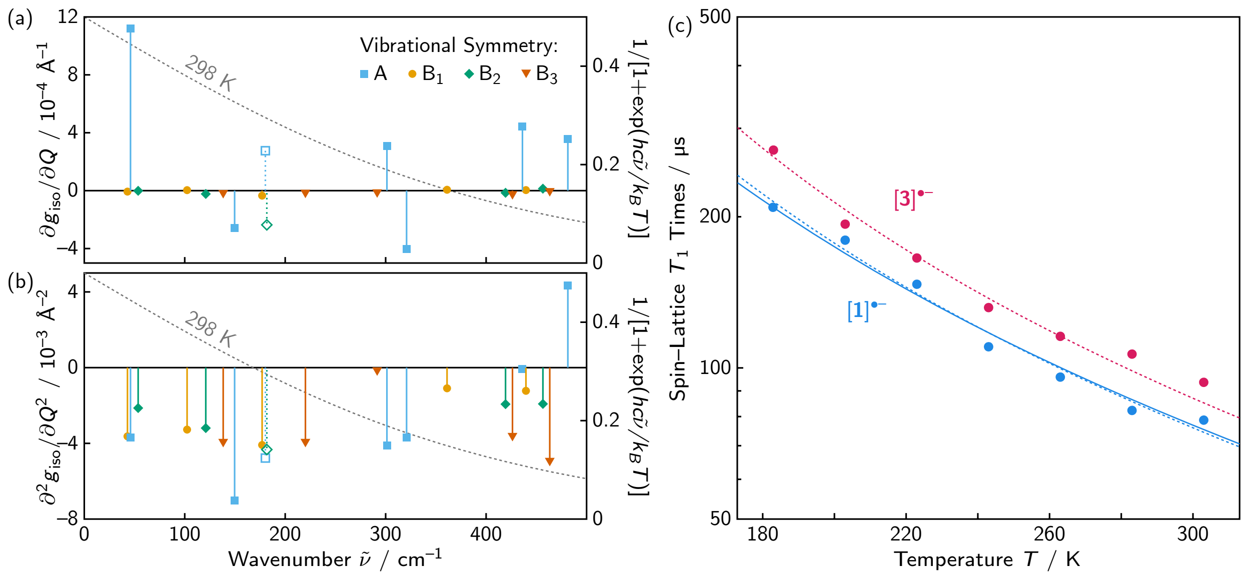

Figure S24: (a) First derivative of the molecular g_{iso} value with respect to each normal mode of $[\mathbf{1}]^{\bullet-}$ shows that only the totally symmetric normal modes ('A' irreducible representation in the D_2 point group) have appreciably large sizes. The only exception is indicated with an asterisk, where there is an 'B_2' mode strongly mixed with an 'A' mode. (b) There is not a similar irrep pattern for second derivatives because $\Gamma^2 = A$ for all irreps Γ of D_2. (c) The spin–lattice T_1 times of $[\mathbf{1}]^{\bullet-}$ and $[\mathbf{3}]^{\bullet-}$ are well described using Eq. (S7), shown by dashed lines: these best fits have $C_{\mathrm{local}} = 5(5) \times 10^{-5}$ s^{-1}, $E_a = 120(70)$ cm^{-1} for $[\mathbf{1}]^{\bullet-}$ and $C_{\mathrm{local}} = 1.1(7) \times 10^{-5}$ s^{-1}, $E_a = 190(50)$ cm^{-1} for $[\mathbf{3}]^{\bullet-}$. The best fit to Eq. (S12) for $[\mathbf{1}]^{\bullet-}$ is shown as a solid line with $C_{\mathrm{local}} = 5.0(1) \times 10^8$ Å^2/s.

at the undisplaced, forward-displaced, and backward-displaced geometries, then a quadratic regression was used to obtain the first and second numerical derivatives of g_{iso} with respect to Q_i (Table S2). These derivatives are the ones depicted in the main manuscript (Figure 8b). Here, we show the first- and second-derivatives and we compare the best fits for the two local mode equations in Fig. S24. They perform equally well, and there is a local mode calculated at 149 cm^{-1} that corresponds to the thermal behavior reasonably well.

The last relaxation pathway we consider is Heisenberg spin exchange. This is an intermolecular process that will vary with concentration of the radicals in solution. It has been observed that the relaxation times of charged radicals in solution often have far lesser responses to increases in concentration than neutral radicals.[34,35] This has been ascribed to the influence of Coulombic repulsion, which tends to keep like-charged ions away from each other, and solvation shells, which form around charged species and provide an additional spatial buffer.[36] The thermal behavior of Eq. (S8) can be captured in the change in solvent density with temperature. Thermal expansion should decrease the effective concentration as temperature rises, causing faster relaxation at lower temperatures. This is not the behavior seen.

These various considerations lead us to suggest that it is a thermal process, likely a normal mode around 150 cm^{-1}, that is the driving force of longitudinal relaxation rather than molecular tumbling or exchange.

Table S2: Derivatives of g_{iso} along each calculated normal mode of **1** below 500 cm^{-1} using ωB97X-D3/Def2-TZVP DFT

#	$\tilde{\nu}$ / cm^{-1}	Γ	$\frac{\partial g_{\text{iso}}}{\partial Q}$ / 10^{-4} Å^{-1}	$\frac{\partial^2 g_{\text{iso}}}{\partial Q^2}$ / 10^{-3} Å^{-2}
6	43.00	B_1	−0.0660	−3.62945
7	46.03	A	11.1883	−3.69913
8	53.65	B_2	−0.0110	−2.13411
9	102.35	B_1	0.0352	−3.27328
10	120.95	B_2	−0.2266	−3.19294
11	138.29	B_3	−0.1518	−3.91303
12	149.66	A	−2.5738	−7.02661
13	177.03	B_1	−0.3432	−4.08627
14	180.27	*	2.7586	−4.77732
15	181.71	*	−2.3626	−4.32823
16	220.13	B_3	−0.1298	−3.91109
17	291.55	B_3	−0.1034	−0.13453
18	301.27	A	3.0732	−4.11821
19	320.83	A	−4.0433	−3.69332
20	361.11	B_1	0.0616	−1.09174
21	419.32	B_2	−0.1474	−1.92893
22	426.32	B_3	−0.2310	−3.59751
23	436.16	A	4.4437	−0.06194
24	439.64	B_1	0.0396	−1.22530
25	456.53	B_2	0.1364	−1.91635
26	463.34	B_3	−0.0176	−4.91475
27	481.36	A	3.5571	+4.33307

* These two modes are strongly mixed between one of 'A' symmetry and one of 'B_2' symmetry.

S4 X-ray Crystallography

The crystals were mounted in hydrocarbon oil on a nylon loop or a glass fiber or they were sealed into glass capillaries ([K(THF)$_4$][**1**]). Data were collected on a Bruker D8 Venture Duo diffractometer coupled to a Photon III CPAD with Mo Kα radiaton (λ = 0.71073 Å) with ϕ- and ω-scans. Data were indexed with Apex II (Difference Vectors method), integrated with Bruker SAINT, and a semi-empirical absorption correction was applied using SADABS or TWINABS. The space group was established using XPREP, and the structure was solved by intrinsic phasing methods using SHELXT-2018/2 and refined against F^2 on all data by full-matrix least squares with SHELXL-2019/2 using established methods. All non-hydrogen atoms were refined anisotropically. All hydrogen atoms were included in the model at geometrically calculated positions and refined using a riding model unless otherwise noted. The isotropic displacement parameters of all hydrogen atoms were fixed to 1.2 times the U_{eq} value of the atoms they are linked to (1.5 times for methyl groups).

Table S3: Summary of crystallographic data

	**2**	[K(THF)$_4$][**1**]	[K(THF)$_4$][**2**]
CCDC	CSD 2533122	CSD 2545211	CSD 2533123
Empirical formula	$C_{30}H_{18}$	$C_{42}H_{48}KO_4$	$C_{46}H_{50}KO_4$
Formula weight (g/mol)	378.44	655.90	705.96
Color / Morphology	red / block	dark green / block	brown / block
Crystal size (mm^3)	$0.095 \times 0.050 \times 0.046$	$0.831 \times 0.320 \times 0.288$	$0.184 \times 0.108 \times 0.100$
Temperature (K)	150(2)	304(2)	293(2)
Wavelength (Å)	1.54178	0.71073	0.71073
Crystal system, Space group	Monoclinic, $P2_1/c$	Monoclinic, $C2/c$	Triclinic, $P\bar{1}$
Unit cell dimensions (Å, °)	$a = 19.7948(8)$, $\alpha = 90$	$a = 26.1415(15)$, $\alpha = 90$	$a = 9.3320(7)$, $\alpha = 81.852(4)$
	$b = 5.0231(2)$, $\beta = 117.501(2)$	$b = 9.7023(5)$, $\beta = 126.820(2)$	$b = 12.4024(9)$, $\beta = 88.204(4)$
	$c = 21.4227(8)$, $\gamma = 90$	$c = 18.6702(11)$, $\gamma = 90$	$c = 17.6333(13)$, $\gamma = 69.094(5)$
Volume (Å^3)	1889.39(13)	3790.8(4)	1886.9(2)
Z	4	4	2
Density (calc., g/cm^3)	1.330	1.149	1.243
Absorption coefficient (mm^{-1})	0.573	0.179	0.184
$F(000)$	792	1404	754
Theta range for data collection (°)	2.516 to 68.786	2.204 to 25.027	1.167 to 25.356
Index ranges	$-23 \leq h \leq 22$, $-6 \leq k \leq 6$,	$-31 \leq h \leq 31$, $-11 \leq k \leq 11$,	$-11 \leq h \leq 11$, $-14 \leq k \leq 14$,
	$-25 \leq l \leq 25$	$-22 \leq l \leq 22$	$0 \leq l \leq 21$
Reflections collected	31672	31877	6871
Independent reflections, R_{int}	3442, 0.1961	3343, 0.0931	6871, 0.1096
Completeness to $\theta_{\max}$ (%)	99.4	99.9	99.9
Absorption correction	Multi-Scan	Semi-empirical from equivalents	Semi-empirical from equivalents
Refinement method	Full-matrix least squares on F^2	Full-matrix least squares on F^2	Full-matrix least squares on F^2
Data / Restraints / Parameters	3442 / 0 / 271	3343 / 1799 / 399	6871 / 1494 / 489
Goodness-of-fit[a]	1.140	1.112	1.672
Final R indices[b] $[I > 2\sigma(I)]$	$R_1 = 0.0628$, $wR_2 = 0.1708$	$R_1 = 0.0885$, $wR_2 = 0.1833$	$R_1 = 0.1134$, $wR_2 = 0.3030$
R indices[b] (all data)	$R_1 = 0.1444$, $wR_2 = 0.2395$	$R_1 = 0.1416$, $wR_2 = 0.2109$	$R_1 = 0.1431$, $wR_2 = 0.3388$
Largest diff. peak and hole (e·Å^{-3})	0.377 and −0.445	0.212 and −0.214	0.957 and −0.395

[a] Goodness-of-fit $= \sqrt{\frac{\sum[w(F_o^2-F_c^2)^2]}{(n-p)}}$ [b] $R_1 = \frac{\sum||F_o|-|F_c||}{\sum|F_o|}$; $wR_2 = \sqrt{\frac{\sum[w(F_o^2-F_c^2)^2]}{\sum[w(F_o^2)^2]}}$; $w = \frac{1}{\sigma^2(F_o^2)+(aP)^2+bP}$; $P = \frac{2F_c^2+\max(F_o^2,0)}{3}$

Table S4: Influence of Basis Set Choice on Thermochemistry[a] of **1** and $[\mathbf{1}]^{\bullet-}$

	Basis Set	$\Delta E^{\ddagger}$	$\Delta H^{\ddagger}$	$\Delta S^{\ddagger}$	$\Delta G^{\ddagger}$	$\tilde{\nu}$(C=C)[b] / cm^{-1}
Neutral **1**	Def2-SVP	+19.61	+17.21	−1.10	+17.54	1684, 1716
	Def2-TZVP	+20.18	+17.66	−1.03	+17.97	1663, 1697
	Def2-TZVPP	+20.16	+17.66	−1.05	+17.97	1662, 1698
	Def2-QZVP	+20.18	+17.71	−0.93	+17.99	1660, 1696
	Def2-QZVPP	+20.18	+17.71	−0.92	+17.99	1661, 1696
	ma-Def2-SVP	+18.92	+16.72	−2.63	+17.50	1680, 1712
	ma-Def2-TZVP	+20.17	+17.72	−0.72	+17.93	1660, 1696
	ma-Def2-TZVPP	+20.15	+17.68	−0.70	+17.89	1662, 1697
Anion $[\mathbf{1}]^{\bullet-}$	Def2-SVP	+12.36	+11.10	−1.57	+11.57	1612
	Def2-TZVP	+12.08	+10.74	−1.50	+11.19	1587
	Def2-TZVPP	+12.04	+10.73	−1.54	+11.20	1587
	Def2-QZVP	+11.97	+10.69	−1.32	+11.08	1586
	Def2-QZVPP	+11.97	+10.69	−1.33	+11.08	1586
	ma-Def2-SVP	+11.61	+10.55	−2.77	+11.38	1608
	ma-Def2-TZVP	+12.02	+10.75	−0.85	+11.00	1586
	ma-Def2-TZVPP	+12.00	+10.73	−0.82	+10.97	1586

[a] All energies in kcal/mol and all entropies in cal/mol·K. Gibbs free energies are reported at 298.15 K. [b] The neutral species had two normal modes with significant C=C character around 1700 cm^{-1}.

S5 Computational Methods

All calculations were performed using Orca 6.0.1.[37–46]

S5.1 Density Functional Theory (DFT) Calculations

S5.1.1 Geometry Optimizations, Transition States, and Thermochemistry

Initial geometries were built in Avogadro and aligned so that the central olefinic C=C bond was along the x axis and the normal vector to the face of the olefin was along the z axis. Geometries were optimized using the ωB97X-D3 density function, the Def2-TZVPP basis set, a conductor-like polarizable continuum model to describe THF solvation. Calculations were performed using the default RIJCOSX approximation and the Def2/J auxiliary basis set on all atoms. Optimizations of **1** and its anion were performed within the D_2 point group. Optimizations of E-**3**, Z-**3**, and their anions were performed within the C_2 point group. Optimizations of **2** and its anion were not conducted using symmetry.

Transition states for $E \rightleftharpoons Z$ isomerization were constructed in Avogadro with a 90° twist angle, and the molecule was carefully aligned to match the orientations of the E and Z local minima. Transition state calculations were all performed as broken-symmetry singlets. An initial optimization was performed while constraining the twist dihedral angle to be 90°, then a frequency calculation and transition state optimization were used to obtain an accurate geometry.

We sought to explore whether our density functional theory was providing useful thermochemical predictions for our compounds. We explored the influence of basis sets on these thermochemical values by screening the calculated barrier for **1** and $[\mathbf{1}]^{\bullet-}$ using a variety of basis sets (Table S4). The choice of basis set had only a minor impact, and seemed totally converged to a stable value as soon as a triple-zeta basis was used. Thermochemical barriers for all compounds at the ωB97X-D3/Def2-TZVPP level of theory are summarized in Table S5. Comparison of the barrier for **2** with the experimental number from our VT EXSY experiment showed that this level of theory was underestimating the barrier. Table S6 summarizes the barriers obtained using various other density functionals, and the barrier to the broken-symmetry 90° transition was was underestimated by all functionals. This underestimation has been previously observed in DFT for BF molecules.[6]

Table S5: Thermochemistry for all compounds

Reaction	ωB97X-D3/Def2-TZVPP				NEVPT2	Hybrid		
	$\Delta E^\ddagger$	$\Delta H^\ddagger$	$\Delta S^\ddagger$	$\Delta G^\ddagger$	$\Delta E^\ddagger$	$\Delta H^\ddagger$	$\Delta S^\ddagger$	$\Delta G^\ddagger$
**1** → TS	+20.16	+17.66	−1.05	+17.97	+29.5	+27.0	−1.05	+27.3
[**1**]$^{\bullet-}$ → TS	+12.04	+10.73	−1.54	+11.20	+16.8	+15.5	−1.54	+16.0
**2** → TS	+15.57	+13.12	−0.94	+13.40	+25.5	+23.1	−0.94	+23.3
[**2**]$^{\bullet-}$ → TS	+7.50	+6.41	−2.04	+7.02	+13.3	+12.2	−2.04	+12.8
E-**3** → TS	+11.84	+9.43	−0.93	+9.70	+22.3	+19.9	−0.93	+20.2
[*E*-**3**]$^{\bullet-}$ → TS	+6.47	+5.44	−2.30	+6.13	+12.8	+11.8	−2.30	+13.0
Reaction	ΔE°	ΔH°	ΔS°	ΔG°	ΔE°	ΔH°	ΔS°	ΔG°
E-**3** → *Z*-**3**	+1.09	+1.07	+0.28	+0.99	+0.0	+0.0	+0.28	−0.1
[*E*-**3**]$^{\bullet-}$ → [*Z*-**3**]$^{\bullet-}$	+0.06	+0.18	−0.49	+0.33	−0.3	−0.2	−0.49	+0.0

All energies in kcal/mol and all entropies in cal/mol·K. Gibbs free energies are reported at 298.15 K. Hybrid numbers were calculated as $\Delta H^\ddagger_{\text{Hybrid}} = \Delta H^\ddagger_{\text{DFT}} - \Delta E^\ddagger_{\text{DFT}} + \Delta E^\ddagger_{\text{NEVPT2}}$, $\Delta S^\ddagger_{\text{Hybrid}} = \Delta S^\ddagger_{\text{DFT}}$, and $\Delta G^\ddagger_{\text{Hybrid}} = \Delta H^\ddagger_{\text{Hybrid}} - T\Delta S^\ddagger_{\text{Hybrid}}$.

Table S6: Screening Density Functionals for *E*/*Z* Isomerization Barrier of **2**

Functional	$\Delta E^\ddagger$	$\Delta H^\ddagger$	$\Delta S^\ddagger$	$\Delta G^\ddagger$
ωB97X-D3	+15.57	+13.12	−0.94	+13.40
BP-D3(BJ)	+19.46	+17.64	−0.19	+17.69
PBE0-D3(BJ)	+16.83	+14.69	−0.58	+14.87
TPSS-D3(BJ)	+18.70	+16.76	−0.22	+16.82
TPSSh-D3(BJ)	+17.70	+15.64	−0.40	+15.76
EXSY NMR		+19.52	−4.75	+20.91

All energies in kcal/mol and all entropies in cal/mol·K. Gibbs free energies are reported at 298.15 K.

Table S7: Basis set impact on TD-DFT transition energies (cm^{-1})

	Basis Set	State									
		1	2	3	4	5	6	7	8	9	10
Neutral **1**	Def2-SVP	24964	28280	28300	36458	36985	38577	38709	43036	43589	44056
	Def2-TZVP	24928	28377	28392	36517	37030	38234	38544	42400	43040	44013
	Def2-TZVPP	24923	28375	28391	36510	37023	38221	38534	42382	43023	44007
	Def2-QZVP	24909	28398	28410	36494	37003	38148	38468	42234	42888	43962
	Def2-QZVPP	24909	28398	28410	36494	37003	38148	38468	42234	42888	43962
	ma-Def2-SVP	24542	28099	28101	36129	36691	38029	38262	42155	42753	43602
	ma-Def2-TZVP	24866	28355	28367	36457	36968	38114	38422	42142	42797	43887
	ma-Def2-TZVPP	24860	28352	28364	36448	36961	38097	38411	42122	42778	43880
Anion [**1**]$^{\bullet-}$	Def2-SVP	14688	21771	22442	24623	24701	27405	28145	31434	31448	31515
	Def2-TZVP	14658	21186	21964	24627	24707	27068	27969	31096	31103	31108
	Def2-TZVPP	14650	21173	21956	24624	24704	27055	27958	31085	31091	31092
	Def2-QZVP	14638	21073	21852	24614	24697	26903	27879	30997	31035	31041
	Def2-QZVPP	14638	21073	21852	24614	24697	26903	27879	30997	31035	31041
	ma-Def2-SVP	14215	21314	22017	24355	24461	26828	27800	30873	31035	31044
	ma-Def2-TZVP	14601	21032	21778	24583	24666	26657	27821	29162	30932	30991
	ma-Def2-TZVPP	14594	21016	21768	24578	24662	26637	27808	29150	30913	30979

All calculations were performed using the ωB97X-D3 density function and a CPCM(THF) solvation treatment.

Due to the open-shell nature of the transition state and the close proximity of a triplet state at the 90° geometry, we turned to a multireference calculation to explore the potential energy surface. The energies $\Delta E^{\ddagger}$ obtained from CASSCF(2,2)/RI-NEVPT2 for the neutral species or CASSCF(3,2)/RI-NEVPT2 for the anion species (see Section S5.2.1) were combined with the enthalpic corrections calculated from ωB97X-D3/Def2-TZVPP frequency calculations to obtain the final numbers reported in the main manuscript. The multireference and hybrid energies are also summarized in Table S5. This hybrid technique was necessary because the lack of analytic gradients for NEVPT2 calculations prevented thermochemical calculations rooted solely in multireference calculations.

S5.1.2 Time-Dependent DFT Calculations

Time-dependent DFT calculations were performed for all species. The Tamm–Dancoff approximation was used throughout.[47] Initial calculations were conducted at the same level of the geometry optimizations, ωB97X-D3/Def2-TZVPP with CPCM(THF). The influence of the basis set was explored for both **1** and [**1**]$^{\bullet-}$. The transition energies for the first ten states are summarized in Table S7. The Def2-TZVPP basis set was selected as a good compromise between size and speed.

While the ωB97X-D3 density functional performed fairly well, we found the SOS-PBE-QIDH functional to perform better. Results in the main manuscript are reported at the SOS-PBE-QIDH/Def2-TZVPP level of theory using a CPCM(THF) solvation treatment. Forty roots were requested for all calculations, and the first twenty are summarized in Tables S8–S15. Symmetry assignments listed in the main document were made by inspecting the natural orbitals involved, and double checked through comparison with the transition dipole moment directions. Lineshape calculations for **1** and [**1**]$^{\bullet-}$ were also explored using the Excited State Dynamics module at the ωB97X-D3/Def2-TZVP level of theory.

Table S8: Calculated transitions for **1**

$\tilde{\nu}$ / cm^{-1}	λ / nm	f_{osc}	D^2 / au^2	D_x / au	D_y / au	D_z / au
23650.8	422.8	0.834265754	11.61271	−3.40774	−0.00134	−0.00193
25080.7	398.7	0.000811558	0.01065	−0.00012	0.00237	0.10318
25119.9	398.1	0.004555599	0.05970	0.00083	0.24434	−0.00101
32712.9	305.7	0.007069692	0.07115	−0.00083	0.00002	0.26673
33228.1	301.0	0.007360733	0.07293	0.00029	0.27005	−0.00007
34190.0	292.5	0.024788510	0.23869	−0.48856	0.00013	−0.00001
34560.1	289.4	0.000000029	0.00000	−0.00004	−0.00008	−0.00052
38994.7	256.4	0.000000311	0.00000	−0.00022	−0.00108	−0.00119
39156.1	255.4	0.291103721	2.44751	0.00286	0.00010	−1.56445
39542.3	252.9	1.588740672	13.22715	−0.00336	3.63691	0.00025
40697.1	245.7	0.657963977	5.32248	2.30703	0.00299	0.00942
42986.0	232.6	0.051540036	0.39472	0.02950	0.00093	−0.62758
44390.7	225.3	0.000002038	0.00002	−0.00020	−0.00353	0.00162
44526.3	224.6	0.000566289	0.00419	−0.00014	0.00168	−0.06468
44680.2	223.8	0.018477674	0.13615	0.00254	−0.36897	−0.00032
45035.8	222.0	0.277632781	2.02950	−1.42460	0.00307	−0.00077
45321.5	220.6	0.988114237	7.17759	−0.00129	2.67910	0.00002
45594.7	219.3	0.046362246	0.33475	0.00169	−0.02212	−0.57815
45696.7	218.8	0.503815993	3.62963	0.00018	−1.90515	0.00638
45887.4	217.9	0.000000107	0.00000	0.00060	−0.00007	−0.00064

Table S9: Calculated transitions for $[\mathbf{1}]^{\bullet -}$

$\tilde{\nu}$ / cm^{-1}	λ / nm	f_{osc}	D^2 / au^2	D_x / au	D_y / au	D_z / au
13287.7	752.6	0.305581588	7.57102	−2.75155	−0.00053	−0.00068
19463.7	513.8	0.000000102	0.00000	−0.00060	0.00116	−0.00019
20676.1	483.6	0.006634047	0.10563	−0.32501	−0.00004	−0.00040
24167.1	413.8	0.000117881	0.00161	0.00026	−0.00087	−0.04006
24233.3	412.7	0.001886606	0.02563	−0.00010	0.16009	−0.00019
25532.3	391.7	0.067567939	0.87122	−0.00071	0.93339	−0.00012
26398.4	378.8	0.092078402	1.14830	1.07159	0.00023	0.00005
26507.4	377.3	0.009062806	0.11256	0.00064	−0.00005	0.33549
27930.4	358.0	0.000000181	0.00000	−0.00030	0.00142	0.00014
29356.5	340.6	0.028044652	0.31450	0.00022	−0.56080	−0.00174
29361.3	340.6	0.005401301	0.06056	0.00131	0.00393	−0.24606
30617.1	326.6	0.212004997	2.27959	1.50983	0.00029	−0.00029
31713.9	315.3	0.029399500	0.30519	0.00258	0.00010	−0.55243
32802.3	304.9	0.082238539	0.82537	0.00018	−0.90850	0.00017
33445.1	299.0	0.004630194	0.04558	0.00070	0.21349	−0.00016
33922.4	294.8	0.000312881	0.00304	0.00118	−0.00048	−0.05509
34100.9	293.2	0.000000206	0.00000	0.00101	−0.00098	0.00006
34439.3	290.4	0.020750696	0.19836	−0.44536	0.00044	0.00344
34749.1	287.8	0.000004987	0.00005	−0.00004	0.00687	0.00026
34825.0	287.2	0.291786211	2.75835	−0.00026	1.66083	−0.00016

Table S10: Calculated transitions for **2**

$\tilde{\nu}$ / cm^{-1}	λ / nm	f_{osc}	D^2 / au^2	D_x / au	D_y / au	D_z / au
22157.0	451.3	0.683745785	10.15918	−3.16965	0.30847	−0.13175
22768.0	439.2	0.045619239	0.65963	−0.78432	0.10124	−0.18498
24376.0	410.2	0.001675926	0.02263	−0.07270	−0.10206	−0.08326
28752.5	347.8	0.077921233	0.89219	0.09395	0.87679	−0.33853
31975.5	312.7	0.056891357	0.58574	−0.01381	0.76344	0.05205
33280.1	300.5	0.125566379	1.24212	0.13801	−0.96042	0.54833
33860.5	295.3	0.006889522	0.06698	−0.23157	0.08542	−0.07788
35416.0	282.4	0.355260305	3.30235	−0.55375	−1.50449	0.85570
36448.7	274.4	0.072411922	0.65404	0.61272	−0.48014	−0.21928
37511.4	266.6	0.587723885	5.15805	−0.70323	−1.33358	1.69855
38384.1	260.5	0.560671855	4.80876	1.48781	1.58446	−0.29096
38710.1	258.3	0.088772327	0.75497	−0.52230	−0.63198	0.28770
38800.1	257.7	1.052379318	8.92926	−0.15942	2.93865	0.51784
40470.2	247.1	0.042447716	0.34530	−0.57124	−0.06214	−0.12299
42263.8	236.6	0.381182223	2.96920	−1.02665	1.38174	0.07748
42893.9	233.1	0.117401309	0.90106	0.62624	−0.64680	−0.30089
43526.5	229.7	0.056020918	0.42371	−0.24549	0.28276	0.53244
43796.0	228.3	0.373298674	2.80607	0.86068	1.42637	0.17537
43803.9	228.3	0.123362859	0.92714	−0.48916	−0.30679	−0.77055
44129.9	226.6	0.079818410	0.59545	−0.75466	0.04469	0.15473

Table S11: Calculated transitions for [**2**]$^{\bullet-}$

$\tilde{\nu}$ / cm^{-1}	λ / nm	f_{osc}	D^2 / au^2	D_x / au	D_y / au	D_z / au
12115.6	825.4	0.253827470	6.89712	−2.62066	0.15557	−0.07102
20727.1	482.5	0.059971493	0.95254	0.58740	−0.70228	0.33807
20786.9	481.1	0.016276644	0.25778	−0.33108	0.32936	−0.19923
21539.4	464.3	0.010230154	0.15636	−0.29982	0.25247	−0.05222
22170.4	451.1	0.024591638	0.36517	0.24739	−0.50908	0.21167
22925.9	436.2	0.000267122	0.00384	−0.00399	0.05762	−0.02235
26710.6	374.4	0.045690289	0.56314	−0.30965	0.62323	−0.28079
27082.1	369.2	0.055085827	0.66963	0.60954	0.38909	−0.38301
27915.1	358.2	0.027963202	0.32978	0.31828	0.47623	0.04106
28418.2	351.9	0.044387463	0.51421	0.56677	−0.43010	0.08941
28531.6	350.5	0.022613393	0.26092	−0.33108	−0.37150	−0.11533
28858.3	346.5	0.048541977	0.55376	−0.41199	0.48056	−0.39126
29082.0	343.9	0.004044975	0.04579	0.15300	0.04209	−0.14356
31241.9	320.1	0.080390551	0.84712	0.07433	0.85956	0.32055
31508.1	317.4	0.048279979	0.50445	−0.18033	−0.59161	0.34918
32379.6	308.8	0.011198917	0.11386	0.22724	0.24934	−0.00722
32645.9	306.3	0.011486735	0.11584	−0.24017	0.22494	−0.08693
32665.9	306.1	0.025403363	0.25602	0.38071	−0.17592	−0.28307
33103.8	302.1	0.005342900	0.05313	0.12146	−0.16465	0.10616
34481.2	290.0	0.008192694	0.07822	0.17878	−0.14161	−0.16188

Table S12: Calculated transitions for *E*-**3**

$\tilde{\nu}$ / cm^{-1}	λ / nm	f_{osc}	D^2 / au^2	D_x / au	D_y / au	D_z / au
20930.8	477.8	0.612688381	9.63673	−3.03342	0.65961	−0.00499
21868.9	457.3	0.002494300	0.03755	−0.00003	0.00046	0.19378
22140.0	451.7	0.050225394	0.74683	0.84520	−0.18020	0.00130
27797.3	359.7	0.149901723	1.77533	−0.08906	−1.32944	0.00207
28778.7	347.5	0.025669155	0.29364	−0.00076	0.00062	0.54189
32391.6	308.7	0.067426431	0.68529	0.00174	−0.00494	−0.82781
32845.5	304.5	0.003873954	0.03883	0.00041	−0.00119	−0.19705
32904.7	303.9	0.227569024	2.27683	0.23443	−1.49059	0.00487
35049.0	285.3	0.001874404	0.01761	−0.00032	−0.00023	0.13269
36271.1	275.7	0.533579282	4.84299	0.00356	−0.00373	−2.20067
36447.7	274.4	0.753124157	6.80256	0.78920	2.48590	−0.00358
37245.9	268.5	1.071522128	9.47106	−1.63170	−2.60933	0.00233
37316.5	268.0	0.223414993	1.97100	0.62857	−1.25534	0.00336
37693.9	265.3	0.046625552	0.40722	0.00291	−0.00256	−0.63813
38351.3	260.7	0.098891720	0.84890	0.92123	−0.01481	0.00256
38360.8	260.7	0.018037038	0.15479	−0.00002	−0.00063	−0.39344
39397.3	253.8	1.048894210	8.76476	0.43409	2.92853	−0.00521
42410.7	235.8	0.117662024	0.91335	0.89916	−0.32382	0.00157
42434.5	235.7	0.009338605	0.07245	−0.00011	−0.00006	0.26917
42439.9	235.6	0.007837600	0.06080	0.00128	−0.00035	−0.24657

Table S13: Calculated transitions for $[E\text{-}\mathbf{3}]^{\bullet -}$

$\tilde{\nu}$ / cm^{-1}	λ / nm	f_{osc}	D^2 / au^2	D_x / au	D_y / au	D_z / au
11111.8	899.9	0.235841135	6.98734	2.62245	−0.33176	0.00373
19663.0	508.6	0.004667540	0.07815	−0.22583	0.16476	−0.00065
20166.4	495.9	0.000832858	0.01360	0.00065	−0.00059	−0.11660
20574.4	486.0	0.113137733	1.81033	−0.81964	1.06701	−0.00295
22571.5	443.0	0.000828475	0.01208	−0.00083	0.00109	0.10992
23638.5	423.0	0.042172505	0.58733	−0.43436	0.63140	−0.00153
23679.0	422.3	0.007071454	0.09832	−0.00008	−0.00015	−0.31355
26544.1	376.7	0.035280017	0.43756	−0.00161	0.00236	0.66148
27386.8	365.1	0.108898149	1.30904	−0.52852	−1.01475	0.00089
27673.7	361.4	0.029362605	0.34930	0.32932	−0.49076	0.00277
28109.0	355.8	0.005240618	0.06138	0.00068	−0.00042	−0.24774
28237.4	354.1	0.001664927	0.01941	−0.00044	−0.00021	0.13932
28800.7	347.2	0.012555380	0.14352	−0.21763	−0.31008	0.00033
29341.6	340.8	0.097394885	1.09277	−0.58980	0.86308	−0.00199
29807.7	335.5	0.046351570	0.51193	−0.00100	0.00131	0.71549
30970.4	322.9	0.054522146	0.57957	0.02061	0.76093	−0.01152
31239.4	320.1	0.031570231	0.33270	−0.01141	−0.57669	0.00111
31313.1	319.4	0.002388132	0.02511	0.00039	−0.00037	−0.15845
31761.3	314.8	0.016479144	0.17081	−0.00012	0.01910	0.41285
31967.3	312.8	0.001185444	0.01221	0.00032	0.00160	−0.11048

Table S14: Calculated transitions for *Z*-**3**

$\tilde{\nu}$ / cm^{-1}	λ / nm	f_{osc}	D^2 / au^2	D_x / au	D_y / au	D_z / au
20205.5	494.9	0.584183694	9.51819	3.06936	−0.31173	0.00607
21068.8	474.6	0.000679454	0.01062	−0.00062	−0.00021	0.10304
22095.3	452.6	0.038215997	0.56941	0.72968	−0.19229	0.00122
27005.2	370.3	0.155060182	1.89029	−0.00333	−0.00475	1.37487
28519.5	350.6	0.033139274	0.38254	−0.05086	−0.61640	−0.00267
32274.1	309.8	0.061263996	0.62492	0.04114	0.78945	0.00124
32351.7	309.1	0.194073541	1.97490	−0.00347	−0.00426	1.40530
34329.5	291.3	0.002937141	0.02817	0.00141	0.00173	−0.16781
34468.2	290.1	0.086741058	0.82848	0.79431	0.44444	0.00451
35250.4	283.7	0.235470563	2.19912	0.00501	0.00707	−1.48292
35931.5	278.3	0.329880052	3.02243	1.18952	1.26784	0.00832
36907.0	271.0	0.259243499	2.31246	0.53178	1.42466	0.00338
37283.2	268.2	1.736262291	15.33127	0.00827	0.01253	−3.91549
37788.2	264.6	0.026298346	0.22911	−0.02542	−0.01464	0.47776
37826.2	264.4	0.174877347	1.52201	1.22310	0.16137	0.00147
38025.5	263.0	0.274336184	2.37511	0.00479	0.00641	−1.54112
38242.2	261.5	0.254617882	2.19190	−1.31038	−0.68888	−0.01612
40439.8	247.3	0.103406131	0.84181	−0.39318	0.82898	0.00193
41728.2	239.6	0.027484095	0.21683	−0.45606	0.09405	−0.00149
41745.8	239.5	0.101699757	0.80202	−0.00166	0.00299	−0.89555

Table S15: Calculated transitions for [*Z*-**3**]$^{\bullet -}$

$\tilde{\nu}$ / cm^{-1}	λ / nm	f_{osc}	D^2 / au^2	D_x / au	D_y / au	D_z / au
10528.9	949.8	0.226206690	7.07289	−2.65328	0.18160	−0.00546
19457.3	513.9	0.000127118	0.00215	0.00109	−0.00095	0.04635
19647.2	509.0	0.001550382	0.02598	−0.14198	0.07630	−0.00027
22239.7	449.6	0.029429884	0.43565	0.52775	−0.39607	0.01601
22758.2	439.4	0.001676630	0.02425	−0.06572	0.04879	0.13249
23263.6	429.9	0.039023972	0.55224	−0.58570	0.45734	−0.00604
23504.9	425.4	0.033233512	0.46547	0.00777	−0.00160	−0.68221
26228.4	381.3	0.083159665	1.04380	0.00541	0.00335	−1.02164
26743.7	373.9	0.054544684	0.67144	−0.61808	−0.53781	0.01317
26780.0	373.4	0.033794913	0.41545	0.51783	−0.38378	0.00299
28207.9	354.5	0.018639429	0.21754	0.00156	0.00015	−0.46641
28288.4	353.5	0.030319244	0.35285	0.01561	0.01182	0.59369
29248.4	341.9	0.041817805	0.47069	0.35349	−0.58799	−0.00069
29403.5	340.1	0.020013169	0.22407	−0.45920	0.11490	−0.00177
30087.9	332.4	0.139692822	1.52847	−0.00340	−0.00538	1.23630
30200.2	331.1	0.007608686	0.08294	0.00140	0.00443	−0.28796
30766.0	325.0	0.014310733	0.15313	0.15171	0.36067	0.00571
31184.6	320.7	0.029959762	0.31628	0.02325	0.31972	−0.46208
31269.9	319.8	0.034090814	0.35891	−0.01787	−0.27573	−0.53157
32966.5	303.3	0.093012183	0.92884	−0.28391	0.92090	0.01336

Table S16: Calculated BF Radical Anion g Values

Species	g_{xx}	g_{yy}	g_{zz}	g_{iso}
$[\mathbf{1}]^{\bullet-}$	2.0021874	2.0022919	2.0032372	2.0025722
$[\mathbf{2}]^{\bullet-}$	2.0020531	2.0026302	2.0034745	2.0027192
$[E\text{-}\mathbf{3}]^{\bullet-}$	2.0024366	2.0029266	2.0029865	2.0027832
$[Z\text{-}\mathbf{3}]^{\bullet-}$	2.0022396	2.0025268	2.0026416	2.0024694

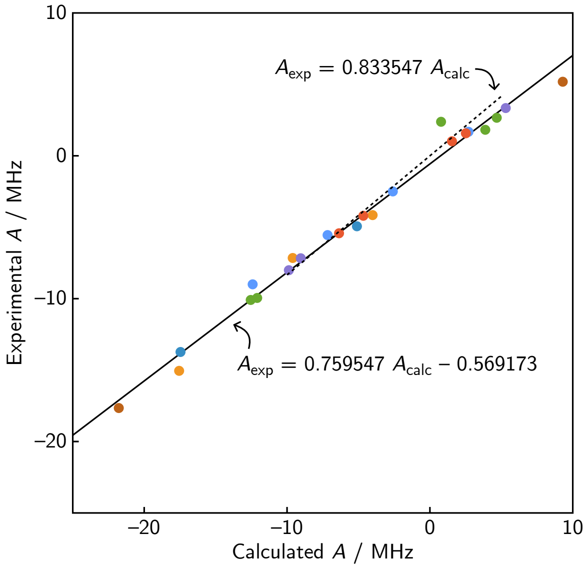

Figure S25: The calculated EPR isotropic hyperfine constants correlated strongly with the experimental ones, but were overestimated (Table S17). The line of best fit ($A_{\text{exp}} = 0.759547A_{\text{calc}} - 0.569173$) is also shown.

S5.1.3 EPR Property Calculations

EPR calculations were performed on geometries optimized at the ωB97X-D3/Def2-TZVPP level of theory with a CPCM(THF) solvation treatment. Spin Hamiltonian parameters were calculated at the PBE0/EPR-III level of theory. The g values matched experiment well (Table S16), but the hyperfine constants appeared to be somewhat overestimated at this level of theory. Correlations between calculated and experimental isotropic hyperfine constants were obtained in two ways (Fig. S25): (1) a scaling constant between the two was estimated by fitting the general lineshape of our experimental EPR spectrum of $[\mathbf{3}]^{\bullet-}$ as described in Section S3.1, giving a relation of $A_{\text{exp}} \sim 0.833547A_{\text{calc}}$, and (2) linear regression was performed between calculated and experimental A_{iso} values for a series of polyaromatic systems (Table S17), giving $A_{\text{exp}} \sim 0.759547A_{\text{calc}} - 0.569173$. Results for the bifluorenylidene molecules **1**, **2**, E-**3**, and Z-**3** are summarized in Table S18.

Table S17: Calculated vs Experimental Isotropic ^{1}H Hyperfine Constants (MHz)

Compound	#	Calc	Exp
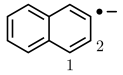	1	−17.4593	13.73
	2	−5.2484	5.07
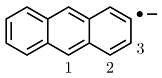	1	−17.5543	15.05
	2	−9.7449	7.29
	3	−4.1513	4.29
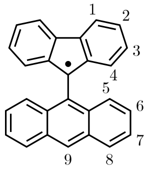	1	+3.7360	1.68
	2	−12.5435	10.09
	3	+4.5382	2.52
	4	−12.2311	10.09
	5	+0.6401	
	6	−0.4629	
	7	+0.6528	
	8	−0.4007	
	9	+0.6401	2.24

Compound	#	Calc	Exp
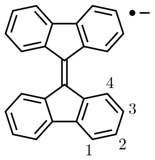	1	+2.4274	1.48
	2	−6.3617	5.42
	3	+1.4039	0.88
	4	−4.8007	4.34
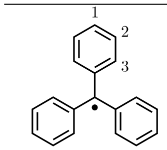	1	−9.8736	8.01
	2	+5.1674	3.20
	3	−9.1805	7.31
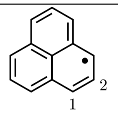	1	−21.7792	17.66
	2	+9.1583	5.04

Compound	#	Calc	Exp
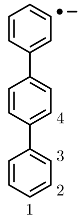	1	−12.4080	9.00
	2	+2.5598	1.54
	3	−7.3042	5.69
	4	−2.7363	2.63

Experimental constants were taken from literature for the naphthalene radical anion,[48] anthracene radical anion,[49] anthracenylfluorenyl radical,[50] trityl radical,[51] phenalenyl radical,[52] and terphenyl radical anion.[48] Values for the bifluorenylidene radical anion were taken from fitting our EPR data.

Table S18: Prediction of ^{1}H Hyperfine Coupling Constants (MHz)

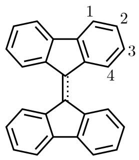

#	Calc	Pred[a]	Exp[b]
1	+2.4274	+1.2746	1.4770
2	−6.3617	−5.4012	5.4217
3	+1.4039	+0.4972	0.8778
4	−4.8007	−4.2155	4.3384

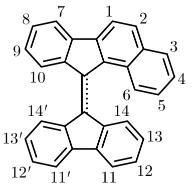

#	Calc	Pred[a]
1	+3.5045	+2.0927
2	−8.5537	−7.0661
3	−2.3928	−2.3866
4	+1.1757	+0.3238
5	−2.4104	−2.4000
6	+0.9811	+0.1760
7	+1.2492	+0.3797
8	−3.7054	−3.3836
9	−0.1453	−0.6795
10	−2.6000	−2.5440
11	+2.3227	+1.1950
11′	+2.6184	+1.4196
12	−6.1259	−5.2221
12′	−6.9156	−5.8219
13	+1.5102	+0.5779
13′	+1.8230	+0.8155
14	−5.2108	−4.5270
14′	−5.5636	−4.7950

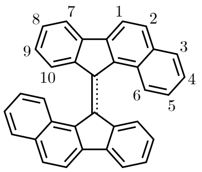

#	Calc	Pred[a]
1	+3.7274	+2.2619
2	−9.1044	−7.4844
3	−2.4715	−2.4464
4	+1.4239	+0.5123
5	−2.5899	−2.5363
6	+1.2985	+0.4171
7	+1.0733	+0.2460
8	−3.7222	−3.3964
9	−0.0196	−0.5840
10	−3.1447	−2.9577

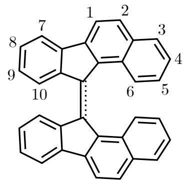

#	Calc	Pred[a]
1	+3.4823	+2.0758
2	−8.4147	−6.9605
3	−2.2704	−2.2936
4	+1.3247	+0.4370
5	−2.4076	−2.3979
6	+1.3442	+0.4518
7	+1.3969	+0.4918
8	−4.3706	−3.8888
9	+0.3675	−0.2900
10	−3.5478	−3.2639

[a] The 'predicted' values take the calculated A values and use the line of best fit $A_{\text{exp}} = 0.759547A_{\text{calc}} - 0.569173$ to estimate the experimental value. [b] Experimental hyperfine constants for $\mathbf{1}^{\bullet-}$ are only available in absolute value.

S5.2 CASSCF/RI-NEVPT2 Multireference Calculations

S5.2.1 CASSCF(n,2)/RI-NEVPT2 Dihedral Angle Excited State Scans

The potential energy surface scan was performed in two steps. Geometries across the potential energy surface were obtained through a nudged elastic band (NEB) calculation performed using ωB97X-D3/Def2-TZVPP DFT. Initial frames were calculated from ORCA's built-in IDPP (image-dependent pair potential) method by feeding in converged geometries for the starting point, transition state, and ending point. The jobs for the neutral species were queued up as broken-symmetry singlets: frames near the transition state seemed to retain this broken symmetry electronic configuration, and those nearer to the minima fell off this surface onto a typical singlet surface (as expected). The default NEB convergence criteria were used for all compounds except neutral **2** and neutral **3**, which gave quite slow NEB convergence. Because of the expense of NEB calculations, these two NEB runs were interrupted once the transition state was consistently the highest-energy frame over several iterations.

At each frame, CASSCF(n,2)/RI-NEVPT2 calculations ($n = 2$ for neutral, $n = 3$ for anion) were performed using the Def2-TZVPP basis set and CPCM(THF) solvation. For all compounds (regardless of charge), initial orbitals were quasi-restricted orbitals obtained through a BP/Def2-TZVPP + CPCM(THF) calculation on the neutral triplet species. This choice meant the π and π^* orbitals were expected to always be the HOMO and LUMO/SOMO of the system. The active space was chosen to be these π and π^* orbitals along with their 2–3 occupying electrons. State-averaged CASSCF calculations were performed including all valid configurations within these active spaces: for neutral species this meant the three singlets and one triplet, and for the anionic species this meant the two doublets. The energies across the potential energy surface are plotted in Fig. S26. The energies of the lowest energy singlet were used in the 'Hybrid' thermochemistry calculations described above (Section S5.1.1).

These multireference calculations on the neutral compounds accurately captured the energy of the $\pi \rightarrow \pi^*$ transition to be 22 080 cm^{-1} for **1**, 20 640 cm^{-1} for **2**, and 19 090 cm^{-1} for **3** (Fig. S26a,c,e). They showed that the energy of this transition should gradually fall in energy as the twist angle approaches 90° for **1** and **3**. It is also evident that the singlet ground state becomes nearly degenerate with the triplet configuration at the transition state. This is easiest to conceptualize by thinking about orbital occupancies in terms of the individual p orbitals on each carbon rather than the π/π^* interactions, which should be totally broken at 90°. The singlet and triplet levels of the transition state are nearly degenerate because they can both be considered to have $|(p_\mathrm{A})^1(p_\mathrm{B})^1\rangle$ electronic configurations (where the individual carbons are labeled 'A' and 'B'). At the transition state, the two higher energy singlet levels for **1** and **3** become nearly degenerate and the CASSCF wavefunctions suggest they should be thought of as having $|(p_\mathrm{A})^2(p_\mathrm{B})^0\rangle \pm |(p_\mathrm{A})^0(p_\mathrm{B})^2\rangle$ configurations. The behavior is slightly different in asymmetric **2**, where the electronic structures of the two highest singlet levels near 90° should be thought of as localized, $|(p_\mathrm{A})^2(p_\mathrm{B})^0\rangle$ and $|(p_\mathrm{A})^0(p_\mathrm{B})^2\rangle$, due to the different degrees of benzannulation on either half of the molecule. The predicted $\Delta E^\ddagger$ barriers from the CASSCF(2,2)/RI-NEVPT2 potential energy surfaces are also indicated. We have combined these with vibrational corrections from DFT calculations to obtain estimations of experimental barriers (Section S5.1.1).

Our computation results on the anions also accurately captured the $\pi \rightarrow \pi^*$ energies, which were found to vary strongly with twist angle across the central olefinic bond (Fig. S26b,d,f). The energy of the $\pi \rightarrow \pi^*$ transition of [**1**]$^{\bullet-}$ can be seen to monotonically decrease as the twist angle approaches 90°, at which point the two $\{\pi, \pi^*\}^3$ states become degenerate. Near 90° for the asymmetric [**2**]$^{\bullet-}$ species, the two surfaces do not approach each other as closely because the electron density becomes polarized towards the more delocalized side of the anion.

S5.2.2 Larger CASSCF(n,8)/RI-NEVPT2 Calculations

Larger active spaces for **1** and [**1**]$^{\bullet-}$ were also explored. The active spaces were carefully chosen so that the same states from TD-DFT calculations were also characterized. For the neutral species, this meant the HOMO (π) through HOMO–4 filled orbitals and the LUMO (π^*) through LUMO+2 empty orbitals, giving a CASSCF(10,8)/RI-NEVPT2 calculation that was run in a state-averaged manner over the lowest eight singlet roots and one triplet root. For the anionic species, this meant the HOMO–2 through the LUMO+4 orbitals, giving a CASSCF(7,8)/RI-NEVPT2 calculation that was run in a state-averaged manner over the lowest twelve doublet roots and two quartet roots. The results are summarized in Tables S19 and S20.

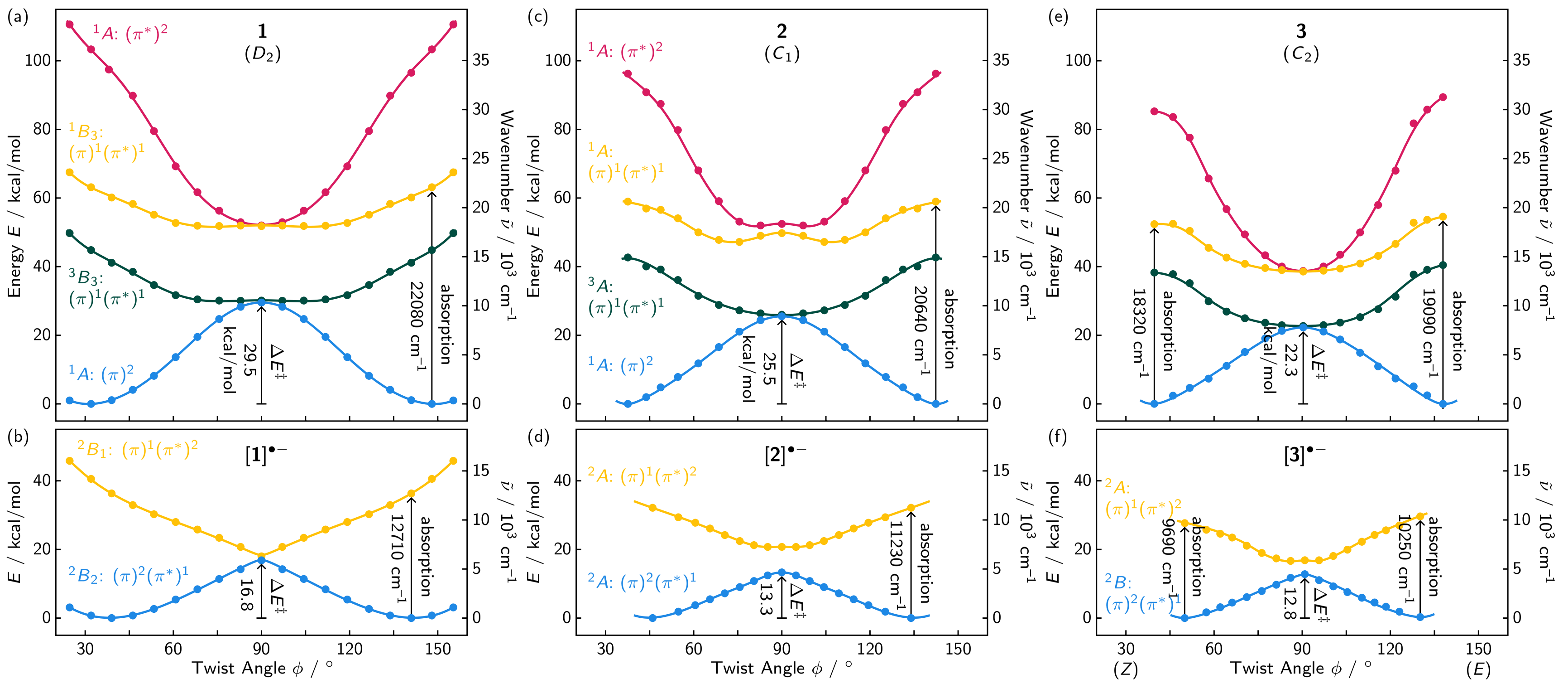

Figure S26: Potential energy surface scans using multireference CASSCF(n,2)/RI-NEVPT2 calculations were used to explore the energy of the $\pi \rightarrow \pi^*$ calculations as a function of twist angle ($n = 2$ for neutral species and $n = 3$ for anionic species).

Table S19: Calculated CASSCF(10,8)/RI-NEVPT2 transitions for **1**

$\tilde{\nu}$ / cm^{-1}	λ / nm	f_{osc}	D^2 / au^2	D_x / au	D_y / au	D_z / au
15871.4	630.1	*Triplet state*				
22449.6	445.4	0.709030009	10.39754	−3.22452	−0.00269	−0.00372
23136.2	432.2	0.003471690	0.04940	0.01844	−0.22134	0.00823
23244.6	430.2	0.000903104	0.01279	−0.01092	0.01631	0.11138
29190.4	342.6	0.011728656	0.13228	0.00515	−0.00003	−0.36366
29944.1	334.0	0.032024086	0.35208	−0.00092	−0.59336	0.00005
33705.9	296.7	0.116194766	1.13490	−1.06529	0.00705	0.00275
34183.3	292.5	0.000005809	0.00006	−0.00036	−0.00736	−0.00127

Table S20: Calculated CASSCF(7,8)/RI-NEVPT2 transitions for $[\mathbf{1}]^{\bullet-}$

$\tilde{\nu}$ / cm^{-1}	λ / nm	f_{osc}	D^2 / au^2	D_x / au	D_y / au	D_z / au
34185.0	292.5	*Quartet state*				
35141.1	284.6	*Quartet state*				
13097.0	763.5	0.359411363	9.03435	−3.00572	−0.00070	−0.00120
17792.1	562.0	0.000000039	0.00000	−0.00072	0.00039	−0.00023
18625.9	536.9	0.000381728	0.00675	−0.00131	0.00058	0.08213
18636.1	536.6	0.000352981	0.00624	0.00021	0.07896	−0.00056
18959.5	527.4	0.110840306	1.92463	1.38731	0.00013	0.00104
25847.2	386.9	0.100838724	1.28437	0.00045	−1.13330	0.00044
26495.3	377.4	0.021032512	0.26134	−0.00580	0.00078	0.51118
28840.1	346.7	0.076921647	0.87807	0.93705	0.00005	0.00094
30240.7	330.7	0.000001146	0.00001	−0.00022	0.00352	0.00008
33057.3	302.5	0.001257071	0.01252	−0.00009	0.11188	−0.00104
33100.0	302.1	0.000520226	0.00517	0.00010	0.00172	0.07191

S5.3 Discussion of Electronic Structure and the 3 × 3 CI Model

The electronic transitions in the visible/NIR energy range for the neutral and anionic bifluorenylidene species are primarily determined by the π systems of these systems. It is easiest to interpret the π system of **1** by considering it as a homodimer of fluorenylidene. We show in Fig. S27 the π system of the fluorenyl radical, which we use instead of fluorenylidene because their π systems should be identical but the fluorenyl radical should sidestep complications from the presence of a second unpaired electron in fluorenylidene. These orbitals were calculated using Hückel molecular orbital (HMO) theory, which assumes a Hamiltonian of $\mathcal{H} = \alpha I + \beta A$, where α is the energy of a carbon p_z orbital, I is the identity matrix, β is the interaction energy between two adjacent p_z orbitals, and A is the adjacency matrix describing connectivity within the π system. Despite its simplicity, it gives results that are easy to interpret and we found its prediction for the fluorenyl radical to match that of ωB97X-D3/Def2-TZVPP and SOS-PBE-QIDH/Def2-TZVPP DFT calculations fairly closely. The frontier orbitals of **1** are shown in Fig. S28 and can be constructed as in-phase/out-of-phase combinations of the fluorenyl MOs in Fig. S27. The HOMO and LUMO are largely localized on the central C=C moiety, so we will treat them as olefinic π/π^* MOs.

We can model the $\{\pi, \pi^*\}^2$ active space of the central olefinic moiety using a complete neglect of dif-

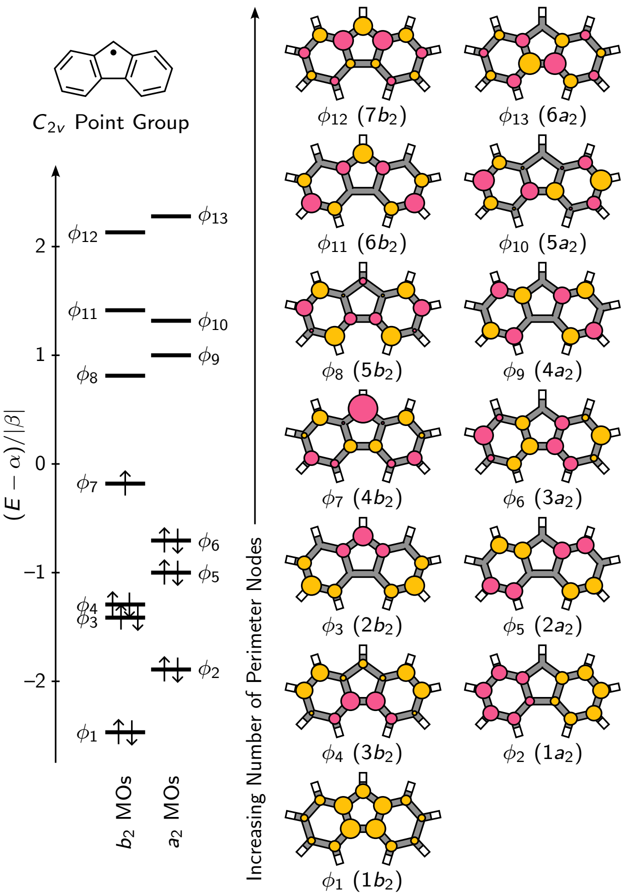

Figure S27: Analysis of the π system of a fluorenyl radical can be done using Hückel MO theory. (a) The MO energies are predicted based on two parameters: α is the energy of a carbon $2p$ orbital and β is the interaction energy between adjacent $2p$ orbitals. The left and right columns of MOs correspond to b_2 and a_2-symmetry orbitals. (b) Orbital illustrations show that the number of perimeter nodes generally increases with the energy of the MO. Non-alternant systems like this do not always have obvious patterns of perimeter nodes, so the vertical positioning in this grid can be ambiguous/qualitative (e.g. ϕ_3 vs ϕ_4). As in the energy diagram, the left and right columns indicate their C_{2v} irreducible representation.

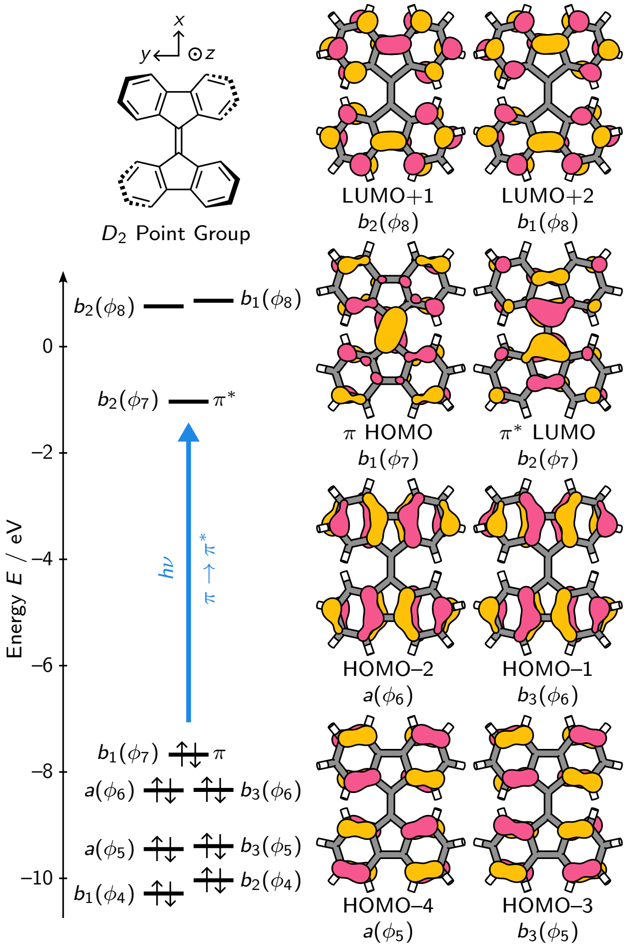

Figure S28: The HOMO and LUMO orbital wavefunctions of **1** have large coefficients on the central olefinic carbon centers. Other frontier orbitals from HOMO–4 to LUMO+2 are primarily found on the outer aromatic rings, and they appear as in-phase/out-of-phase pairs of MOs of the fluorenyl π system. The corresponding fluorenyl MO is indicated parenthetically according to the naming scheme in Fig. S27. Symmetry labels in the D_2 point group are attached, where the olefinic C=C is defined as the x axis, and the normal to the olefin plane is defined as the z axis.

ferential overlap (CNDO) approximation, which underlies the highly successful Pariser–Parr–Pople (PPP) method. We begin with the 3×3 CI model.[53] Here, we conceptualize the molecule as two electrons held within two p orbitals on adjacent carbon atoms, which we call A and B. The localization of the frontier orbitals in Fig. S28 justifies that this approximation can still provide insight into electronic structure. The 3×3 CI model uses a 6×6 Hamiltonian and it neglects spatial overlap between the two p orbitals ($S_{ij} = \delta_{ij}$). The Hamiltonian of the system is

$$\begin{matrix} |^1(A^2 - B^2)\rangle \\ |^1(A^2 + B^2)\rangle \\ |^1AB\rangle \\ |^3AB, M = +1\rangle \\ |^3AB, M = 0\rangle \\ |^3AB, M = -1\rangle \end{matrix} \begin{pmatrix} E_T + 2\bar{K} & \delta & \bar{\gamma} & 0 & 0 & 0 \\ \delta & E_T + 2(\bar{K} + K) & \gamma & 0 & 0 & 0 \\ \bar{\gamma} & \gamma & E_T + 2K & 0 & 0 & 0 \\ 0 & 0 & 0 & E_T & 0 & 0 \\ 0 & 0 & 0 & 0 & E_T & 0 \\ 0 & 0 & 0 & 0 & 0 & E_T \end{pmatrix} \tag{S13}$$

The variables within the matrix are composite quantities that relate to more fundamental one- and two-

electron integrals:

$$\begin{aligned} J_{AB} &= (AA|BB) \quad \text{and analogous for other } J\text{s} \\ K &= (AB|AB) \\ \gamma &= 2h_{AB} + (AA|AB) + (BB|BA) \\ \bar{\gamma} &= (AA|AB) - (BB|BA) \\ \delta &= h_{AA} - h_{BB} + (J_{AA} - J_{BB})/2 \\ \bar{K} &= [(J_{AA} + J_{BB})/2 - J_{AB}]/2 \\ E_T &= h_{AA} + h_{BB} + J_{AB} - K \end{aligned} \tag{S14}$$

The relevant one- and two-electron integrals are

$$S_{UV} = \int U(\vec{r})V(\vec{r})d^3\vec{r}, \quad \text{which we assume to equal } \delta_{UV} \tag{S15}$$

$$h_{UV} = \int U(\vec{r})\mathcal{H}V(\vec{r})d^3\vec{r} \tag{S16}$$

$$(UV|WX) = \iint U(\vec{r}_1)V(\vec{r}_1)\frac{e^2}{r_{12}}W(\vec{r}_2)X(\vec{r}_2)d^3\vec{r}_1 d^3\vec{r}_2 \tag{S17}$$

The quantities can be qualitatively understood as (all quoted passages are from Ref. 53):

- J_{AB} is the repulsion between one electron in A and the other in B
- K (called K_{AB} in Ref. 53) is the exchange integral: it "is the repulsion between the overlap charge density due to the first electron... and an identical charge density due to the second electron."
- γ (γ_{AB} in Ref. 53) "provides a measure of the interaction between A and B and corresponds roughly to twice the resonance integral of semiempirical theories".
- $\bar{\gamma}$ (γ^{-}_{AB} in Ref. 53) "is related to the degree of localization of orbitals A and B"
- δ (δ_{AB} in Ref. 53) "is equal to the energy difference between the two electron configurations A^2, in which both electrons reside in A, and the configuration B^2, in which both reside in B." The orbitals are chosen such that $\delta \geq 0$.
- E_T is the energy of the triplet state
- $\bar{K}$ (K'_{AB} in Ref. 53) is related to the energy difference in the absence of exchange between the A^1B^1 levels and the A^2/B^2 levels.

The values of J, K, and $\bar{K}$ should always be positive for real orbitals. However, the triplet should lie below the S_0 state by roughly $2K$ if we look at only the diagonal terms of the two-electron repulsion matrix; thus, any true molecule with a triplet lying above the singlet must have negative K or it must have strong enough off-diagonal elements (γ, $\bar{\gamma}$) to push the singlet level lower. Compounds **1** and **3** are 'homosymmetric biradicaloids' ($\gamma \neq 0, \delta = 0$) and **2** is a 'nonsymmetric biradicaloid' ($\gamma \neq 0, \delta \neq 0$).

We seek to model the potential energy surface (PES) as a function of twist angle, which we will call η here. We use the CNDO approximation to simplify the Hamiltonian so that it requires fitting fewer parameters to the PES; this approximation assumes all $(UV|WX)$ two-electron repulsion integrals are zero unless $U = V$ and $W = X$. This simplifies Eq. (S13) to

$$\begin{pmatrix} 2E_0 + \bar{J} & 2V + W & 0 & 0 & 0 & 0 \\ 2V + W & 2E_0 + \bar{J} & 2h_{AB} & 0 & 0 & 0 \\ 0 & 2h_{AB} & 2E_0 + J_{AB} & 0 & 0 & 0 \\ 0 & 0 & 0 & 2E_0 + J_{AB} & 0 & 0 \\ 0 & 0 & 0 & 0 & 2E_0 + J_{AB} & 0 \\ 0 & 0 & 0 & 0 & 0 & 2E_0 + J_{AB} \end{pmatrix} \tag{S18}$$

where $E_0 = (h_{AA} + h_{BB})/2$, $V = (h_{AA} - h_{BB})/2$ $\bar{J} = (J_{AA} + J_{BB})/2$, and $W = (J_{AA} - J_{BB})/2$. The one-electron energies can be easily described as a function of angle when we conceptualize the orbitals as two adjacent p orbitals,

$$\mathcal{H} = \begin{pmatrix} h_{AA} & h_{AB} \\ h_{AB} & h_{BB} \end{pmatrix} = \begin{pmatrix} E_0 + V & \frac{1}{2}\Delta E \cos\eta \\ \frac{1}{2}\Delta E \cos\eta & E_0 - V \end{pmatrix}, \tag{S19}$$

where h_{AA} and h_{BB} are the energies of the unmixed orbitals, and h_{AB} is their mixing energy. We assume h_{AB} varies as $\frac{1}{2}\Delta E \cos\eta$ (Fig. 1c in the main manuscript). In this way, the orbitals have maximal overlap when untwisted, and the mixing energy gradually decreases till 90°, when their crossed nodal planes prevent mixing between the p orbitals.

Variation of the two-electron integrals with twist angle are more difficult to model. The J_{AA} and J_{BB} values (and thus the composite $\bar{J}$ and W values) will not vary because these quantities describe electrons localized within a single p orbital, and thus cannot depend on the twist angle. In contrast, the J_{AB} value will depend on twist angle, and we can predict the dependence on η using the Neumann expansion of electrostatic potential in prolate spheroidal coordinates,[54]

$$r_{12}^{-1} = \sum_{\ell \geq 0} \sum_{m=-\ell}^{\ell} \frac{2}{R}(-1)^m (2\ell+1) \left[\frac{(\ell-|m|)!}{(\ell+|m|)!}\right]^2 \mathcal{P}_\ell^{|m|}(\sigma_<)\mathcal{Q}_\ell^{|m|}(\sigma_>)P_\ell^{|m|}(\tau_1)P_\ell^{|m|}(\tau_2)e^{im\phi_1}e^{-im\phi_2} \tag{S20}$$

where $\mathcal{P}/\mathcal{Q}$ are the associated Legendre functions, P/Q are the associated Legendre polynomials, and R is the internuclear distance (distance between foci of the prolate spheroidal coordinate system). It is clear that each term in this infinite series is just a component varying in $\sigma_1, \tau_1, \sigma_2, \tau_2$ variables and a component varying in the azimuthal ϕ_1, ϕ_2 variables. We can use that to our advantage if we recognize that two p orbitals twisting along the internuclear axis can also be written as products of a function in σ, τ and an azimuthal function in ϕ:

$$\begin{aligned} &p_A(r,\theta,\phi) = \mathrm{R}(r)\Theta(\theta)\cos\phi \text{ in spherical coordinates} \\ &\quad \rightarrow \quad p_A(\sigma,\tau,\phi) = \mathrm{R}\left(\frac{R}{2}(\sigma-\tau)\right)\Theta\left(\cos^{-1}\left(\frac{\sigma\tau-1}{\sigma-\tau}\right)\right)\cos\phi && \text{(S21)} \\ &p_B(r,\theta,\phi) = \mathrm{R}(r)\Theta(\theta)\cos(\phi+\eta) \text{ in spherical coordinates} \\ &\quad \rightarrow \quad p_B(\sigma,\tau,\phi) = \mathrm{R}\left(\frac{R}{2}(\sigma+\tau)\right)\Theta\left(\cos^{-1}\left(\frac{\sigma\tau+1}{\sigma+\tau}\right)\right)\cos(\phi+\eta) && \text{(S22)} \end{aligned}$$

Here, these orbitals are now centered on the foci of the prolate spheroidal coordinate systems. We have assumed p_A is directed exactly along the x axis and p_B is twisted relative to it by an angle η. Because $\cos\phi = (e^{i\phi} + e^{-i\phi})/2$, terms in Eq. (S20) can only be nonzero if $m = 0, \pm 2$. We can rewrite the Neumann expansion and introduce an abbreviation F as

$$\begin{aligned} r_{12}^{-1} &= \sum_{m=0,\pm 2} (-1)^m \underbrace{\sum_{\ell=|m|}^{\infty} \frac{2}{R}(-1)^m(2\ell+1)\left[\frac{(\ell-|m|)!}{(\ell+|m|)!}\right]^2 \mathcal{P}_\ell^{|m|}(\sigma_<)\mathcal{Q}_\ell^{|m|}(\sigma_>)P_\ell^{|m|}(\tau_1)P_\ell^{|m|}(\tau_2)}_{F_\ell^{|m|}(\sigma_1,\tau_1,\sigma_2,\tau_2)} e^{im\phi_1}e^{-im\phi_2} \\ &= \sum_{m=0,\pm 2} (-1)^m F_\ell^{|m|}(\sigma_1,\tau_1,\sigma_2,\tau_2)e^{im\phi_1}e^{-im\phi_2} \end{aligned} \tag{S23}$$

This can be used to evaluate the dependence on twist angle, giving

$$\begin{aligned}
J_{AB} = (AA|BB) &= \int [p_A(\vec{r}_1)^* p_A(\vec{r}_1)] r_{12}^{-1} [p_B(\vec{r}_2)^* p_B(\vec{r}_2)] d^3\vec{r}_1 d^3\vec{r}_2 \\
&= \left(\frac{1}{4\pi}\frac{R^3}{8}\right)^2 \int [f_A(\sigma_1,\tau_1)\cos\phi_1]^2 r_{12}^{-1} [f_B(\sigma_2,\tau_2)\cos(\phi_2+\eta)]^2 (\sigma_1^2-\tau_1^2)(\sigma_2^2-\tau_2^2) d\sigma_1 d\sigma_2 d\tau_1 d\tau_2 d\phi_1 d\phi_2 \\
&= \sum_{m=0,\pm 2} (-1)^m \left\{ \left(\frac{1}{2}\frac{R^3}{8}\right)^2 \int f_A(\sigma_1,\tau_1)^2 F_\ell^{|m|}(\sigma_1,\tau_1,\sigma_2,\tau_2) f_B(\sigma_2,\tau_2)^2 (\sigma_1^2-\tau_1^2)(\sigma_2^2-\tau_2^2) d\sigma_1 d\sigma_2 d\tau_1 d\tau_2 \right\} \\
&\qquad \times \left\{ \left(\frac{1}{2\pi}\right)^2 \int \cos^2\phi_1 e^{im(\phi_1-\phi_2)} \cos^2(\phi_2+\eta) d\phi_1 d\phi_2 \right\} \\
&= \underbrace{\begin{Bmatrix} m=0 \\ \sigma\tau \text{ integral}\end{Bmatrix} \left(\frac{\cos^2\eta}{4}\right)}_{Y\cos^2\eta} + \underbrace{\begin{Bmatrix} m=+2 \\ \sigma\tau \text{ integral}\end{Bmatrix} \left(\frac{1}{16}\right) + \begin{Bmatrix} m=-2 \\ \sigma\tau \text{ integral}\end{Bmatrix} \left(\frac{1}{16}\right)}_{X}
\end{aligned} \tag{S24}$$

Here, we abbreviate $f_A(\sigma,\tau) = \mathrm{R}\left(\frac{R}{2}(\sigma-\tau)\right)\Theta\left(\cos^{-1}\left(\frac{\sigma\tau-1}{\sigma-\tau}\right)\right)$ so that $p_A(\sigma,\tau,\phi) = f_A(\sigma,\tau)\cos\phi$, and analogously $f_B(\sigma,\tau) = \mathrm{R}\left(\frac{R}{2}(\sigma+\tau)\right)\Theta\left(\cos^{-1}\left(\frac{\sigma\tau+1}{\sigma+\tau}\right)\right)$ so that $p_B(\sigma,\tau,\phi) = f_B(\sigma,\tau)\cos(\phi+\eta)$. We can clearly see that the rigid twisting of two p orbitals causes J_{AB} to vary as $X + Y\cos^2\eta$ for two constants X and Y, which we treat as adjustable parameters to fit to the calculated PES. The final η-dependent Hamiltonian for this two-electron $\{\pi,\pi^*\}^2$ system is

$$\begin{pmatrix}
2E_0+\bar{J} & 2V+W & 0 & 0 & 0 & 0 \\
2V+W & 2E_0+\bar{J} & \Delta E\cos\eta & 0 & 0 & 0 \\
0 & \Delta E\cos\eta & 2E_0+X+Y\cos^2\eta & 0 & 0 & 0 \\
0 & 0 & 0 & 2E_0+X+Y\cos^2\eta & 0 & 0 \\
0 & 0 & 0 & 0 & 2E_0+X+Y\cos^2\eta & 0 \\
0 & 0 & 0 & 0 & 0 & 2E_0+X+Y\cos^2\eta
\end{pmatrix} \tag{S25}$$

The final η-dependent Hamiltonian for the three-electron $\{\pi,\pi^*\}^3$ system is simpler and can be modeled with Eq. (S19).

The PESs shown in Fig. S26 cannot be directly fit because Eqs. (S25) and (S19) do not incorporate any terms to capture the influence of steric clashing. Instead, we can fit the traceless parts of Eqs. (S25) and (S19) to the deviations between the PES from the energetic barycenter of the active space. These fits are shown in Fig. S29. The fitted ΔE values can be used as a way to interrogate how delocalization impacts the strength of the π bond. All ΔE values among the neutral compounds are quite similar, and the ΔE values among the anions are also quite similar. This shows that the increase in twist angle is primarily driven by increases in steric clashing rather than a weakening of the central C=C bond.

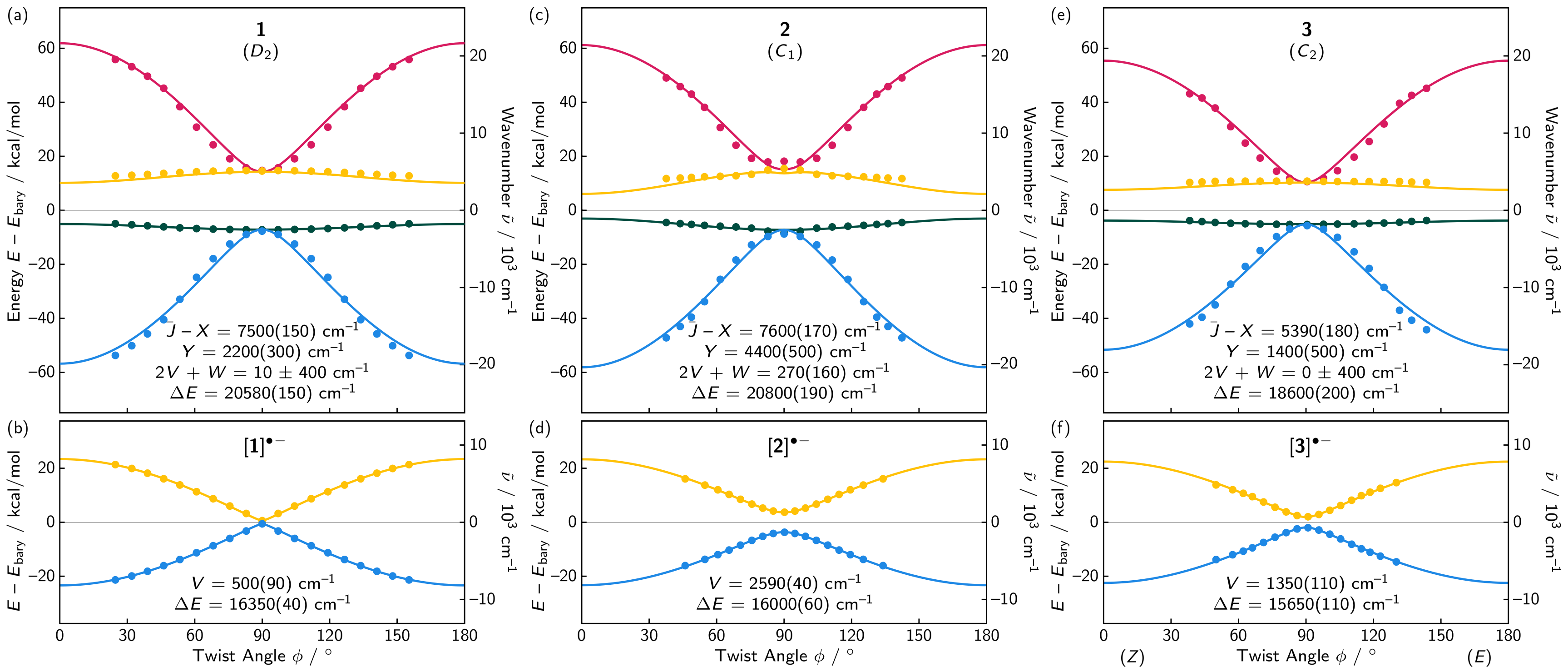

Figure S29: Best fits of the CASSCF(n,2)/RI-NEVPT2 PESs using Eqs. (S25) and (S19).

S5.4 XYZ Coordinates

S5.4.1 Neutral and Anion 1

Neutral 1

C	0.67936049682489	-0.00026942924281	-0.00891443204172
C	1.58207427929395	-1.13168283780199	0.31627019234418
C	1.32015813549439	-2.37923886608508	0.87116816429499
H	0.30783749564135	-2.71082569583223	1.05223182513783
C	2.37970060428292	-3.20562736067216	1.21987215703241
H	2.17595491909494	-4.18283529305696	1.63916297785495
C	3.69620756002702	-2.78924977787734	1.05082905561640
H	4.50901229772329	-3.44872936869295	1.32808302317710
C	3.97095197557625	-1.51979402294116	0.56138783903069
H	4.99347804444582	-1.17266425859255	0.47622458164274
C	2.91308794590064	-0.69775293062545	0.21149255776915
C	2.91254960427003	0.69666225049460	-0.23567857437509
C	3.97005613145033	1.51813224648253	-0.58786252169074
H	4.99239914475983	1.16955583323262	-0.50645422528065
C	3.69518623902904	2.78869223911407	-1.07428644126488
H	4.50761340590794	3.44794976595567	-1.35314958771957
C	2.37855226684322	3.20662413276870	-1.23777070643382
H	2.17396734871719	4.18488585649068	-1.65419122670191
C	1.31922348262084	2.38078268404992	-0.88689979262827
H	0.30699054037817	2.71487280681409	-1.06353049244675
C	1.58128681412845	1.13172112164272	-0.33549955098116
C	-0.67882913802941	-0.00151236992946	-0.00981156597367
C	-1.58228831539276	1.12945365742897	0.31465647845994
C	-1.32151253434832	2.37726555156880	0.86945198884019
H	-0.30959363311629	2.70959053492098	1.05130993111796
C	-2.38181236858200	3.20323543047284	1.21712678430990
H	-2.17854372951829	4.18063749212137	1.63619641922103
C	-3.69793125108414	2.78613465974502	1.04727376638662
H	-4.51119800541636	3.44525380829924	1.32402758670218
C	-3.97157881613016	1.51643783974473	0.55782651437920
H	-4.99380660698947	1.16855665009907	0.47211360401200
C	-2.91307018138395	0.69491023840751	0.20877719721021
C	-2.91158159236412	-0.69945865570941	-0.23854700082310
C	-3.96828670360726	-1.52151690717076	-0.59184047354537
H	-4.99097534792587	-1.17372649961876	-0.51141966831890
C	-3.69220239911784	-2.79180198161031	-1.07824487858898
H	-4.50403809256101	-3.45142088847286	-1.35797835588215
C	-2.37519285258076	-3.20905227946506	-1.24069349379300
H	-2.16986458702782	-4.18719238258905	-1.65703151440478
C	-1.31668728750923	-2.38264408961410	-0.88880720979108
H	-0.30397915477051	-2.71580516462998	-1.06449308584951
C	-1.58006613495492	-1.13384373962368	-0.33737784600456

Neutral 1 Transition State

C	0.72785801285011	-0.00224928241721	0.00930033921236
C	1.57780038091786	-0.82161472013413	0.82834639952104
C	1.26137366765276	-1.78149180811002	1.78836171863049
H	0.22783656997734	-2.01174299250480	2.01877888904700
C	2.29784568578030	-2.43386360449819	2.44071440922290

H	2.07396599109587	-3.18286999213634	3.18988672276563
C	3.62681041300476	-2.13661895725158	2.14332432919415
H	4.41977291236491	-2.65798789693843	2.66477925481815
C	3.95201142001426	-1.17741069408126	1.18394863327496
H	4.98934547431950	-0.95629819106458	0.96279168830923
C	2.92980239285503	-0.52210547270193	0.52861595428083
C	2.93036563934641	0.51593129301650	-0.50936508918700
C	3.95326883071494	1.17047420386293	-1.16437485768417
H	4.99037624907411	0.94854475546183	-0.94298349173063
C	3.62908713636989	2.13002668003633	-2.12376303718304
H	4.42260686872590	2.65079408711976	-2.64496803970407
C	2.30043539686791	2.42834090456517	-2.42149729609568
H	2.07731527359297	3.17764150066918	-3.17060685614915
C	1.26328611122721	1.77669087158416	-1.76950839709860
H	0.22999736441096	2.00782076185477	-2.00016086268704
C	1.57868394956645	0.81648186680892	-0.80948374507204
C	-0.72812818470697	-0.00180052492461	0.00894103515175
C	-1.57859267343677	0.81717607349227	0.82784121278394
C	-1.26279721412551	1.77697899334290	1.78814657765548
H	-0.22941048015060	2.00736788880848	2.01910469575135
C	-2.29968626149832	2.42907819977374	2.44010114986402
H	-2.07627955940593	3.17803910526520	3.18945955817836
C	-3.62846421928363	2.13167995486491	2.14203189219578
H	-4.42175034763258	2.65292632189475	2.66311730708064
C	-3.95304461615987	1.17250345642914	1.18240391072644
H	-4.99023415958037	0.95129490104349	0.96067654335760
C	-2.93041387073161	0.51741077310009	0.52750030657544
C	-2.93030659644808	-0.52055584607512	-0.51057855130469
C	-3.95275930327990	-1.17555008939719	-1.16586097239366
H	-4.99001807883810	-0.95405772955687	-0.94472796922041
C	-3.62792553347318	-2.13498327844459	-2.12513849438641
H	-4.42108837207325	-2.65613590706537	-2.64650331719666
C	-2.29907290217278	-2.43276383462021	-2.42249179069724
H	-2.07546576005576	-3.18193286797523	-3.17158481795375
C	-1.26237069047215	-1.78073404285847	-1.77018499885419
H	-0.22892231118513	-2.01143050958963	-2.00054728786268
C	-1.57842460601892	-0.82066435064876	-0.81024265513642

Anion 1

C	0.70932586149271	0.00086766314058	-0.00472071620844
C	1.58429725203596	-1.08102442404328	0.39657962590097
C	1.31542644115018	-2.33127426317224	0.97434724899280
H	0.29480686878763	-2.64842659868535	1.14742151315075
C	2.36263150680997	-3.15765204356559	1.33762588947873
H	2.14939986702078	-4.12502927665315	1.77776290548148
C	3.69461080879929	-2.76396894780298	1.15668523822049
H	4.49793781844204	-3.42995369373316	1.44749886568774
C	3.98237245143873	-1.51670137813949	0.62641556685915
H	5.01229989165904	-1.19581681952744	0.51308380604632
C	2.93815213921664	-0.67674712655297	0.25557610362346
C	2.93872823606943	0.67710379267370	-0.26382682062516
C	3.98394971075301	1.51627305736772	-0.63360867392685
H	5.01345216303636	1.19464244200589	-0.51849270751467
C	3.69774089534306	2.76311344053641	-1.16561960548908

H	4.50169809699494	3.42859154617121	-1.45584451205994
C	2.36623968912272	3.15682314297082	-1.34982939773969
H	2.15379842541105	4.12364537431869	-1.79155969725326
C	1.31801958152347	2.33123145937551	-0.98762664238817
H	0.29805180803133	2.64873415589594	-1.16339706370817
C	1.58516000854995	1.08178498303808	-0.40727235675697
C	-0.70865593182097	0.00058976246872	-0.00535931908527
C	-1.58506363539542	1.08105208392592	0.39695401375115
C	-1.31845700707813	2.33040702154513	0.97776084706527
H	-0.29863099591428	2.64798641576129	1.15422486001923
C	-2.36705551273436	3.15581649286568	1.33932846494173
H	-2.15497507464645	4.12254969880880	1.78143713166154
C	-3.69834899616083	2.76194960333071	1.15400360370954
H	-4.50262079799437	3.42724606760869	1.44378253946803
C	-3.98403188264879	1.51517043748408	0.62157344120792
H	-5.01340193019265	1.19339818894036	0.50567363957472
C	-2.93843596193644	0.67613915523111	0.25246083676837
C	-2.93715385604231	-0.67777200085389	-0.26675186894025
C	-3.98089900280533	-1.51829842024119	-0.63768061988245
H	-5.01097436492239	-1.19766013700397	-0.52496639649786
C	-3.69250020196365	-2.76566992131196	-1.16729533885197
H	-4.49538636097053	-3.43213676121541	-1.45822278148855
C	-2.36031240479556	-3.15895447389422	-1.34758164557781
H	-2.14649955558891	-4.12642145811461	-1.78723390772714
C	-1.31355169341651	-2.33204613202761	-0.98421570048845
H	-0.29274502918893	-2.64890588260692	-1.15667179666988
C	-1.58308932547149	-1.08167622631962	-0.40702857272937

Anion 1 Transition State

C	0.73047648560052	0.01063816316808	-0.01528049221605
C	1.59716007018679	-0.80474259305543	0.79823006004612
C	1.28612097333574	-1.76679660304599	1.75790463786374
H	0.25070897853995	-1.99374483665556	1.98424154564115
C	2.32174132095935	-2.42067977018117	2.41032451855546
H	2.09771615683616	-3.17096685693269	3.15880663041259
C	3.65219145157085	-2.12363099073820	2.11412991641721
H	4.44548800088282	-2.64565170642834	2.63506025028395
C	3.97490046557591	-1.16318296955904	1.15601695693333
H	5.01232059226596	-0.94084171531167	0.93427829269959
C	2.95035163992508	-0.50733704489975	0.50162173876064
C	2.94904356976461	0.53151696349510	-0.53481460513846
C	3.97194828478244	1.18872113897848	-1.19042067366319
H	5.00992218555791	0.96779493482313	-0.96987143701291
C	3.64681527473193	2.14869690806710	-2.14818355918487
H	4.43881572842698	2.67174665069647	-2.67005187846423
C	2.31561978838551	2.44393253175844	-2.44283822834065
H	2.08973236573431	3.19388460594628	-3.19109649085936
C	1.28163438922825	1.78869269288440	-1.78917793243898
H	0.24564520520687	2.01422430347884	-2.01427974258632
C	1.59512547283272	0.82710892265394	-0.82983472994869
C	-0.73011779445075	0.00981435635009	-0.01448291010201
C	-1.57261732199861	0.81384045721179	0.79200134461638
C	-1.28799069923315	1.79177654509448	1.77190881187709
H	-0.25838417119809	2.03527524878011	2.01524024582780

C	-2.32266387278171	2.43341569739687	2.41561511281318
H	-2.10018939705331	3.18349583330178	3.16708126418997
C	-3.66895020241800	2.13647218378540	2.11895798180080
H	-4.46291376445036	2.65701917762106	2.64120531547311
C	-3.97297770263764	1.18404805769603	1.16460115454486
H	-5.01013284207877	0.95655419732529	0.93729869724369
C	-2.94538597488761	0.51567165348056	0.49401671846392
C	-2.94528373826649	-0.49767778079473	-0.52183904143787
C	-3.97272286051129	-1.16674280968138	-1.19198172464230
H	-5.00993149779995	-0.94000736659501	-0.96414826371931
C	-3.66844601859382	-2.11880635580556	-2.14661923678696
H	-4.46227032146109	-2.63985561733085	-2.66856993218887
C	-2.32208947768933	-2.41470093727098	-2.44398836047504
H	-2.09945240087994	-3.16449120886887	-3.19568725678701
C	-1.28757238113565	-1.77240484828105	-1.80069932469622
H	-0.25790915797690	-2.01516170915069	-2.04454982933035
C	-1.57245680282822	-0.79481750340675	-0.82053554444493

S5.4.2 Neutral and Anion 2

Neutral 2

C	0.67966635991351	0.01238764849370	0.03189230497819
C	1.60037295622591	-1.11192577581816	0.29481339472451
C	1.41812581640862	-2.31660956567063	1.03899418613094
C	0.24384931128174	-2.64605144180968	1.75987316893641
H	-0.57833319211561	-1.94636993884959	1.78516569917032
C	0.14089764802009	-3.82406460562751	2.44431018973841
H	-0.76388320628528	-4.04531518215027	2.99658937349014
C	1.20581116023053	-4.74812911417032	2.45022337558052
H	1.10583258711294	-5.68514106418065	2.98314944491489
C	2.37164908987014	-4.43756660730304	1.81294819264222
H	3.21499030599596	-5.11756235891047	1.84434491073411
C	2.52143205891410	-3.20954130884097	1.12235588620036
C	3.77759598498722	-2.84981410353764	0.56932904639014
H	4.59711209233856	-3.55400153171133	0.65053950710125
C	3.97189346609943	-1.62211987320156	0.00694347858250
H	4.95096084051641	-1.32204899213755	-0.34487610744619
C	2.87658007003566	-0.75030351264157	-0.10461246946760
C	2.85333104091033	0.63484818349635	-0.57155067642904
C	3.86906496822625	1.40166678703151	-1.11596939009530
H	4.86721555702898	0.99651196089241	-1.22950258083288
C	3.58093743468654	2.69459616503475	-1.53129501407427
H	4.36234226600496	3.31490126583751	-1.95189641373704
C	2.28446891099050	3.18486553501385	-1.43225710065360
H	2.06018728143745	4.18514282322294	-1.78085324084771
C	1.26246273562324	2.40869101449300	-0.89880726376317
H	0.26126299598576	2.81094480355674	-0.87517528970582
C	1.54868947095236	1.13571041596215	-0.42139613528161
C	-0.68063167552080	-0.01239819244246	-0.02076557916892
C	-1.61374048021636	1.10629495256811	0.23395755447840
C	-1.40762746144057	2.33246467033952	0.85402663990490
H	-0.41895603252864	2.64334797754767	1.16253549732729
C	-2.49575305954383	3.15785413411901	1.10440313902554
H	-2.33815503750157	4.11999984459332	1.57510862758748

C	-3.78553547623463	2.75572872342843	0.77340737567294
H	-4.62163207095642	3.41250443915363	0.97844334949898
C	-4.01070357883995	1.50243313445288	0.21827940253354
H	-5.01934456099959	1.16838323925318	0.00775276764881
C	-2.92493465470750	0.68184580687147	-0.03312449792437
C	-2.87276648902923	-0.70430190128465	-0.50599468107004
C	-3.88029366332855	-1.52337248699363	-0.98669560426994
H	-4.90578482655476	-1.17651670318890	-1.02318911261524
C	-3.54835829674579	-2.79272696749932	-1.44112315289466
H	-4.32174061436032	-3.45099437007076	-1.81661191869779
C	-2.22294518792838	-3.21507179173596	-1.44099624430910
H	-1.97218842983800	-4.19695764808792	-1.82204743633191
C	-1.21213459803908	-2.39157385210528	-0.96482622766937
H	-0.18735359221317	-2.73266265990928	-0.99914869761687
C	-1.53939622486916	-1.14097197548300	-0.45502167809036

Neutral 2 Transition State

C	0.74598351883621	0.15751345450843	0.11333006346160
C	1.62756704190383	-0.62328274470423	0.90705388879916
C	1.37076634803342	-1.60439258007265	1.92187619703097
C	0.07993293290855	-1.99238601127384	2.33777823929349
H	-0.78948720409165	-1.53859269344427	1.88195794788565
C	-0.09132636083647	-2.93952531884709	3.31443298170957
H	-1.09194079417702	-3.22211686459883	3.61690096107840
C	1.02004366577632	-3.54649329710286	3.92469760014117
H	0.87212462242800	-4.29382845507263	4.69398878202117
C	2.28471313399146	-3.18957690795794	3.54184990084951
H	3.14945512592327	-3.65053894820803	4.00490904236740
C	2.49357017007868	-2.21660713842721	2.53831774718866
C	3.81489072617595	-1.85336623401294	2.14811742199021
H	4.64703678172948	-2.34183642065298	2.64105668432628
C	4.04606000099493	-0.91325340474129	1.17636501829691
H	5.05916130995236	-0.65212109683941	0.89586423206409
C	2.95640496186719	-0.29906628950470	0.55596488780165
C	2.91451096363598	0.71880542091958	-0.49439846755590
C	3.90621691791413	1.37983040476247	-1.19016836929934
H	4.95374904295262	1.18106955817176	-0.99788381044795
C	3.53285320471647	2.31718358465129	-2.15455000086064
H	4.29895248117333	2.84496479476219	-2.70869866731740
C	2.19209717556238	2.58358622958050	-2.41342963855173
H	1.92898287119293	3.31548653447359	-3.16674605262637
C	1.18694489197964	1.92121573233363	-1.71595277729337
H	0.14271774584244	2.12895561187121	-1.91835337624627
C	1.55443662738165	0.98846990599461	-0.75556099192262
C	-0.71123825335583	0.17485960201354	0.11724756357313
C	-1.54842463303212	1.02518534709445	0.91800731315863
C	-1.21635449572511	1.99115557853422	1.86620165275811
H	-0.17925680675488	2.20664638120181	2.09475426352968
C	-2.24243707081164	2.66802632983998	2.50969511460844
H	-2.00692456031879	3.42232380525316	3.24991249691162
C	-3.57593805312743	2.38758114850886	2.21602211244974
H	-4.36052597710978	2.92687747328096	2.73187768206413
C	-3.91639510556909	1.42115080783495	1.26964715577258
H	-4.95701890364592	1.21185478009552	1.05270326017278

C	-2.90439293218959	0.74184346981133	0.62239567568440
C	-2.92093585049487	-0.31327064561446	-0.39790320085964
C	-3.95380859109595	-0.96591356009159	-1.03952189747797
H	-4.98744512271962	-0.72583981630199	-0.82092385287791
C	-3.64416168968479	-1.94896423195973	-1.97938644702482
H	-4.44542773169531	-2.46847140243573	-2.48987788389501
C	-2.32013609529308	-2.27313519142366	-2.27108712360553
H	-2.10850886630946	-3.04047013612025	-3.00509671827588
C	-1.27325840860389	-1.62474212055939	-1.63178077851830
H	-0.24342574477212	-1.87638304927846	-1.85621481689530
C	-1.57440301153683	-0.63991139625190	-0.69270101543721

Anion 2

C	0.71325417206615	-0.03579055490491	-0.04430528470958
C	1.62831830460041	-1.08608145281588	0.32589754606035
C	1.44264976377747	-2.31291723365077	1.05678305540565
C	0.21905044519913	-2.70024833843032	1.64886459026915
H	-0.63289884324807	-2.04130246849383	1.56668737860634
C	0.09297381978964	-3.88125707392104	2.33402057609985
H	-0.85894122496817	-4.14605744206012	2.77822386494024
C	1.19278203684567	-4.74651288636663	2.46529809354725
H	1.08658660732588	-5.68537428635315	2.99510921063293
C	2.40400212792517	-4.38156121017343	1.94181863089836
H	3.26843082296291	-5.02505989948134	2.06428846704762
C	2.57015458251960	-3.15800139324471	1.25270827213621
C	3.86309807947802	-2.75805852280525	0.80072029513698
H	4.69913328197732	-3.42736263196896	0.96744858372902
C	4.04808046499005	-1.53893142649669	0.21733230504981
H	5.04150129731944	-1.21842342736155	-0.07735912423935
C	2.94350616235791	-0.69831952945323	-0.00759608579561
C	2.89409208102850	0.62938097658024	-0.56805608946183
C	3.89426339541131	1.45117079907548	-1.08502916637031
H	4.92936218899083	1.12686041527068	-1.08053972782518
C	3.55141515842244	2.68128841505209	-1.61710040849247
H	4.31823178079104	3.33382623764723	-2.01704163157817
C	2.20802938260671	3.07953635374491	-1.65781917124992
H	1.94841519020204	4.03562413338284	-2.09754933534331
C	1.20657607445351	2.26991043602344	-1.15527654141769
H	0.17476727896262	2.59062632236075	-1.22081675441084
C	1.53708850922570	1.04012354971315	-0.56881347155889
C	-0.70810573290191	-0.04246384650516	-0.06971677180513
C	-1.59326921466729	1.04033503103194	0.30365044296300
C	-1.34091706897478	2.26625358655412	0.93414708768544
H	-0.32692099827411	2.55704860828105	1.18020952669223
C	-2.39731652080525	3.10126918328848	1.25039686126821
H	-2.20045957022296	4.05192939536396	1.73226295263492
C	-3.71861367844408	2.73599828427279	0.96694338508784
H	-4.52919209936691	3.40715767949095	1.22361673606946
C	-3.99115816004012	1.51106414196283	0.37676796035342
H	-5.01605314209883	1.21606652520501	0.17969412573050
C	-2.93835131689156	0.66447672463146	0.05292210731036
C	-2.92005565294430	-0.67364258312050	-0.51361618086836
C	-3.94355750979075	-1.48875048172199	-0.98107906187641
H	-4.97525810375338	-1.15731558847525	-0.93651083930873

C	-3.63003362069545	-2.72828962092330	-1.51810921182050
H	-4.41732256935017	-3.37662590922744	-1.88311792536433
C	-2.29522548455002	-3.14044226517494	-1.60322476833399
H	-2.06291599408364	-4.10509545472248	-2.03930255186700
C	-1.26738939488880	-2.33625849517542	-1.14447936834552
H	-0.23974136624399	-2.66781880189586	-1.22819650330333
C	-1.56752574202496	-1.09497397400923	-0.56950608000869

Anion 2 Transition State

C	0.74645879806611	0.16094521806367	0.11014320719267
C	1.62830424879603	-0.61213044784289	0.89830823624905
C	1.39072738069369	-1.60021093766476	1.92354407564339
C	0.10042247238723	-1.98809127360179	2.34214831343358
H	-0.76391677883419	-1.52872413401136	1.87960165621573
C	-0.08794939532521	-2.93354663255264	3.32094345849726
H	-1.09216619756048	-3.20984603226936	3.61906395287411
C	1.01998944622895	-3.54051650987974	3.93224107170751
H	0.87331252104261	-4.28710091447921	4.70370777100338
C	2.28786603945396	-3.18420758371621	3.54701850455279
H	3.14858600417042	-3.64976905406629	4.01559669808329
C	2.51220656984878	-2.21553307226356	2.54406033617918
C	3.84484386753527	-1.85870930046981	2.15747861968672
H	4.67576710214619	-2.34818014758414	2.65242225472259
C	4.06041070413068	-0.92383998069455	1.18764234241459
H	5.07608607640169	-0.66331629607310	0.90557163734257
C	2.97487498734975	-0.29189457843330	0.54832461390259
C	2.93055548174861	0.69867406196944	-0.47809421287541
C	3.92508297516880	1.37738800154673	-1.19515343397464
H	4.97385076507118	1.17568288456793	-1.00018910669401
C	3.56716704291090	2.30510420665641	-2.15174507731943
H	4.33280623107735	2.83302097144760	-2.70826048910215
C	2.20622314575066	2.57395139540513	-2.41151786850802
H	1.94609403074889	3.30696863128043	-3.16724008197034
C	1.20788782543461	1.92253720793925	-1.72228126246628
H	0.16545180304758	2.14019148377544	-1.93364603788122
C	1.54855872720394	0.96987467668332	-0.73943043503075
C	-0.71495066103223	0.17800958805289	0.11395649123514
C	-1.56688793988586	1.02575900604734	0.90879952336845
C	-1.23930770128374	1.99578912339892	1.85449216726176
H	-0.20044994131958	2.20884126318012	2.07818435442259
C	-2.26433618877535	2.67509561609505	2.49734421840001
H	-2.02833689647877	3.43198983769475	3.23534564222291
C	-3.59937332959399	2.39417158689157	2.20613399146722
H	-4.38403452270024	2.93490589968894	2.72097498936312
C	-3.93793470670657	1.42470833692021	1.26303035913596
H	-4.97874799717496	1.21365508150837	1.04654323282069
C	-2.92405692673179	0.74375569903718	0.61737323102212
C	-2.93940299301482	-0.31374871557222	-0.39975811599923
C	-3.97259200307406	-0.96946654532093	-1.04085932171131
H	-5.00695385320365	-0.72971535101513	-0.82323830387276
C	-3.66250545280815	-1.95413158422337	-1.97804188601619
H	-4.46260660795459	-2.47613669896349	-2.48840905061551
C	-2.33620955968238	-2.27612564085835	-2.26672273789802
H	-2.12224649175307	-3.04511894238234	-2.99892361077739

C	-1.29193480863004	-1.62360365182511	-1.62712924324364
H	-0.25974690761538	-1.87061995718424	-1.84673350657503
C	-1.59088638527478	-0.63623579490277	-0.68993116788967

S5.4.3 Neutral and Anion 3

Neutral *E*-3

C	0.68241843360843	0.00727752505630	0.17627530945719
C	1.60050348101167	-1.12304784135495	0.40473683631869
C	1.43364012909741	-2.31830530750084	1.16550863928541
C	0.27413530995449	-2.63680473235843	1.91488276068477
H	-0.54689299549927	-1.93525417221670	1.94781569641147
C	0.18409060250412	-3.80789727306476	2.61256591679296
H	-0.70901743458376	-4.02304010503573	3.18582658616992
C	1.24745405379134	-4.73429205231510	2.60389502480282
H	1.15744214147223	-5.66512365909712	3.14927431829208
C	2.39803583655191	-4.43564040364822	1.93420011558536
H	3.23873401425740	-5.11941298842893	1.95036295839486
C	2.53428777896011	-3.21622033621767	1.22550635243757
C	3.77216424100266	-2.87233750083626	0.62408541833573
H	4.59004734431689	-3.58017772640115	0.68819052249284
C	3.95367314340657	-1.65652075904436	0.03074967489175
H	4.92072804709323	-1.37039176978697	-0.36340948617659
C	2.86201023632806	-0.77821336194968	-0.05417110671774
C	2.82685085914817	0.59835233995682	-0.54787987673977
C	3.81551816590953	1.35006675084761	-1.15867293359494
H	4.80532637622141	0.93940682941138	-1.31677450804351
C	3.50943443413997	2.63691285689656	-1.58164954833897
H	4.26988928705576	3.24475167597132	-2.05556163082234
C	2.22276076809629	3.13795011062897	-1.42245878841633
H	1.98696146345155	4.13444837333640	-1.77415678993241
C	1.22623156409590	2.37686089621175	-0.82309200799334
H	0.22942218384045	2.78413795459447	-0.74229194297843
C	1.53548601403595	1.11102366652981	-0.34308797075844
C	-0.68205559527222	-0.00254607479253	0.17439292578432
C	-1.60088807284388	1.12849148916431	0.39623305225870
C	-1.43622187640247	2.32646458768300	1.15318317013207
C	-0.27863777286992	2.64767309387259	1.90435221331034
H	0.54247189154166	1.94639532306855	1.94149948870805
C	-0.19062786286643	3.82100376058576	2.59852045657406
H	0.70098073310249	4.03825815783012	3.17331798709701
C	-1.25423031416499	4.74706083221041	2.58419539553314
H	-1.16583022697982	5.67963115158101	3.12685724875392
C	-2.40304878976856	4.44598935783152	1.91254106741629
H	-3.24396829262081	5.12958016277960	1.92444631157748
C	-2.53724881715397	3.22425883827743	1.20742969492610
C	-3.77353326715908	2.87797167514983	0.60411006919468
H	-4.59181609367885	3.58573210214229	0.66376042225959
C	-3.95325358252385	1.66004943651778	0.01454230243951
H	-4.91926764742867	1.37222815045503	-0.38092564539093
C	-2.86117606127462	0.78171765338559	-0.06454872384047
C	-2.82459855634698	-0.59668762789948	-0.55299760939794
C	-3.81164774455294	-1.35100433612697	-1.16320383635268
H	-4.80117598479897	-0.94120768102597	-1.32526259268437

C	-3.50429517892378	-2.63946662623324	-1.58032340965683
H	-4.26349924687953	-3.24940636169509	-2.05354650365465
C	-2.21792006842832	-3.13962429826063	-1.41606252967909
H	-1.98108335163746	-4.13749329312989	-1.76314915746759
C	-1.22300082381171	-2.37597993494404	-0.81728088072296
H	-0.22634178213455	-2.78278289986932	-0.73238604955662
C	-1.53367109339023	-1.10832562874218	-0.34303440740172

Neutral 3 Transition State

C	0.72404163376088	-0.03083041236985	-0.01887670070204
C	1.59836859797044	-0.83169546556239	0.76458665287733
C	1.33154993225980	-1.81221365221822	1.77654233051452
C	0.03654482103932	-2.18701326605682	2.19173607257920
H	-0.82777435809731	-1.72336817890962	1.73619703151092
C	-0.14433293667870	-3.13352345376071	3.16716176945813
H	-1.14779392865553	-3.40637968884776	3.46910774622791
C	0.96080470533446	-3.75266820167638	3.77666738803575
H	0.80524357797828	-4.49920724373650	4.54523311542734
C	2.22917010020279	-3.40873177469076	3.39425347418506
H	3.08903227926563	-3.87883056971099	3.85720726529872
C	2.44808983358199	-2.43665950464624	2.39200752764163
C	3.77284759952699	-2.08482855495804	2.00327593087929
H	4.60024137367332	-2.58159407362498	2.49592867785357
C	4.01324854142412	-1.14364762100285	1.03516976294250
H	5.02882559511082	-0.88947819789201	0.75732415328212
C	2.92935596070044	-0.51691039016883	0.41638286220953
C	2.89755345550000	0.51123889883418	-0.62408484861578
C	3.89579897379894	1.17288214284870	-1.31024121168615
H	4.94133882877240	0.96320020287362	-1.11889929805485
C	3.53150967367062	2.12664539400935	-2.26152837333072
H	4.30281778087999	2.65515058409099	-2.80769932675821
C	2.19330903276601	2.40966028514949	-2.51697065648635
H	1.93761996958822	3.15495040275799	-3.25961127055690
C	1.18180960862311	1.74766220903408	-1.82885380964280
H	0.13958614849470	1.96926243705162	-2.02660317126361
C	1.54046432435599	0.79693093746420	-0.88288038146761
C	-0.73458949959087	-0.00616728404878	-0.02346346777381
C	-1.61399245321213	0.80022173967733	0.74851463488276
C	-1.35365046301529	1.78897748950608	1.75408269187190
C	-0.06127449577962	2.16891457292810	2.17270826437287
H	0.80594943681417	1.70342260496642	1.72462167771472
C	0.11343771468181	3.12291924146512	3.14192849796856
H	1.11497773746683	3.39960003775291	3.44674697253273
C	-0.99561011369945	3.74471045962291	3.74155807380532
H	-0.84486933469802	4.49686217718946	4.50558708386900
C	-2.26157540519268	3.39611108444179	3.35540797728003
H	-3.12438016304655	3.86812177390335	3.81088807085453
C	-2.47415305528123	2.41640328248423	2.35923596822626
C	-3.79643810050237	2.05977414721987	1.96647089108348
H	-4.62698825767550	2.55875430886586	2.45151168521341
C	-4.03063415500094	1.11103588920997	1.00423014818948
H	-5.04442991361504	0.85332487090438	0.72315294790950
C	-2.94275879291019	0.48103771697859	0.39581804771145
C	-2.90422563892851	-0.55552166025312	-0.63605874295003

C	-3.89801986887888	-1.22400429318597	-1.32208990172558
H	-4.94482635829357	-1.01414084132915	-1.13802447623415
C	-3.52745084278846	-2.18527012521217	-2.26336316720165
H	-4.29511528501598	-2.71910681534694	-2.80949643792670
C	-2.18757374238762	-2.46883348411974	-2.50918311889984
H	-1.92701690271835	-3.21978827483090	-3.24438629464250
C	-1.18054505618786	-1.80002646590782	-1.82110057711064
H	-0.13702198074964	-2.02192849887215	-2.01149957561271
C	-1.54543613464177	-0.84180689829090	-0.88512058576691

Neutral *Z*-3

C	0.68922468209774	-0.00279389181445	-0.16116683034654
C	1.59217736890525	-0.40820901984780	0.93588395937578
C	1.33766582702930	-1.10542614712837	2.15621098529048
C	0.07885984866753	-1.62636964366325	2.54201967745461
H	-0.76071716644177	-1.54407606216601	1.86937352856850
C	-0.09137731951672	-2.25358798195087	3.74388058307988
H	-1.06526602284325	-2.64531761640978	4.00975396333578
C	0.98907915773603	-2.40434421721285	4.63618350649329
H	0.83854015802719	-2.88894163885232	5.59248634270151
C	2.22964272280990	-1.96944871749922	4.27025796299184
H	3.08007467759616	-2.11593529117131	4.92584314111177
C	2.44325774665571	-1.33935981343123	3.01957490447040
C	3.75656171653102	-0.98508973369021	2.61613205425020
H	4.57532084105999	-1.18015264759992	3.29855674704882
C	3.99472348421728	-0.46086706277987	1.37960202589178
H	5.00513461880658	-0.25006037142334	1.05235407722870
C	2.90231955877171	-0.18804134250855	0.54056906190080
C	2.90937704259324	0.32247748749229	-0.82954272196390
C	3.96532817979373	0.75723087502997	-1.61186809300239
H	4.98307279972142	0.71176990277776	-1.24396141649145
C	3.69303944914905	1.27743285271149	-2.87015394670276
H	4.50421501805937	1.61575522365548	-3.50249230139936
C	2.37882329359689	1.39650964599684	-3.30794969212619
H	2.17374080884432	1.83603456739932	-4.27595871979826
C	1.31963997546863	0.97338712481007	-2.51470203162660
H	0.30787577815358	1.12301185654198	-2.86292030011187
C	1.58331223499411	0.38032911833324	-1.28622216830027
C	-0.67084333329119	0.12569219363823	-0.16393679742113
C	-1.57889844174711	0.52365178969927	0.93164544894121
C	-1.33030003267350	1.21177023732736	2.15842242292803
C	-0.07332526062378	1.72951007281176	2.55448111347611
H	0.76970982859683	1.65202533698295	1.88561512779842
C	0.09100829208660	2.34792640590453	3.76173334541479
H	1.06363538801538	2.73728059122492	4.03559250180623
C	-0.99393701931287	2.49244841651490	4.64964370521045
H	-0.84811498835168	2.97016034353196	5.61013908544156
C	-2.23266254155207	2.06045408278072	4.27431105521616
H	-3.08643776966794	2.20226323923197	4.92657199897278
C	-2.44011230097607	1.43958815420012	3.01795490321142
C	-3.75152218031268	1.08882219842967	2.60543548603948
H	-4.57348416337163	1.27924128075542	3.28531703498164
C	-3.98371128086034	0.57394649078761	1.36389168255495
H	-4.99245372857920	0.36608515967047	1.02968444976951

C	-2.88721700876704	0.30687523122451	0.52836020149719
C	-2.88785312636877	-0.19402353271567	-0.84532029995468
C	-3.93998627688297	-0.62318695896331	-1.63586475404096
H	-4.95963565033025	-0.58000374137817	-1.27298747720249
C	-3.66144341868156	-1.13500646875926	-2.89622618485794
H	-4.46950864937645	-1.46886562849852	-3.53489247805028
C	-2.34509627960057	-1.25178071158843	-3.32823178964317
H	-2.13543455900280	-1.68507545718775	-4.29806138652986
C	-1.28981302618229	-0.83438430446196	-2.52683366760903
H	-0.27621017379877	-0.98234265665057	-2.87035742326981
C	-1.55961877887129	-0.24931922011183	-1.29586160400512

Anion E-3

C	0.71258533580276	-0.00341665363556	0.08758972819423
C	1.61531820282395	-1.06943051456987	0.44143310182559
C	1.42024400231754	-2.28115518286651	1.19154566587664
C	0.19598933272813	-2.64214638431799	1.79898068309503
H	-0.64745338517320	-1.97221017400872	1.71304919037369
C	0.06013022621805	-3.81284952690240	2.49872955924618
H	-0.89088540072032	-4.05885049633989	2.95538031006345
C	1.14919446997225	-4.69264856819219	2.63002324411348
H	1.03450072941002	-5.62281779845789	3.17310207310434
C	2.35925763751158	-4.35487411023717	2.08702832000621
H	3.21406945863293	-5.01173087719302	2.20551783683119
C	2.53548728337086	-3.14354448101048	1.37847617052918
C	3.82434957516104	-2.77591091831834	0.89071657010115
H	4.65172176584409	-3.45710035374316	1.05233148160623
C	4.01846527781999	-1.57515386188249	0.27151610504703
H	5.00981495554125	-1.28158883585789	-0.05590064387246
C	2.92525283652447	-0.71862693511532	0.05622455469100
C	2.88370559919084	0.59800023601588	-0.53847208511917
C	3.87742862790098	1.38729454581715	-1.11148778178077
H	4.90568274328562	1.04370906251846	-1.14248549162036
C	3.53560009671552	2.61524463477828	-1.65266483838575
H	4.29855347496937	3.24284735145755	-2.09733536209421
C	2.20220215639825	3.04408746302563	-1.64267097301109
H	1.94676101230060	4.00035043730040	-2.08416369525669
C	1.20573619270110	2.26448294481111	-1.08373491071911
H	0.17782487261193	2.60494606479790	-1.09925653694989
C	1.53890171615867	1.03787047244717	-0.49800977964442
C	-0.71185784107563	0.00775690649370	0.08566353418698
C	-1.61564623154851	1.07490842814687	0.43346546545821
C	-1.42276165344948	2.28936661057177	1.17979645789738
C	-0.20024793821472	2.65278605897163	1.78931173630296
H	0.64360228219673	1.98278236563819	1.70799440859954
C	-0.06661255639346	3.82589101910388	2.48549816367416
H	0.88304929189052	4.07366428740966	2.94401041291562
C	-1.15625337242974	4.70585237530607	2.61079500924427
H	-1.04329625848936	5.63787443073722	3.15106042793698
C	-2.36473270850199	4.36591586268266	2.06563068310866
H	-3.22004774924262	5.02293666589384	2.17954316415567
C	-2.53871472029986	3.15211939198184	1.36076536453469
C	-3.82624864496084	2.78242313720406	0.87107617211117
H	-4.65417838168936	3.46400276425719	1.02813101906416

C	-4.01854427754463	1.57933845901372	0.25587418547176
H	-5.00899824445554	1.28427464920365	-0.07289559196361
C	-2.92460604273074	0.72230537050083	0.04650000140820
C	-2.88137296577618	-0.59653604307559	-0.54309874397110
C	-3.87365862240479	-1.38830374977544	-1.11518200488038
H	-4.90193822055214	-1.04512794239416	-1.14963048494512
C	-3.53034799477073	-2.61826734472478	-1.65084300399516
H	-4.29219065267086	-3.24788430732174	-2.09458158398958
C	-2.19683940986702	-3.04661538961597	-1.63644917153090
H	-1.94010463408624	-4.00449183210292	-2.07366262070317
C	-1.20179643558365	-2.26456782835409	-1.07838190631460
H	-0.17377130853955	-2.60481900199505	-1.09053439187470
C	-1.53657350482787	-1.03587288407767	-0.49798919815273

Anion 3 Transition State

C	0.72958105970722	-0.01028292375509	-0.04244252002356
C	1.60649599527133	-0.81883124051127	0.74394322615750
C	1.33003306184303	-1.80225846154529	1.75089964093749
C	0.02672467012251	-2.16933846330927	2.15426152252594
H	-0.81992317818829	-1.68845769810505	1.68320546721418
C	-0.16803547075563	-3.11757661118583	3.12493741493157
H	-1.17595584905550	-3.38446974809299	3.41818534450785
C	0.92797962295088	-3.74732720609191	3.74223532248855
H	0.76093191374406	-4.49542057589043	4.50731563741319
C	2.20195771366088	-3.41139783327265	3.37213350027535
H	3.05444827753951	-3.88928845271909	3.84144671390300
C	2.43711481280798	-2.43705291093046	2.37475553163938
C	3.76783166262094	-2.09265858786281	1.99961621927407
H	4.58848712633784	-2.59609061496993	2.49728954095606
C	4.01897739223345	-1.14884078160335	1.03797891729054
H	5.03829061231449	-0.89811502035242	0.76908417657607
C	2.94198971327673	-0.51312527929977	0.41236926304923
C	2.92656632167230	0.52049493964629	-0.62293013924976
C	3.93561118187316	1.18018833071545	-1.29770818549175
H	4.97876742659855	0.96354433222973	-1.09883322960088
C	3.58579765020706	2.14077702403620	-2.24685884777728
H	4.36464536786663	2.66784973559932	-2.78427016532293
C	2.24971615769058	2.43194335638396	-2.51140006815024
H	2.00426505886118	3.18257520533205	-3.25267506822413
C	1.23048575144975	1.77098289570398	-1.83440283696502
H	0.18903482250383	1.99414573567262	-2.03517794583413
C	1.57204584639387	0.81267295325888	-0.88908343039537
C	-0.73323966268131	0.01591793383611	-0.05232847712895
C	-1.61181435670080	0.79515017778602	0.73100445687926
C	-1.37059232588347	1.79363857786386	1.74486916867520
C	-0.07876336677112	2.19106476231053	2.14926491855335
H	0.78393756019098	1.73107171957741	1.68433341415890
C	0.11278138556073	3.14594794285667	3.11830867851926
H	1.11799399759663	3.42891107025286	3.40659926634256
C	-0.99306849252790	3.75332246962442	3.73270348107443
H	-0.84379051586854	4.50718198102490	4.49654463773061
C	-2.26241712411887	3.38748405646227	3.36124887238056
H	-3.12147916638357	3.85300990393638	3.83285536403151
C	-2.49005659513909	2.40836110891270	2.36941898824329

C	-3.82400067947168	2.03861070455752	1.99940729885184
H	-4.65329006118031	2.52949251771556	2.49567552442192
C	-4.04305199260949	1.08668358235532	1.04734128544104
H	-5.05961344098093	0.81353832016100	0.78103681858202
C	-2.95933962379075	0.45205061287301	0.40709928073666
C	-2.91712022699175	-0.56118369380522	-0.59699027443532
C	-3.91264497107853	-1.26945087532630	-1.28370205854315
H	-4.96117076174406	-1.07321662261954	-1.08222325798585
C	-3.55547793123038	-2.22155855232364	-2.21605737340201
H	-4.32172556441883	-2.77248327103334	-2.74890290826378
C	-2.19477931625611	-2.48699275263890	-2.48102321917534
H	-1.93576970823710	-3.24210698863947	-3.21500474643313
C	-1.19552444213223	-1.80432651429688	-1.82423641072826
H	-0.15325563698286	-2.01980549315496	-2.03840517042387
C	-1.53553170171701	-0.82135477734917	-0.87150256020769

Anion Z-3

C	0.72155842489640	0.02690078659359	-0.07290080934840
C	1.63819072616668	-0.46761514548366	0.92527981374920
C	1.42752609409372	-1.18750078056294	2.15255286417019
C	0.15923196933634	-1.61241420146720	2.60924868114150
H	-0.71014606912409	-1.40691239247128	2.00254250776843
C	0.01023849082806	-2.28292596662335	3.79503585184184
H	-0.97612156317821	-2.59580110486834	4.11534559569923
C	1.13084020659766	-2.56970197424210	4.59441924768824
H	1.00824312483281	-3.08979524802906	5.53665600472367
C	2.37773855724602	-2.20420824731866	4.16483945203685
H	3.25220456636456	-2.44168822178448	4.76076066332022
C	2.56433868130469	-1.52726772828905	2.93697801217281
C	3.87888772520270	-1.21684442016408	2.48021124733858
H	4.72185725682772	-1.49151961755630	3.10365774368769
C	4.07171164960747	-0.61808171621322	1.26951011994776
H	5.07546476500194	-0.41969422890542	0.91002599964512
C	2.96087512106027	-0.25197518798114	0.48961438124708
C	2.91385895087345	0.36733847716397	-0.81468345818920
C	3.92287534323179	0.82213895424076	-1.66011009215026
H	4.96586550361400	0.70627144421131	-1.38618418563318
C	3.57854203341109	1.43726983220552	-2.85160081992124
H	4.35136393606638	1.79236542721380	-3.52265671410551
C	2.23035742410236	1.62085257484823	-3.18619657058711
H	1.97485726346733	2.12517259565996	-4.11085868309902
C	1.22099388023834	1.17954608237677	-2.35042589063569
H	0.18500356515422	1.35237499492084	-2.61471687485964
C	1.54971480256331	0.51425516223295	-1.16256534641423
C	-0.70289386878821	0.09721899676897	-0.07582170852724
C	-1.62456235458783	0.58248920970949	0.92232975812633
C	-1.42049022264011	1.29184538283762	2.15688726216716
C	-0.15497761531619	1.71453964313664	2.62325491411991
H	0.71775502458082	1.51553317519828	2.01929034414027
C	-0.01267584015416	2.37542631596146	3.81529996618463
H	0.97168614350967	2.68724351202258	4.14270505913407
C	-1.13737707686062	2.65407128666258	4.61171266959461
H	-1.01999339956274	3.16657456978253	5.55875981135893
C	-2.38169943858449	2.29062356396001	4.17304958942989

H	-3.25942297035081	2.52207614209920	4.76653789779195
C	-2.56147225831081	1.62385535385387	2.93862123575439
C	-3.87353066660254	1.31664624121664	2.47265875240194
H	-4.71972686172121	1.58570109125237	3.09419427435888
C	-4.05998069637788	0.72850141024992	1.25578071456425
H	-5.06167471852418	0.53304198524465	0.88902900167665
C	-2.94497912754679	0.36978206561539	0.47846131990005
C	-2.89145918110340	-0.23781481875094	-0.83113598665753
C	-3.89611960834898	-0.68615346576902	-1.68513444926220
H	-4.94052862674228	-0.57380677821344	-1.41517417146115
C	-3.54560330845913	-1.29047769786555	-2.88037152073191
H	-4.31497558868891	-1.64047279574630	-3.55803503725566
C	-2.19575241317252	-1.47007191611141	-3.21025829539278
H	-1.93554350624555	-1.96586229772677	-4.13822090931237
C	-1.19071195044070	-1.03538060207824	-2.36585452356460
H	-0.15333181604777	-1.20524705343198	-2.62655099555099
C	-1.52565048269970	-0.38075266958597	-1.17382371422241

References

[1] Matsuo, Y.; Wang, Y.; Ueno, H.; Nakagawa, T.; Okada, H. *Angew. Chem. Int. Ed.* **2019**, *58*, 8762–8767.

[2] Zhang, X.; Han, J.-B.; Li, P.-F.; Ji, X.; Zhang, Z. *Synth. Commun.* **2009**, *39*, 3804–3815.

[3] Fulmer, G. R.; Miller, A. J. M.; Sherden, N. H.; Gottlieb, H. E.; Nudelman, A.; Stoltz, B. M.; Bercaw, J. E.; Goldberg, K. I. *Organometallics* **2010**, *29*, 2176–2179.

[4] Pijper, T. C.; Pijper, D.; Pollard, M. M.; Dumur, F.; Davey, S. G.; Meetsma, A.; Feringa, B. L. *J. Org. Chem.* **2010**, *75*, 825–838.

[5] Rakstys, K.; Saliba, M.; Gao, P.; Gratia, P.; Kamarauskas, E.; Paek, S.; Jankauskas, V.; Nazeeruddin, M. K. *Angew. Chem. Int. Ed.* **2016**, *55*, 7464–7468.

[6] Assadi, N.; Pogodin, S.; Cohen, S.; Agranat, I. *Struct. Chem.* **2013**, *24*, 1229–1240.

[7] Bauer, J.; Hou, L.; Kistemaker, J. C. M.; Feringa, B. L. *J. Org. Chem.* **2014**, *79*, 4446–4455.

[8] Thrippleton, M. J.; Keeler, J. *Angew. Chem. Int. Ed.* **2003**, *42*, 3938–3941.

[9] Perrin, C. L.; Engler, R. E. *J. Magn. Reson. A* **1996**, *123*, 188–195.

[10] Ugras, T. J.; Yao, Y.; Robinson, R. D. *Chirality* **2023**, *35*, 846–855.

[11] Schlenk, W.; Bergmann, E. *Justus Liebigs Ann. Chem.* **1928**, *463*, 1–97.

[12] Cox, R. H. *J. Magn. Reson.* **1970**, *3*, 223–229.

[13] Walczak, M.; Stucky, G. D. *J. Organometal. Chem.* **1975**, *97*, 313–323.

[14] Eaton, G. R.; Eaton, S. S.; Barr, D. P.; Weber, R. T. *Quantitative EPR*; Springer-Verlag/Wien: New York, 2010.

[15] Stoll, S.; Schweiger, A. *J. Magn. Reson.* **2006**, *178*, 42–55.

[16] Otero, M.; Roman, E.; Samuel, E.; Gourier, D. *J. Electroanal. Chem.* **1992**, *325*, 143–152.

[17] Chippendale, J. C.; Gill, P. S.; Warhurst, E. *Trans. Faraday Soc.* **1967**, *63*, 1088–1096.

[18] Lewis, I. C.; Singer, L. S. *J. Chem. Phys.* **1966**, *44*, 2082–2085.

[19] Muus, L. T., Atkins, P. W., Eds. *Electron Spin Relaxation in Liquids*; Springer US: Boston, MA, 1972.

[20] Biller, J. R.; Elajaili, H.; Meyer, V.; Rosen, G. M.; Eaton, S. S.; Eaton, G. R. *J. Magn. Reson.* **2013**, *236*, 47–56.

[21] Mailer, C.; Nielsen, R. D.; Robinson, B. H. *J. Phys. Chem. A* **2005**, *109*, 4049–4061.

[22] Robinson, B. H.; Haas, D. A.; Mailer, C. *Science* **1994**, *263*, 490–493.

[23] Meyer, V.; Eaton, S. S.; Eaton, G. R. *Appl. Magn. Reson.* **2014**, *45*, 993–1007.

[24] Biller, J. R.; McPeak, J. E.; Eaton, S. S.; Eaton, G. R. *Appl. Magn. Reson.* **2018**, *49*, 1235–1251.

[25] Owenius, R.; Eaton, G. R.; Eaton, S. S. *J. Magn. Reson.* **2005**, *172*, 168–175.

[26] Engler, B. P.; Harrah, L. A. *Viscosity and Density of 2-Methyltetrahydrofuran as a Function of Temperature.*; 1979.

[27] Metz, D. J.; Glines, A. *J. Phys. Chem.* **1967**, *71*, 1158–1158.

[28] Archibald, E. H.; Ure, W. *J. Chem. Soc.* **1927**, 610–614.

[29] Lange, N. A. *Lange's Handbook of Chemistry*, 10th ed.; McGraw-Hill: New York, 1923; pp 1657–1669.

[30] Hwang, J. S.; Mason, R. P.; Hwang, L. P.; Freed, J. H. *J. Phys. Chem.* **1975**, *79*, 489–511.

[31] Santanni, F.; Albino, A.; Atzori, M.; Ranieri, D.; Salvadori, E.; Chiesa, M.; Lunghi, A.; Bencini, A.; Sorace, L.; Totti, F.; Sessoli, R. *Inorg. Chem.* **2021**, *60*, 140–151.

[32] de Camargo, L. C.; Briganti, M.; Santana, F. S.; Stinghen, D.; Ribeiro, R. R.; Nunes, G. G.; Soares, J. F.; Salvadori, E.; Chiesa, M.; Benci, S.; Torre, R.; Sorace, L.; Totti, F.; Sessoli, R. *Angew. Chem. Int. Ed.* **2021**, *60*, 2588–2593.

[33] Kazmierczak, N. P.; Mirzoyan, R.; Hadt, R. G. *J. Am. Chem. Soc.* **2021**, *143*, 17305–17315.

[34] Rengan, S. K.; Khakhar, M. P.; Prabhananda, B. S.; Venkataraman, B. *Pramana* **1974**, *3*, 95–121.

[35] Biller, J. R.; Meyer, V.; Elajaili, H.; Rosen, G. M.; Kao, J. P. Y.; Eaton, S. S.; Eaton, G. R. *J. Magn. Reson.* **2011**, *212*, 370–377.

[36] Zhou, A.; Sun, Z.; Sun, L. *Innovation* **2024**, *5*, 100662.

[37] Neese, F. *WIREs Comput. Mol. Sci.* **2018**, *8*, e1327.

[38] Neese, F. *WIREs Comput. Mol. Sci.* **2012**, *2*, 73–78.

[39] Ganyushin, D.; Neese, F. *J. Chem. Phys.* **2006**, *125*, 024103.

[40] Stoychev, G. L.; Auer, A. A.; Neese, F. *J. Chem. Theory Comput.* **2017**, *13*, 554–562.

[41] Kollmar, C.; Sivalingam, K.; Helmich-Paris, B.; Angeli, C.; Neese, F. *J. Comput. Chem.* **2019**, *40*, 1463–1470.

[42] Lang, L.; Neese, F. *J. Chem. Phys.* **2019**, *150*, 104104.

[43] Kollmar, C.; Sivalingam, K.; Guo, Y.; Neese, F. *J. Chem. Phys.* **2021**, *155*, 234104.

[44] Guo, Y.; Sivalingam, K.; Neese, F. *J. Chem. Phys.* **2021**, *154*, 214111.

[45] Neese, F. *J. Comput. Chem.* **2023**, *44*, 381–396.

[46] Ugandi, M.; Roemelt, M. *Int. J. Quantum Chem.* **2023**, *123*, e27045.

[47] Chantzis, A.; Laurent, A. D.; Adamo, C.; Jacquemin, D. *J. Chem. Theory Comput.* **2013**, *9*, 4517–4525.

[48] Forno, A. E. J.; Peover, M. E.; Wilson, R. *Trans. Faraday Soc.* **1970**, *66*, 1322–1333.

[49] Reddoch, A. H. *J. Chem. Phys.* **1965**, *43*, 225–234.

[50] Nishiuchi, T.; Ito, R.; Takada, A.; Yasuda, Y.; Nagata, T.; Stratmann, E.; Kubo, T. *Chem. Asian J.* **2019**, *14*, 1830–1836.

[51] Maki, A. H.; Allendoerfer, R. D.; Danner, J. C.; Keys, R. T. *J. Am. Chem. Soc.* **1968**, *90*, 4225–4231.

[52] Zaitsev, V.; Rosokha, S. V.; Head-Gordon, M.; Kochi, J. K. *J. Org. Chem.* **2006**, *71*, 520–526.

[53] Bonačić-Koutecký, V.; Koutecký, J.; Michl, J. *Angew. Chem. Int. Ed. Engl.* **1987**, *26*, 170–189.

[54] Harris, F. E. *Int. J. Quantum Chem.* **2002**, *88*, 701–734.
